# Polyoxazoline‐Based Nanovaccine Synergizes with Tumor‐Associated Macrophage Targeting and Anti‐PD‐1 Immunotherapy against Solid Tumors

**DOI:** 10.1002/advs.202300299

**Published:** 2023-07-11

**Authors:** Ana I. Matos, Carina Peres, Barbara Carreira, Liane I. F. Moura, Rita C. Acúrcio, Theresa Vogel, Erik Wegener, Filipa Ribeiro, Marta B. Afonso, Fábio M. F. Santos, Águeda Martínez‐Barriocanal, Diego Arango, Ana S. Viana, Pedro M. P. Góis, Liana C. Silva, Cecília M. P. Rodrigues, Luis Graca, Rainer Jordan, Ronit Satchi‐Fainaro, Helena F. Florindo

**Affiliations:** ^1^ Grouf of BioNanoSciences ‐ Drug Delivery and Immunoengineering, Research Institute for Medicines (iMed.ULisboa), Department of Pharmacy, Pharmacology and Health Technologies Faculty of Pharmacy Universidade de Lisboa Lisbon 1649‐003 Portugal; ^2^ Faculdade de Medicina, Instituto de Medicina Molecular João Lobo Antunes, Lisbon Academic Medical Center Universidade de Lisboa Lisbon 1649‐028 Portugal; ^3^ Department of Chemistry, Faculty of Chemistry and Food Chemistry, School of Science Technische Universität Dresden 01062 Dresden Germany; ^4^ Group of Biomedical Research in Digestive Tract Tumors CIBBIM‐Nanomedicine Vall d'Hebron Research Institute (VHIR) Universitat Autònoma de Barcelona (UAB) Barcelona 08035 Spain; ^5^ Group of Molecular Oncology Lleida Biomedical Research Institute (IRBLleida) Lleida 25198 Spain; ^6^ Centro de Química Estrutural Departamento de Química e Bioquímica Institute of Molecular Sciences Faculty of Sciences Universidade de Lisboa Lisbon 1749‐016 Portugal; ^7^ Department of Physiology and Pharmacology Faculty of Medicine Sagol School of Neuroscience Tel Aviv University Tel Aviv 69978 Israel

**Keywords:** anti‐PD‐1, nanovaccines, poly(2‐oxazoline)s, tumor immune microenvironment, tumor‐associated macrophages

## Abstract

Immune checkpoint blockade reaches remarkable clinical responses. However, even in the most favorable cases, half of these patients do not benefit from these therapies in the long term. It is hypothesized that the activation of host immunity by co‐delivering peptide antigens, adjuvants, and regulators of the transforming growth factor (TGF)‐β expression using a polyoxazoline (POx)‐poly(lactic‐*co*‐glycolic) acid (PLGA) nanovaccine, while modulating the tumor‐associated macrophages (TAM) function within the tumor microenvironment (TME) and blocking the anti‐programmed cell death protein 1 (PD‐1) can constitute an alternative approach for cancer immunotherapy. POx‐Mannose (Man) nanovaccines generate antigen‐specific T‐cell responses that control tumor growth to a higher extent than poly(ethylene glycol) (PEG)‐Man nanovaccines. This anti‐tumor effect induced by the POx‐Man nanovaccines is mediated by a CD8^+^‐T cell‐dependent mechanism, in contrast to the PEG‐Man nanovaccines. POx‐Man nanovaccine combines with pexidartinib, a modulator of the TAM function, restricts the MC38 tumor growth, and synergizes with PD‐1 blockade, controlling MC38 and CT26 tumor growth and survival. This data is further validated in the highly aggressive and poorly immunogenic B16F10 melanoma mouse model. Therefore, the synergistic anti‐tumor effect induced by the combination of nanovaccines with the inhibition of both TAM‐ and PD‐1‐inducing immunosuppression, holds great potential for improving immunotherapy outcomes in solid cancer patients.

## Graphical abstract

Remodeling of a tumor immunosuppressive microenvironment via a polyoxazoline‐poly(lactic‐*co*‐glycolic) acid nanovaccine combined with modulators of αCSF‐1R and αPD‐1 controls tumor growth and prolongs survival of three murine solid tumor models (CT26, MC38, and B16F10), thus constituting a promising approach to improve cancer immunotherapy.

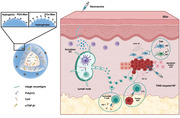

## 1 Introduction

Personalized cancer immunotherapeutic approaches have been explored as potentiators of anti‐tumor immunity to improve the outcomes in solid tumors such as melanoma, lung cancer, and colorectal cancer (CRC).

**Figure 1:**
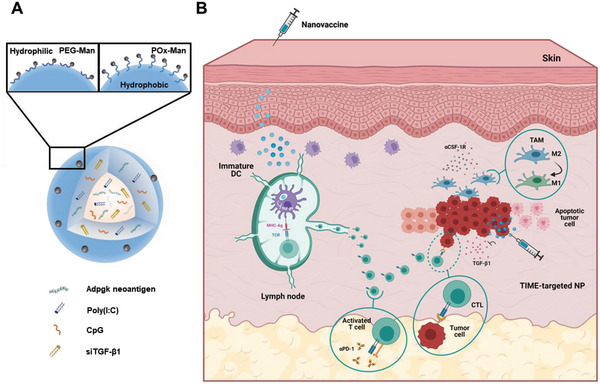
Design of multifunctional nanoparticles (NP) to deliver combinations of neoantigen epitopes and immune modulators against solid tumors. A) NP composed of a polymer core of PLGA and (i) mannosylated polymers (PLGA‐PEG‐Man or POx‐Man) for antigen‐presenting cell (APC) targeting; and (ii) tripeptide motif Arg–Gly–Asp (RGD)‐modified POx (POx‐RGD) polymers to target tumor‐immune microenvironment (TIME). POx‐Man NP delivers combinations of major histocompatibility complex (MHC) class I and MHC class II Adpgk neoantigens, CpG and Poly(I:C). A siRNA targeting TGF‐β1 (siTGF‐β1) is delivered by NP to modulate the secretion of this immunosuppressive player by dendritic cells (DC) (POx‐Man NP) or at the TIME (POx‐RGD NP). B) APC‐targeted NP are efficiently internalized by immature DC promoting the delivery of neoantigens and immune regulators, which improves DC maturation, and neoantigen‐specific CD8^+^ cytotoxic T‐lymphocytes (CTL) and CD4^+^ T‐cell responses. Activated CD4^+^ T cells promote the expansion of effector CTL, which migrate and induce the destruction of tumor cells expressing Adpgk antigens. Combination of therapeutic cancer vaccines with modulators of the immunosuppressive TIME (POx‐RGD NP, αCSF‐1R, and αPD‐1) to control tumor growth and improve survival.

Recent advances focused on efficiently delivering combinations of synergistic immunomodulators, such as adjuvants and cancer antigens, to enhance tumor immunogenicity, have made use of poly(ethylene glycol) (PEG)‐based nanovaccines.^[^ ^1^ ^]^ PEG is widely used, but the presence of anti‐PEG antibodies was recently reported due to its potential immunogenicity and antigenicity.^[^ ^2^ ^]^ These adverse events prompted the evaluation of potential alternatives to PEG, such as the poly(2‐oxazoline)s (POx).^[^ ^3^ ^]^ These are amphiphilic polymers, which physical properties and chemical functions can be tailored to their application. Here we set out to develop and characterize a therapeutic nanovaccine made of poly(lactic‐*co*‐glycolic) acid (PLGA) nanoparticles (NP) modified with mannose‐functionalized diblock‐POx (POx‐Man) (**Figure** **1**) for antigen‐presenting cell (APC) targeting, as an alternative to mannosylated PLGA‐PEG (PEG‐Man) nanovaccines. POx‐Man NP were designed to enable the in vivo co‐delivery of ADP‐dependent glucokinase (Adpgk) (MC38 neoantigen) peptides, toll‐like receptor (TLR) agonists (CpG and polyinosinic:polycytidylic acid [Poly(I:C)]), and small interfering RNA (siRNA) targeting dendritic cell (DC)‐related immunosuppressive agents. We show that POx‐Man nanovaccines induced stronger immune responses, which led to significant tumor growth inhibition when compared with PEG‐Man NP.

Combination immunotherapies are emerging as a key trend in cancer treatment by targeting and modulating the function of multiple cell players and signaling pathways related to tumor immune evasion. The infiltration of tumor‐associated macrophages (TAM) has been correlated with poor prognosis for most solid tumors, including melanoma and CRC.^[^ ^4^ ^]^

Pro‐tumorigenic TAM trigger tumor development, angiogenesis, and therapeutic resistance mediated by the overexpression of immune suppressive cytokines, the differentiation of T regulatory (Treg) cells, and the downregulation of major histocompatibility complex (MHC) class II (MHCII) molecules.^[^ ^5^ ^]^ TAM infiltration in several solid tumors has been counteracted by disrupting the colony‐stimulating factor 1 (CSF‐1)/CSF‐1 receptor (CSF‐1R) pathway, which is strongly correlated to macrophage survival and differentiation.^[^ ^6^ ^]^ Transforming growth factor (TGF)‐β signaling is also associated with poor prognosis in patients with metastatic CRC (mCRC)^[^ ^7^ ^]^ and melanoma,^[^ ^8^ ^]^ among others. It has been shown that TGF‐β downregulation induced potent cytotoxic immune responses and prevented metastasis.^[^ ^9^ ^]^ We, therefore, hypothesized that modulators of the TGF‐β1 secretion and TAM function could unlock the full potential of our POx nanovaccine and thereby potentiate the immune‐mediated destruction of solid tumors. To test this hypothesis, we explored the potential synergistic effect obtained using pexidartinib, a CSF‐1R inhibitor that modulates TAM, and the multifunctional POx‐Man NP entrapping combinations of siRNA targeting TGF‐β1 (siTGF‐β1), TLR ligands, and Adpgk peptide epitopes, as model neoantigens expressed in the MC38 cell line of CRC. The tripeptide motif Arg–Gly–Asp (RGD)‐modified POx (POx‐RGD) NP was used to evaluate the potential added value of delivering the siTGF‐β1 and the immune potentiators CpG and Poly(I:C) to the tumor‐immune microenvironment (TIME) (Figure 1).

Moreover, the programmed cell death ligand 1 (PD‐L1) is highly expressed in tumor cells and APC,^[^ ^10^ ^]^ and its upregulation is associated with the suppression of the synergic T cell receptor‐CD8 cooperativity, which delays the recognition of the antigen by CD8 T cells.^[^ ^11^ ^]^ The programmed cell death protein 1 (PD‐1) antibody revolutionized the treatment landscape of metastatic melanoma patients and was more recently approved for a specific subset of CRC patients. However, around 50% and 30% response rates have been obtained for advanced melanoma and CRC patients, respectively, who generally suffer grade 3/4 side effects.^[^ ^12^ ^]^ Therefore, alternative approaches are needed to make immunotherapy relevant for most patients diagnosed with advanced solid tumors. To address this challenge, we evaluated whether a combinational nano‐immunotherapy modulating the TIME via POx‐Man nanovaccine, anti‐PD‐1 monoclonal antibody (mAb), and TAM targeting, would control MC38 and CT26 tumor growth and survival. Our data show that the combination of our cancer nanovaccine with modulators of the immunosuppressive TIME (αCSF‐1R, and αPD‐1) constitutes a promising nanotechnology‐enhanced immunotherapy against solid tumors. In fact, this data was further validated in B16F10‐bearing mice, a highly aggressive melanoma model for immunotherapy studies.^[^ ^13^ ^]^

## 2 Results

### 2.1 Polyoxazoline Nanoparticles as Anti‐Tumor Nanovaccines

Polymeric nanovaccines were synthesized to deliver combinations of modulators of DC function, namely neoantigens, TLR ligands, and siTGF‐β1 signaling pathway. To potentiate the interaction of nanovaccines with DC, mannose‐functionalized NP were developed to target the mannose receptor (CD206) expressed at the DC surface, thus promoting receptor–ligand interaction and subsequently improving payload delivery.^[^ ^14^ ^]^ Moreover, we explored POx (Figure S1A,B, Supporting Information) as an alternative to PEG on NP surface, taking advantage of its hydrophilicity, while addressing the recently raised concerns on the secretion of anti‐PEG antibodies.^[^ ^2^ ^]^ The presence of mannose in the in‐house synthesized mannose‐grafted PLGA‐PEG polymer (PLGA‐PEG‐Man) was confirmed by the multiplet signal between 3.7 and 4.2 ppm in ^1^H‐NMR spectra (Figure S1C, Supporting Information), as reported by Alonso‐Sande et al.^[^ ^15^ ^]^ The multiplet signal between 5.9 and 6.7 ppm indicates the conjugation between the mannosamine group and the Boc‐PEG‐amine, using the homobifunctional cross‐linker BS^3^ (Figure S1C, Supporting Information).^[^ ^16^ ^]^ The degree of labeling (DoL) of 18.5% for mannose‐grafted POx polymer (POx‐Man) was assessed by 3,5‐dinitrosalicylic acid (DNS) assay, as the mannose end group was undetectable in the ^1^H‐NMR spectra (Figure S1D,F, Supporting Information).

Non‐targeted (no targeting moiety) and APC‐targeted (PLGA‐PEG‐Man and PLGA‐POx‐Man) NP presented an average hydrodynamic diameter close to 200 nm, with low polydispersity index (PdI) (<0.2) and near‐neutral surface charge, depending on NP composition and entrapped bioactive molecules (**Figure** **2**A and Table S1, Supporting Information).

Figure 2The co‐delivery of antigens and adjuvants by POx‐Man nanovaccines strongly inhibits tumor growth in MC38‐bearing mice by enhancing the systemic activation of T lymphocytes and the secretion of Th1 cytokines, being strongly mediated by CD8^+^ T cells. A) Dynamic light scattering analysis and B,C) atomic force microscopy images (topography: left; phase: right) show a uniform size polydispersity for slight roughness spherical PLGA‐PEG‐Man (B) and PLGA‐POx‐Man (C) nanoparticles (NP), empty and loaded with the therapeutic agents. D) Percentage of antigen‐presenting cells (APC), CD11b^+^CD11c^+^, CD11b^+^CD11c^−^, and CD11b^−^CD11c^+^ cells, with internalized Adpgk‐loaded PLGA‐POx‐Man or PLGA‐PEG‐Man NP, 14 h after immunization. E) Expression of surface activation and maturation markers on activated CD11b^+^CD11c^+^MHCII^+^ cells in the lymph nodes, with and without internalized Adpgk‐loaded NP, 14 h after immunization of C57BL/6J mice. Data are presented as the mean ± s.d., *n* = 3. Statistical significance was calculated by two‐way analysis of variance (ANOVA) with Tukey multiple comparisons post‐hoc test. F) Immunization scheme of C57BL/6J mice to evaluate T‐cell activation ex vivo. G–I) The activation of CD4 (G), CD8 (H), and CTL (I) is enhanced by the co‐delivery of antigens and adjuvants by POx‐Man nanovaccine. J–P) Secretion of IFN‐γ (J,M), IL‐2 (K,N), TNF‐α (L,O), and IL‐10 (P) by CD8^+^ and CD4^+^ T cells after re‐stimulation of splenocytes in culture with relevant peptides for 6 h. The highest levels of the triad IFN‐γ, IL‐2, and TNF‐α induced by Adpgk‐loaded POx‐Man nanovaccine predict an improved cytotoxic CD8^+^/Th1 T‐cell activity. This nanovaccine also modulated the Th2 cytokine secretion profile, while inducing lower levels of CD4^+^ T cells expressing IL‐10 when compared to PEG‐Man nanovaccine. Data are presented as the mean ± s.d., *n* = 3. Statistical significance was calculated by one‐way ANOVA followed by Tukey multiple comparisons post‐hoc test. Q) C57BL/6J mice were inoculated subcutaneously with 0.5 × 10^6^ MC38 tumor cells and treated with Adpgk‐loaded PEG‐Man and POx‐Man nanovaccines on days 7 and 14 in combination with αCD8 monoclonal antibody (mAb) (10 mg kg^−1^), on days 6, 9, 12, and 15. R) Body weight change is expressed as the percent change in weight from the day of treatment initiation. S) Average MC38 tumor growth curves. T) Individual MC38 tumor volumes at day 20 following tumor inoculation. The data are presented as mean ± s.e.m of MC38‐bearing mice (*n* = 5 animals), replicated in two independent experiments for PBS, PLGA‐POx‐Man, and PLGA‐PEG‐Man groups. Statistical significance was analyzed by one‐way analysis of variance (ANOVA) followed by Tukey multiple comparisons post‐hoc test and *p* values correspond to tumor volume at day 20 after tumor inoculation, compared to the PLGA‐POx‐Man nanovaccine group.

**Figure:**
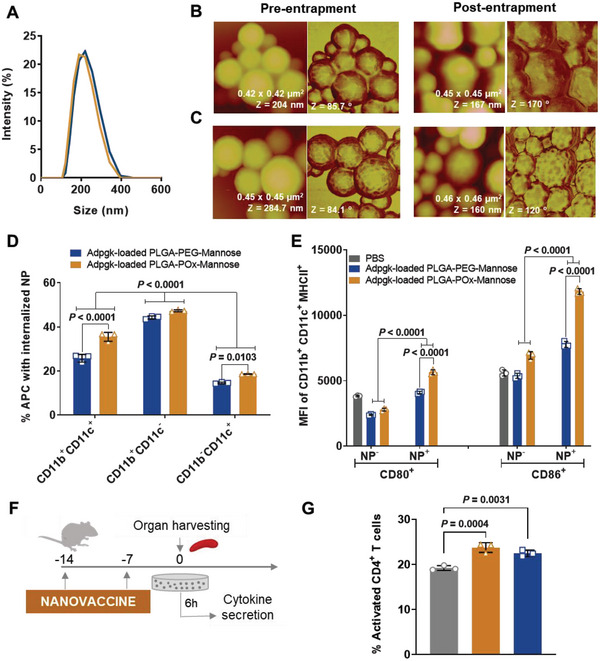

**Figure:**
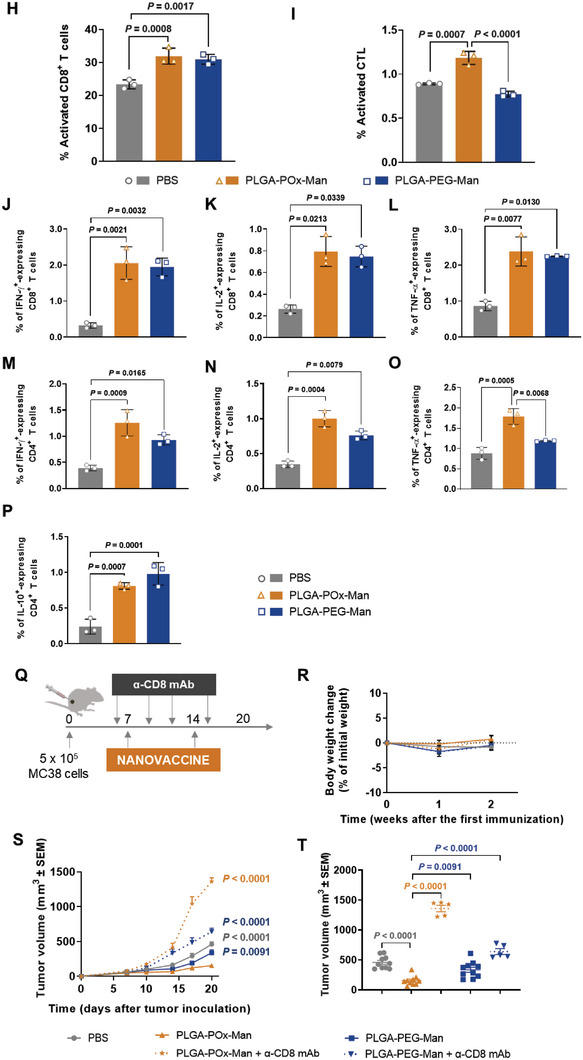

Atomic force microscopy (AFM) images showed homogenous spherical‐shaped populations with a slightly roughness surface, which diameters correlate with the ones measured by dynamic light scattering (DLS) (Figure 2B,C).

DC‐targeted NP displayed high levels of entrapment efficiency (EE) and loading capacity (LC) for the Adpgk_R304M_ MHC class I‐restricted (MHCI‐Adpgk) and MHC class II‐restricted (MHCII‐Adpgk) peptides (Table S1, Supporting Information), to enable the neoantigen presentation by MHCI and MHCII pathways, respectively, and the subsequent engagement of both CD4^+^ and CD8^+^ T cells.^[^ ^17^ ^]^ The EE obtained for the MHCII‐Adpgk peptide (EE > 70.5 ± 1.4% and LC > 35.2 ± 0.7 µg mg^−1^) was higher than the one obtained for the MHCI‐Adpgk peptide (EE > 55.6 ± 1.1% and LC 27.8 ± 0.6 µg mg^−1^) independently from the NP polymeric composition. DC‐targeted NP capacity to co‐incorporate the oligonucleotides such as the immune adjuvants CpG (EE > 80.5 ± 10.9% and LC > 8.1 ± 1.1 µg mg^−1^) and Poly(I:C) (EE > 83.1 ± 10.6% and LC > 16.6 ± 2.1 µg mg^−1^), alone or in combination with the siTGF‐β1 (EE > 96.1 ± 2.5% and LC > 30.8 ± 0.8 µg mg^−1^) was not affected by the entrapment of neoantigens (Table S1, Supporting Information).

Empty (no cargo), non‐targeted, and DC‐targeted NP presented a potential biocompatible and safe profile, as both formulations did not affect DC viability (>87%) for the longest incubation time tested, independently from the NP polymeric composition (Figure S2A,C,F, Supporting Information).

PLGA‐PEG‐Man (20% m/m) NP were internalized at a higher extent than those comprising 10% and 30% m/m of mannose‐grafted polymers (Figure S2B,F, Supporting Information). NP synthesized using 20% m/m of mannose‐grafted polymers (PLGA‐PEG‐Man and PLGA‐POx‐Man) presented higher internalization levels (*p* < 0.0001) by murine immature DC (JAWSII) than non‐targeted NP (Figure S2B,D,F, Supporting Information), which reveals a potential stronger interaction between the mannosylated NP and the mannose receptor (CD206). Therefore, similarly to what we previously obtained using mannose‐grated PLGA‐based NP,^[^ ^18^ ^]^ nanoparticulate systems prepared using 20% m/m PLGA‐PEG‐Man or PLGA‐POx‐Man were selected for subsequent in vivo therapeutic efficacy studies. Confocal microscopy images confirm that PLGA‐POx‐Man NP are internalized by DC (Figure S2E, Supporting Information).

PLGA‐POx‐Man NP were preferentially internalized in vivo by CD11b^+^CD11c^+^ and CD11b^+^CD11c^−^ cells, compared to CD11b^−^CD11c^+^ cells (Figure 2D and Figure S3A,B, Supporting Information), due to their high phagocytic capacity, in addition to the expected ability to efficiently detect foreign NP.^[^ ^19^ ^]^

PLGA‐POx‐Man NP induced a significantly higher expression (*p* < 0.0001) of the co‐stimulatory/maturation markers CD80/86 on the surface of activated circulating DC (Figure 2E and Figure S3C, Supporting Information). In fact, the co‐delivery of antigens and adjuvants by PLGA‐POx‐Man NP enhanced the activation of CD4^+^ and CD8^+^ T cells, as well as cytotoxic T‐lymphocytes (CTL) (Figure 2F–I and Figure S4A, Supporting Information). Mice treated with Adpgk‐loaded POx‐Man nanovaccine also presented the highest levels of CD8^+^ and CD4^+^ T cells overexpressing the Th1 cytokines interferon (IFN)‐ɣ, interleukin (IL)‐2, and tumor necrosis factor (TNF)‐α (Figure 2J–O and Figure S4A, Supporting Information), which predict an improved cytotoxic CD8^+^/Th1 T‐cell‐mediated systemic activity. This nanovaccine also modulated the Th2 cytokine secretion profile, while inducing lower levels of CD4^+^ T cells expressing IL‐10, when compared to PEG‐Man nanovaccine (Figure 2P and Figure S4A, Supporting Information).

To select the nanovaccine with the strongest anti‐tumor effect against solid tumors and evaluate if the anti‐tumor effect is mediated by CD8^+^ T cells, MC38‐bearing mice were treated with two doses of PLGA‐PEG‐Man or PLGA‐POx‐Man nanovaccines (MHCI‐Adpgk NP/MHCII‐Adpgk NP), 7 days apart, with or without CD8^+^ T‐cell depletion (Figure 2Q). Both nanovaccines co‐delivering Adpgk neoantigens and immune potentiators, despite NP composition, reduced the tumor growth rate when compared to the phosphate buffered saline (PBS)‐treated group, presenting significantly lower average tumor volumes (*p* < 0.05) (Figure 2S and Figure S4B, Supporting Information).

Although being both different from the PBS‐treated group, the strongest tumor growth inhibition with minimal body weight changes (Figure 2R) was observed in mice treated with the POx‐Man nanovaccine (Figure 2S,T) and POx‐Man Nanovaccine + IgG2b isotype control mAb (Figure S4B, Supporting Information), highlighting the added value of the POx‐Man polymer on NP‐mediated anti‐tumor effect. At day 20 following tumor inoculation, mice treated with POx‐Man nanovaccine presented average tumor volumes 2.2‐ and 3‐fold smaller than those treated with the PEG‐Man nanovaccine (*p* = 0.0091) and PBS (*p* < 0.0001), respectively (Figure 2S and Figure S4B, Supporting Information). The tumor volume intragroup variability also decreased for animals treated with the POx‐Man nanovaccine (Figure 2T and Figure S4B, Supporting Information). In addition, the depletion of CD8^+^ T cells dramatically compromised the anti‐tumor effect of PLGA‐POx‐Man nanovaccine, suggesting that CD8^+^ T cells are positively correlated with decreased tumor volume and the therapeutic benefit mediated by POx‐Man nanovaccine (Figure 2S,T and Figure S4B, Supporting Information). Mice treated with POx‐Man Nanovaccine + αCD8 mAb presented average tumor volumes 8.8‐, 6.5‐, and 3‐fold higher than those treated with the POx‐Man nanovaccine (*p* < 0.0001), POx‐Man Nanovaccine + IgG2b isotype control mAb (*p* < 0.0001), and PBS (*p* < 0.0001), respectively (Figure 2S and Figure S4B, Supporting Information). Although also compromised, the anti‐tumor effect of PEG‐Man nanovaccine was shown to be poorly mediated by CD8^+^ T cells (Figure 2S,T and Figure S4B, Supporting Information). An improved cytotoxic CD8^+^/Th1 T‐cell activity can be confirmed by the highest levels of activated CD8^+^ T cells, activated CTL, and the triad IFN‐γ, IL‐2, and TNF‐α induced by Adpgk‐loaded POx‐Man nanovaccine or POx‐Man Nanovaccine + IgG2b isotype control mAb (Figure S4C–K, Supporting Information). This nanovaccine also induced lower levels of IL‐10‐expressing CD4^+^ T and Treg cells when compared to PEG‐Man nanovaccine (Figure S4L,M, Supporting Information). Therefore, we selected the new material‐based POx‐Man nanovaccine for the following immunotherapy combination in vivo studies, in which we evaluated the potential synergistic effect obtained by combining this multi‐functional mannosylated POx‐based nanovaccine with the downregulation of TGF‐β1 on DC and TIME, in addition to TAM and PD‐1 targeting.

### 2.2 Modulation of MC38 Tumor Microenvironment via Co‐Delivery of Peptide Epitopes and Gene Regulators of TGF‐β1 Expression by POx‐Man Nanovaccine and Tumor‐Associated Macrophage Targeting

Since the MC38 TME immune suppression counteracted the long‐lasting effector function of immune cells induced by POx‐Man nanovaccine (Figure 2), and considering solid tumors biology, we hypothesized that the downregulation of TGF‐β1 secretion and TAM modulation within tumor niche would synergize with the nanovaccine, leading to an extensive activation and expansion of effector immune cells that would ultimately lead to the induction of memory lymphocytes.^[^ ^6^, ^7^, ^20^ ^]^

The surface of the single NP platform, mostly composed of PLGA polymer, was modified with POx‐RGD to promote the active targeting mediated by the RGD receptors (α_v_β_3_/α_v_β_5_ integrins) and subsequent accumulation of PLGA‐POx‐RGD NP within TIME.^[^ ^21^ ^]^ These endothelial cell receptors, particularly expressed on neovascular endothelial cells, are upregulated in solid tumors, including CRC, being associated with angiogenesis and therefore with endothelial cell migration and interaction with extracellular matrix.^[^ ^22^ ^]^ Owing to the lower DoL of 3.1% obtained by the sakaguchi assay for POx‐RGD (Figure S1E,G, Supporting Information), when compared to the one obtained for the conjugation of the mannose moiety to POx (Figure S1D,F, Supporting Information), the TIME‐targeted NP were prepared using two percentages (10% and 30% m/m) of POx‐RGD polymer. PLGA‐POx‐RGD (30% m/m) NP were internalized by MC38 cells (*p* < 0.0001) and HMEC1 dermal microvascular endothelial cells (α_v_β_3_/α_v_β_5_^+[^ ^23^ ^]^) (*p* < 0.0001) at a higher extent than non‐targeted NP or NP comprising 10% m/m of RGD‐grafted POx polymer (Figures S5 and S6, Supporting Information). PLGA‐POx‐RGD (30% m/m) NP were therefore selected to deliver combinations of immune potentiators (CpG‐ODN and Poly(I:C)) and siTGF‐β1 to modulate tumor‐infiltrating immune cell sub‐populations and silence the expression of the potent immune suppressor TGF‐β1 cytokine within tumor milieu. The optimal phospate/nitrogen (P/N) (siTGF‐β1:pARG) ratio of 7 was determined by an electrophoretic mobility shift assay (Figure S7, Supporting Information).

The synergistic anti‐tumor effect between the therapeutic nanovaccine and the inhibition of TGF‐β1 secretion and TAM modulation was subsequently evaluated in a preclinical intervention study in MC38‐bearing mice following the schedule in **Figure** **3**A: 1) subcutaneous administration of PLGA‐POx‐Man nanovaccine delivering combinations of MC38 MHCI and MHCII peptides, and TLR ligands (Nanovaccine); 2) peritumoral administration of TIME‐targeted PLGA‐POx‐RGD NP entrapping the TLR ligands and the siTGF‐β1 combined with (i) nanovaccine (Nanovaccine + TIME‐targeted NP), (ii) Nanovaccine co‐entrapping siTGF‐β1(Nanovaccine_siTGF‐β1 + TIME‐targeted NP), and (iii) pexidartinib (Nanovaccine_siTGF‐β1 + TIME‐targeted NP + Pexidartinib).

Figure 3Combined POx‐Man nanovaccine, TAM modulation, and TGF‐β1 secretion inhibition restrict MC38 tumor growth. A) C57BL/6J mice were inoculated subcutaneously with 0.5 × 10^6^ MC38 tumor cells and treated with Adpgk‐loaded POx‐Man nanovaccine, alone or in combination with the immune modulatory therapies siTGF‐β1‐loaded TIME‐targeted NP or the TAM inhibitor pexidartinib, on days 10, 17, and 24. B) Average MC38 tumor growth curves. The data are presented as mean ± s.e.m of MC38‐bearing mice (*n* = 5 animals). Statistical significance was analyzed by one‐way analysis of variance (ANOVA) followed by Tukey multiple comparisons post‐hoc test and *p* values correspond to tumor volume at day 27 after tumor inoculation, relative to Nanovaccine_siTGF‐β1 + TIME‐targeted NP + Pexidartinib group. C–E) Low infiltration of MHCII^−^ CD206^+^ TAM (M2‐like TAM) obtained for the trivalent combination of siTGF‐β1‐loaded POx‐Man nanovaccine with the immune modulatory therapies strongly correlates to restricted tumor growth. Tumor‐infiltrating myeloid subsets for M2‐like TAM (C), MHCII^+^ CD206^−^ TAM (M1‐like TAM) (D), and M1:M2‐like TAM ratio (E). Tumors were recovered on day 27 following tumor inoculation. The quantification was performed by flow cytometry analysis. Data are presented as mean ± s.d., *n* = 3 animals. Statistical significance was calculated by one‐way ANOVA with Tukey multiple comparisons post‐hoc test. F–H) Dysregulation of TGF‐β1 and CSF‐1R expression in CRC after the administration of the combinatorial treatments with the siTGF‐β1‐loaded POx‐Man nanovaccine. F) Quantitative RT‐PCR analysis of *Tgf‐β1* in mice tumors. G,H) Immunoblotting and densitometry of TGF‐β1, phosphorylated CSF‐1R (p‐CSF‐1R), and CSF‐1R. Blots of TGF‐β1 were normalized to endogenous β‐actin, whereas p‐CSF‐1R was normalized to CSF‐1R. Representative immunoblots are shown. Data are presented as mean ± s.d. fold change, *n* ≥ 3 independent samples with two technical replicates. Statistical significance was calculated by one‐way ANOVA with Tukey multiple comparisons post‐hoc test. I) C57BL/6J mice were inoculated subcutaneously with 0.5 × 10^6^ MC38 tumor cells and treated with Adpgk + siTGF‐β1‐loaded POx‐Man nanovaccine (Nanovaccine_siTGF‐β1), alone or in combination with the immune modulatory therapies, siTGF‐β1‐loaded TIME‐targeted NP or pexidartinib, on days 8 and 15. J) Body weight change is expressed as the percent change in weight from the day of treatment initiation. K) Average MC38 tumor growth curves. L) Individual MC38 tumor volumes at day 19 following tumor inoculation. M) Individual tumor growth curves. The data are presented as mean ± s.e.m of MC38‐bearing mice (*n* = 5 animals), replicated in two independent experiments for Nanovaccine, Nanovaccine_siTGF‐β1, pexidartinib, Nanovaccine_siTGF‐β1 + Pexidartinib, Nanovaccine_siTGF‐β1 + TIME‐targeted NP groups, and in three independent experiments for Nanovaccine_siTGF‐β1 + TIME‐targeted NP + Pexidartinib group. Statistical significance was analyzed by one‐way ANOVA followed by Dunnett multiple comparisons post‐hoc test and *p* values correspond to tumor volume at day 19 after tumor inoculation, compared to the Nanovaccine_siTGF‐β1 + Pexidartinib group. N) ELISpot representative images and analysis of IFN‐γ spot forming cells within splenocytes after ex vivo restimulation with relevant Adpgk peptides on day 19.

**Figure:**
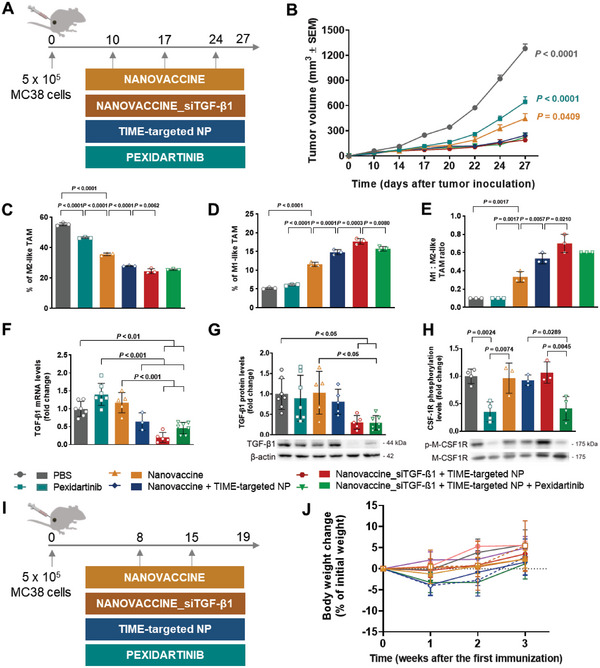

**Figure:**
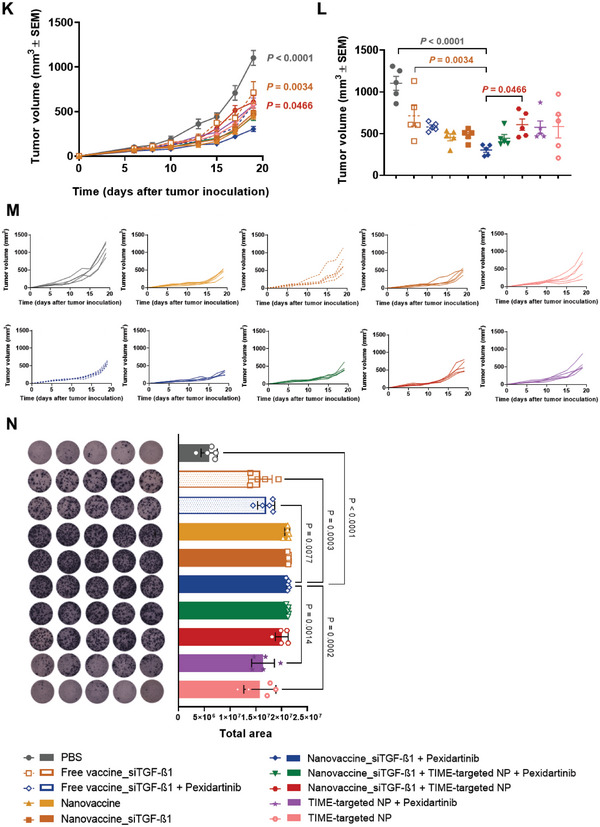

MC38‐bearing mice treated with the combination of Nanovaccine + TIME‐targeted NP presented a significantly lower tumor volume on day 27 (Figure 3B). The combination of these TIME‐targeted NP with nanovaccine co‐entrapping now the siTGF‐β1 in addition to the neoantigen peptides and the TLR ligands (Nanovaccine_siTGF‐β1) resulted in an anti‐tumor effect stronger than the one obtained in the PBS (*p* < 0.0001), pexidartinib (*p* < 0.0001), and nanovaccine (*p* = 0.0409) treatment groups (Figure 3B).

At day 27 following tumor inoculation, the average tumor volume of the combinations Nanovaccine + TIME‐targeted NP, Nanovaccine_siTGF‐β1 + TIME‐targeted NP, and Nanovaccine_siTGF‐β1 + TIME‐targeted NP + Pexidartinib were 2‐, 3‐, and 6‐fold smaller than those obtained for the monotherapies nanovaccine‐, pexidartinib‐, and PBS‐treated mice, respectively (Figure 3B). Tumors collected from mice treated with Nanovaccine + TIME‐targeted NP, Nanovaccine_siTGF‐β1 + TIME‐targeted NP, and Nanovaccine_siTGF‐β1 + TIME‐targeted NP + Pexidartinib presented the lowest infiltration of MHCII^−^ CD206^+^ TAM (M2‐like TAM) (*p* < 0.01) (Figure 3C and Figure S8, Supporting Information), being highly infiltrated by MHCII^+^ CD206^−^ TAM (M1‐like TAM) (Figure 3D,E and Figure S8, Supporting Information).

Despite the similar average tumor volume presented by mice treated with Nanovaccine + TIME‐targeted NP, Nanovaccine_siTGF‐β1 + TIME‐targeted NP, or Nanovaccine_siTGF‐β1 + TIME‐targeted NP + Pexidartinib, the downregulation of TGF‐β1 expression in tumors was only confirmed when the Nanovaccine_siTGF‐β1 was administered subcutaneously (s.c.) in combination with the TIME‐targeted NP, with or without pexidartinib (Figure 3F,G). Both qRT‐PCR and immunoblotting analyses revealed a significant downregulation of the TGF‐β1 expression in the tumor of mice treated with these divalent and trivalent therapies, compared to monotherapies, which was 6‐ and 2.5‐fold (*p* < 0.01) lower for the TGF‐β1 mRNA levels (Figure 3F), 3‐ and 3‐fold (*p* < 0.05) lower for the TGF‐β1 protein levels (Figure 3G), respectively. Importantly, the group treated with nanovaccine (no siTGF‐β1) in combination with the peritumoral administration of TIME‐targeted NP (containing the siTGF‐β1) did not present a significant TGF‐β1 downregulation compared to mice treated with divalent and trivalent therapies including the Nanovaccine_siTGF‐β1 (Figure 3F,G). Therefore, the downregulation of this cytokine in DC and induced modulation of immune infiltrates within tumor mass was crucial for the overall reduction of TGF‐β1 within the tumor milieu. A 3‐ and 2‐fold (*p* < 0.01) downregulation of phosphorylated CSF‐1R protein levels within tumors was also confirmed upon the intraperitoneal (i.p.) administration of pexidartinib, as monotherapy or in the trivalent therapy, respectively (Figure 3H).

### 2.3 Tumor‐Infiltrating Immune Subsets in MC38 Tumors Induced by Nanovaccine_siTGF‐β1 in Combination with Pexidartinib

At day 19 following tumor inoculation (Figure 3I–M), the average tumor volume (Figure 3K,L) of the Nanovaccine_siTGF‐β1 + Pexidartinib group was 1.5‐, 2‐, 2.5‐, and 4‐fold smaller than the ones obtained for mice treated with nanovaccine, Nanovaccine_siTGF‐β1, and Nanovaccine_siTGF‐β1 + TIME‐targeted NP + Pexidartinib; Free vaccine_siTGF‐β1 + Pexidartinib, TIME‐targeted NP, TIME‐targeted NP + Pexidartinib, and Nanovaccine_siTGF‐β1 + TIME‐targeted NP; Free vaccine_siTGF‐β1; and PBS; respectively.

Nanovaccine_siTGF‐β1 + Pexidartinib generated 1.5‐ and 4.5‐fold greater frequencies of Adpgk‐specific CD8^+^ T cells compared with PBS and pexidartinib groups, respectively, for cytotoxic immune responses against MC38 cells (*p* < 0.0001) (Figure S9, Supporting Information). In addition, the IFN‐ɣ enzyme‐linked immune‐spot (ELISpot) assay further confirmed the Adpgk‐specific T‐cell response considering the IFN‐ɣ secretion by splenocytes of mice treated with the Nanovaccine_siTGF‐β1 + Pexidartinib, post‐stimulation with MHCI/MHCII‐Adpgk peptides (Figure 3N).

**Figure 4:**
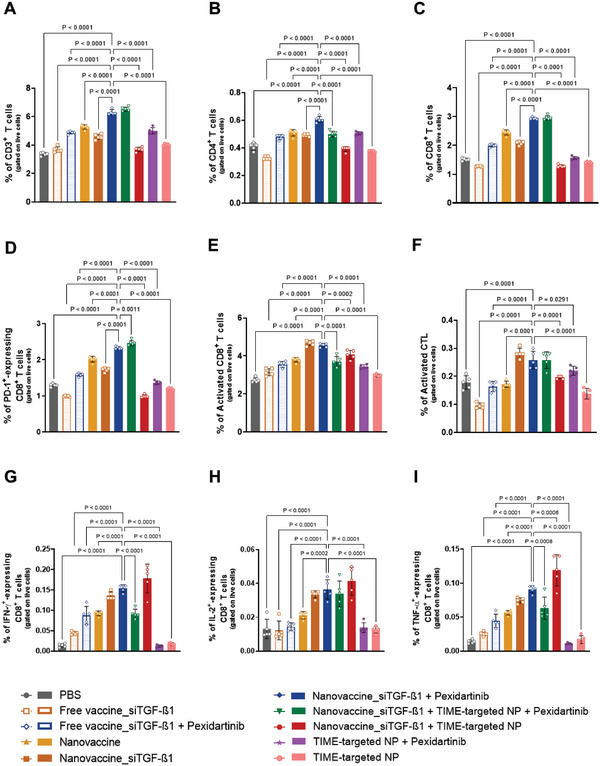
High infiltration of PD‐1^+^‐expressing CD8^+^ T cells may be blocking the full therapeutic potential of the POx‐Man nanovaccine combined with TAM modulation. A–D) Tumor‐infiltrating immune cell populations for CD3^+^ (A), CD4^+^ (B), CD8^+^ (C), and PD‐1‐expressing CD8^+^ (D). Divalent combination of siTGF‐β1‐loaded POx‐Man nanovaccine and TAM modulation triggers a systemic activation of the CD8^+^ T effector. E,F) Percentage of the activated systemic effector CD8^+^ (E) and CTL (F) T cells. Increased systemic secretion of Th1 cytokines (triad IFN‐γ, IL‐2, and TNF‐α)‐expressing CD8^+^ T cells, for siTGF‐β1‐loaded POx‐Man Nanovaccine + Pexidartinib, predicts an improved cytotoxic CD8^+^/Th1 T‐cell activity and correlates with restricted tumor growth. G–I) Secretion of IFN‐γ (G), IL‐2 (H), and TNF‐α (I) by CD8^+^ T cells after restimulation of splenocytes in culture with relevant peptides for 6 h. Tumors and spleens were recovered on day 19 following tumor inoculation. The quantification was performed by flow cytometry analysis. Data are presented as mean ± s.d., *n* = 5 animals. Statistical significance was calculated by one‐way ANOVA with Dunnett multiple comparisons post‐hoc test.

This promising divalent therapy Nanovaccine_siTGF‐β1 + Pexidartinib boosted T‐cell infiltration into tumors (**Figure** **4**A), resulting in the highest levels of tumor‐infiltrating CD4^+^ T cells (Figure 4B), and CD8^+^ T cells (Figure 4C) (*p* < 0.0001) (Figure S10, Supporting Information). However, an increased level of tumor‐infiltrating CD8^+^ T cells expressing PD‐1 was also observed (Figure 4D and Figure S10, Supporting Information) in the tumors of these animals, when compared to PBS‐, Nanovaccine‐, Nanovaccine_siTGF‐β1‐, TIME‐targeted NP‐, and all other divalent combination‐treated groups (*p* < 0.0001).

Increased systemic levels of activated CD8^+^ T cells (Figure 4E and Figure S11D, Supporting Information) and CTL (Figure 4F and Figure S11D, Supporting Information) were also observed for mice treated with Nanovaccine_siTGF‐β1 + Pexidartinib.

High levels of T memory cells have been correlated with a successful relief of disease progression and improved overall and disease‐free survival in CRC patients.^[^ ^24^ ^]^ In this intervention therapeutic study, the highest systemic levels of CD8^+^ T effector memory cells (Figure S11A,D, Supporting Information), which can recirculate through non‐lymphoid tissues and the blood, were obtained for the divalent therapy Nanovaccine_siTGF‐β1 + Pexidartinib (*p* < 0.0001). A significant upregulation of the systemic CD8^+^ T central memory (Figure S11B,D, Supporting Information) and CD8^+^ T naïve memory (Figure S1C,D, Supporting Information) cells was observed for Nanovaccine_siTGF‐β1 + Pexidartinib‐treated mice, when compared to groups treated with PBS (*p* < 0.0001), Free vaccines (*p* < 0.01), or with the divalent and trivalent therapies Nanovaccine_siTGF‐β1 + TIME‐targeted NP (*p* < 0.0001) and Nanovaccine_siTGF‐β1 + TIME‐targeted NP + Pexidartinib NP (*p* < 0.05), respectively.

The highest systemic levels of CD8^+^ T cells overexpressing the Th1 cytokine triad IFN‐ɣ (Figure 4G and Figure S12, Supporting Information), IL‐2 (Figure 4H and Figure S12, Supporting Information), and TNF‐α (Figure 4I and Figure S12, Supporting Information) induced following the treatment of animals with the divalent therapies Nanovaccine_siTGF‐β1 + Pexidartinib and Nanovaccine_siTGF‐β1 + TIME‐targeted NP predicted an improved antigen‐specific cytotoxic T‐cell mediated response.^[^ ^7b^ ^]^ However, this effect correlated more with the stronger tumor growth control observed for mice treated with the combination Nanovaccine_siTGF‐β1 + Pexidartinib.

Moreover, animals treated with this dual therapy showed an enhanced germinal center (GC) response when compared to groups treated with Nanovaccine_siTGF‐β1 + TIME‐targeted NP or Nanovaccine_siTGF‐β1 + TIME‐targeted NP + Pexidartinib, as shown by decreased levels of T follicular regulatory (Tfr) cells (Figure S13C,E, Supporting Information) and increased amount of GC B cells (Figure S13A,E, Supporting Information) and T follicular helper (Tfh) cells (Figure S13B,E, Supporting Information) that may contribute to a stronger immune response through the secretion of antibodies. In fact, only mice treated with nanovaccine, Nanovaccine_siTGF‐β1, and the combination Nanovaccine_siTGF‐β1 + Pexidartinib presented enhanced secretion of IgG antibodies that bound specifically to Adpgk neoantigen peptide (Figure S13D, Supporting Information).

Since an increased level of CD8^+^ T cells overexpressing PD‐1 was observed for both promising divalent Nanovaccine_siTGF‐β1 + Pexidartinib and trivalent Nanovaccine_siTGF‐β1 + TIME‐targeted NP + Pexidartinib combination therapies (Figure 4D), when compared to all other groups (*p* < 0.0001), we hypothesized that the clinical outcomes of our strategy on controlling growth of solid tumors could be further improved by blocking the PD‐1.^[^ ^25^ ^]^

### 2.4 Combination Therapy Comprising Nanovaccine_siTGF‐β1, Pexidartinib, and αPD‐1 Improved the Survival of MC38 and CT26‐Bearing Mice

**Figure 5:**
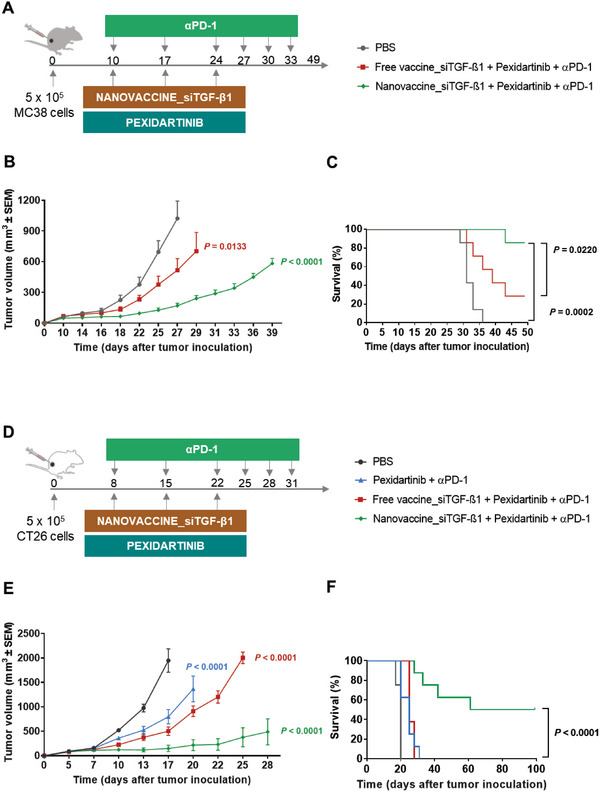
Trivalent combination of POx‐Man Nanovaccine_siTGF‐β1, pexidartinib, and αPD‐1 strongly restricts CRC tumors growth and leads to long‐term survival. A) C57BL/6J mice were inoculated subcutaneously with 0.5 × 10^6^ MC38 tumor cells and treated with POx‐Man Nanovaccine_siTGF‐β1 in combination with both pexidartinib and αPD‐1 (10 mg kg^−1^), on days 10, 17, and 24. On days 3, 6, and 9 after the third treatment (day 24), αPD‐1 (10 mg kg^−1^) was administered intraperitoneally to mice. B) Average MC38 tumor growth curves. Average data are presented as mean ± s.e.m of MC38‐bearing mice (*n* = 7 animals). Statistical significance was analyzed by one‐way analysis of variance (ANOVA) followed by Tukey multiple comparisons post‐hoc test and *p* values correspond to tumor volume at day 27 after tumor inoculation relative to the PBS group. C) Overall survival over time of MC38‐bearing mice (*n* = 7 animals) compared using Kaplan–Meier curves followed by the log‐rank test. D) Balb/c mice were inoculated subcutaneously with 0.5 × 10^6^ CT26 tumor cells and treated with POx‐Man Nanovaccine_siTGF‐β1 in combination with pexidartinib and αPD‐1 (10 mg kg^−1^), on days 8, 15, and 22. On days 3, 6, and 9 after the third treatment (day 22), αPD‐1 (10 mg kg^−1^) was intraperitoneally administered to mice. E) Average CT26 tumor growth curves. Mice treated with POx‐Man Nanovaccine_siTGF‐β1 in combination with both pexidartinib and αPD‐1 showed a robust response, with 4/8 mice showing complete tumor shrinkage. Average data are presented as mean ± s.e.m of CT26‐bearing mice (*n* = 8 animals), replicated in two independent experiments for Nanovaccine_siTGF‐β1 + Pexidartinib + αPD‐1 and Free vaccine_siTGF‐β1 + Pexidartinib + αPD‐1 groups. Statistical significance was analyzed by one‐way analysis of variance (ANOVA) followed by Tukey multiple comparisons post‐hoc test and *p* values correspond to tumor volume at day 17 after tumor inoculation, compared to the PBS group. F) Overall survival over time of CT26‐bearing mice (*n* = 8 animals) compared using Kaplan–Meier curves followed by the log‐rank test.

MC38‐bearing mice received the Nanovaccine_siTGF‐β1, Pexidartininb, and αPD‐1 as shown in **Figure** **5**A. On day 27 following tumor inoculation (last day of the study at which all animals were still alive in all groups), the animals treated with the trivalent therapies (Nanovaccine_siTGF‐β1 + Pexidartinib + αPD‐1 and Free vaccine_siTGF‐β1 + Pexidartinib + αPD‐1) presented average tumor volumes sixfold (*p* < 0.0001) and twofold (*p* = 0.0133) lower than those obtained in the PBS‐treated group (Figure 5B and Figure S14B, Supporting Information). Negligible body weight changes were observed for all treatment groups (Figure S14A, Supporting Information).

Although different from the PBS‐treated group, both trivalent therapies also presented distinct tumor volumes. In fact, on day 29, the group treated with the Nanovaccine_siTGF‐β1 + Pexidartinib + αPD‐1 presented the lowest average tumor volume (241 mm^3^), when compared to animals treated with the Free vaccine_siTGF‐β1 + Pexidartinib + αPD‐1, which presented an average tumor volume of 703 mm^3^ (Figure 5B and Figure S14B, Supporting Information).

On day 39, animals treated with Nanovaccine_siTGF‐β1 + Pexidartinib + αPD‐1 presented a 2.3‐fold higher survival percentage (*p* = 0.0220) compared to animals treated with Free vaccine_siTGF‐β1 + Pexidartinib + αPD‐1 (Figure 5C), of which five out of seven had already reached a tumor volume of at least of 1000 mm^3^ (Figure S14C, Supporting Information). Two out of seven animals (29%) of the group Free vaccine_siTGF‐β1 + Pexidartinib + αPD‐1 remained alive after 49 days, whereas six (86%) out of seven animals treated with Nanovaccine_siTGF‐β1 + Pexidartinib + αPD‐1 survived during that period (Figure 5C). The survival curve of the nano‐based trivalent regimen is statistically different from those obtained for the Free vaccine_siTGF‐β1 + Pexidartinib + αPD‐1 (*p* = 0.0220) and PBS (*p* = 0.0002) treatments.

Importantly, the variability in terms of individual tumor size obtained for animals treated with PBS or the immune modulators in solution was significantly reduced for mice treated with the nano‐based trivalent regimen (Figure 5B and Figure S14B,C, Supporting Information).

Finally, the application of the trivalent therapy Nanovaccine_siTGF‐β1 + Pexidartinib + αPD‐1 for the treatment of solid tumors was further validated in CT26‐bearing mice (Figure 5D), with negligible body weight changes observed for all treatment groups (Figure S15A, Supporting Information).

On day 17 following tumor inoculation (the last day of the study at which all animals were still alive in all groups), the animals treated with Nanovaccine_siTGF‐β1 + Pexidartinib + αPD‐1, Free vaccine_siTGF‐β1 + Pexidartinib + αPD‐1, and Pexidartinib + αPD‐1 showed average tumor volumes 13‐fold (*p* < 0.0001), 4‐fold (*p* < 0.0001), and 2.4‐fold (*p* < 0.0001) lower than those obtained in the PBS‐treated group (Figure 5E and Figure S15B, Supporting Information).

The group treated with the Nanovaccine_siTGF‐β1 + Pexidartinib + αPD‐1 elicited a potent anti‐tumor response (Figure 5E and Figure S15B,C, Supporting Information), inducing complete tumor regression in 50% (four out of the eight) of mice (Figure 5E and Figure S15B,C, Supporting Information), and prolonged overall survival (Figure 5F).

On day 20, the lowest average tumor volume (217 mm^3^) was presented by animals treated with the Nanovaccine_siTGF‐β1 + Pexidartinib + αPD‐1, when compared to those obtained in animals treated with the Free vaccine_siTGF‐β1 + Pexidartinib + αPD‐1 (911 mm^3^) and Pexidartinib + αPD‐1 (1366 mm^3^) (Figure 5E and Figure S15C, Supporting Information).

Nanovaccine_siTGF‐β1 + Pexidartinib + αPD‐1‐treated group presented a 2.7‐ and 4‐fold higher survival percentage (*p* < 0.0001) compared to animals treated with Free vaccine_siTGF‐β1 + Pexidartinib + αPD‐1 and Pexidartinib + αPD‐1 (Figure 5F), respectively, with eight out of eight animals (100%) alive after 25 days, whereas three (37.5%) out of eight animals treated with Free vaccine_siTGF‐β1 + Pexidartinib + αPD‐1, and two (25%) out of eight animals treated with Pexidartinib + αPD‐1 survived during that period (Figure 5F).

Overall, the trivalent combination Nanovaccine_siTGF‐β1 + Pexidartinib + αPD‐1 eliminated the established tumors (≈100 mm^3^ at the initiation of treatment on day 8) in 50% of animals (Figure S15B, Supporting Information), which survived for 99 days (Figure 5F). Free vaccine_siTGF‐β1 + Pexidartinib + αPD‐1 and Pexidartinib + αPD‐1 therapies failed as mice did not survive more than 31 days (Figure 5F).

Animals that responded to the trivalent nano‐immunotherapy (Nanovaccine_siTGF‐β1 + Pexidartinib + αPD‐1) harboring a complete tumor regression, were s.c. challenged at the left flank, on day 33. From those, 100% of mice remained with no disease for more 65 days (day 99), showing its long‐lasting immune memory protection upon rechallenge (Figure S15D, Supporting Information).

### 2.5 Nano‐Based Trivalent Therapy Controlled the Aggressive Tumor Growth of the B16F10 Melanoma Mouse Model

**Figure 6:**
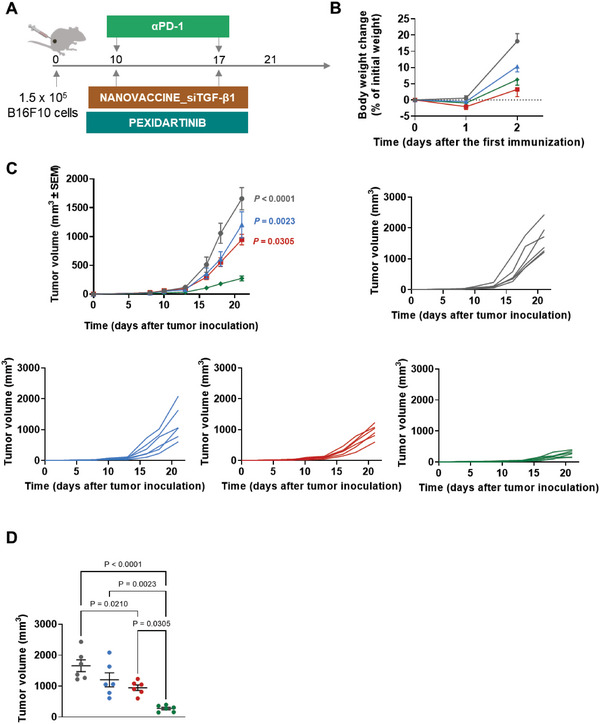
Trivalent combination of POx‐Man Nanovaccine_siTGF‐β1, pexidartinib, and αPD‐1 controlled the aggressive tumor growth of the B16F10 melanoma model. A) C57BL/6J mice were inoculated subcutaneously with 0.15 × 10^6^ B16F10 tumor cells and treated with POx‐Man Nanovaccine_siTGF‐β1 in combination with pexidartinib and αPD‐1 (10 mg kg^−1^), on days 10 and 17. B) Body weight change is expressed as the percent change in weight from the day of treatment initiation. Data are presented as mean ± s.e.m of B16F10‐bearing mice (*n* = *6* animals). C) Average and individual B16F10 tumor growth curves. D) Individual B16F10 tumor volumes at day 21 following tumor inoculation. Average data are presented as mean ± s.e.m of B16F10‐bearing mice (*n* = 6 animals). Statistical significance was analyzed by one‐way analysis of variance (ANOVA) followed by Tukey multiple comparisons post‐hoc test and *p* values correspond to tumor volume at day 21 after tumor inoculation, compared to Nanovaccine_siTGF‐β1 + Pexidartinib + αPD‐1 group.

Motivated by the results previously described, the therapeutic efficacy of the nano‐based trivalent therapy was tested in the aggressive and weakly immunogenic B16F10 melanoma mouse model. Accordingly, B16F10‐bearing mice received the Nanovaccine_siTGF‐β1, pexidartinib, and αPD‐1 according to the schedule in **Figure** **6**A, with negligible body weight changes observed (Figure 6B).

On day 21 following tumor inoculation, the animals treated with Nanovaccine_siTGF‐β1 + Pexidartinib + αPD‐1, Free vaccine_siTGF‐β1 + Pexidartinib + αPD‐1, and Pexidartinib + αPD‐1 showed average tumor volumes 6‐fold (*p* < 0.0001), 1.8‐fold (*p* < 0.0210), and 1.4‐fold (*p* > 0.05) lower than those obtained in the PBS‐treated group (Figure 6C,D).

Although different from the PBS‐treated group, distinct tumor volumes were presented by the divalent and both trivalent therapies. On day 21, Pexidartinib + αPD‐1 (1203 mm^3^) and Free vaccine_siTGF‐β1 + Pexidartinib + αPD‐1 (947 mm^3^) therapies failed to control the tumor growth, presenting the highest average tumor volumes when compared to those obtained in animals treated with the trivalent combination Nanovaccine_siTGF‐β1 + Pexidartinib + αPD‐1 (274 mm^3^) (Figure 6C,D). Importantly, the variability in terms of individual tumor size obtained for animals treated with PBS or the immune modulators in solution was significantly reduced for mice treated with the nano‐based trivalent regimen (Figure 6C,D).

This superior anti‐tumor effect was also supported by the tolerability and safety of the nano‐based trivalent regimen, demonstrated by the absence of acute toxicity signs (Figure S16A–C, Supporting Information). On day 21 following tumor inoculation, the biochemical analysis of murine blood showed increased levels for the activity of the aspartate aminotransferase (AST) (Figure S16D, Supporting Information), alanine aminotransferase (ALT) (Figure S16E, Supporting Information), and gama glutamil transferase (GGT) (Figure S16F, Supporting Information) for PBS‐treated group, induced by the aggressive disease model‐associated toxicity. In contrast to Pexidartinib + αPD‐1 and Free vaccine_siTGF‐β1 + Pexidartinib + αPD‐1 treatments, the benefit of the nano‐based trivalent regimen was also endorsed by the basal levels of the liver function enzymes (Figure S16D–F, Supporting Information). No alterations were observed for urea and creatinine levels (Figure S16G,H, Supporting Information) in response to all treatments. Histological differences were also observed among free and nano‐based trivalent regimens (Figure S16I, Supporting Information). Multifocal foci of inflammatory cell infiltration (mononuclear) associated with moderate liver necrosis were observed in the liver of animals treated with the Free vaccine_siTGF‐β1 + Pexidartinib + αPD‐1 (Figure S16I, Supporting Information). No significant alterations (within normal limits) were detected in the heart, kidney, and spleen (Figure S16I, Supporting Information). Overall, this study validated our hypothesis by confirming the synergism observed between pexidartinib, αPD‐1, and our multivalent POx‐based nanovaccine, which translated into a strong tumor growth control of the poorly immunogenic B16F10 melanoma model. These data overall support the potential application of our trivalent approach as an efficient and safe immunotherapy against solid tumors.

## 3 Discussion

The clinical approval of targeted therapeutic regimens, alone or with a variety of combinations as the first, second, and third line of treatments, has revitalized the management of advanced solid tumors. However, alternative multi‐targeted combinatorial schemes designed to interfere with immune modulatory or immune suppressive mechanisms, which dictate tumor cells’ differentiation, proliferation, and dissemination, are urgent to achieve durable therapeutic efficacy, while overcoming the intensity and frequency of serious adverse effects.

Cancer vaccination emerged as an interesting tool to synergize and improve the outcome of other therapeutic approaches (e.g., immune modulators), due to its ability to induce tumor‐specific CTL responses against cancer antigens by increasing their recognition, processing, and presentation to effector T cells. Anti‐tumor therapeutic vaccines may be a key player in favorably shifting the equilibrium between an immune suppressive pro‐tumoral environment and long‐term anti‐tumoral immunity, potentiating ongoing surveillance and thereby overcoming therapy resistance, metastasis, and tumor recurrence.

Herein, we report the development of a nanovaccine, in which the amphiphilic polymer POx was explored as an alternative to PEG.^[^ ^3^, ^26^ ^]^ Although PEG is a polymer widely used to improve the half‐time of carriers by avoiding their premature capture by macrophages, POx are emerging as a class of biocompatible polymers alternative to PEG by presenting high synthetic versatility and structural modularity,^[^ ^27^ ^]^ in addition to overcoming the immunogenic issues that recently emerged against PEGylated‐based therapies concerning the development of anti‐PEG antibodies.^[^ ^2^, ^28^ ^]^ Adverse effects, including the ones resulting from the off‐target accumulation of nanocarriers, have not been reported for POx derivates, which were described as having a rapid renal clearance and excretion.^[^ ^29^ ^]^ A significantly improved stability for POx was demonstrated due to the longer retention of anti‐fouling properties of POx‐modified surfaces compared to PEG, under physiological and oxidative conditions.^[^ ^3^, ^30^ ^]^ POx coatings have been reported to have a very low plasma protein adhesion, in addition to the ability to delay NP recognition in vitro by mononuclear phagocyte cells, and macrophages, at a higher extent when compared to traditional PEG coatings.^[^ ^3^, ^26^ ^]^

To potentiate more effectively and selectively the interaction with the mannose receptor (CD206) and enhance the payload delivery to DC,^[^ ^14^ ^]^ our nanovaccines were functionalized with mannose. The obtained nanovaccine mean average diameter close to 200 nm is suitable to travel through the lymph drainage reaching the lymphoid organs within 2–3 h after administration.^[^ ^31^ ^]^ These nanovaccines can also be recognized and internalized at the site of injection by immature DC, which subsequently traffic to LN within 18 h.^[^ ^31^, ^32^ ^]^ PLGA‐POx‐Man led to a stable nanovaccine formulation, which presented improved internalization levels in vivo by APC of the myeloid compartment, compared to the PEGylated formulation.^[^ ^33^ ^]^ These results suggest that the mannose moieties decorating the surface of the PLGA‐POx‐Man NP presented a favorable interaction with the mannose receptors when compared to those available at the surface of the PLGA‐PEG‐Man NP. This may be due to a decrease in the interfacial tension between the PLGA‐based NP surface and the surrounding aqueous environment, caused by the POx coating, as previously shown by Tryba et al.,^[^ ^33^ ^]^ allowing for active targeting and a more extensive internalization by APC.

The preferential accumulation of nanovaccines in peripheral LN is extremely important for vaccination since these lymphoid organs represent the site where APC, especially DC, communicate with naïve T cells to induce antigen‐specific adaptive immune responses.^[^ ^34^ ^]^ However, only mature DC can potently activate naïve T cells through the extension on dendrites (one DC can activate 500 different naïve T cells in 1 h),^[^ ^35^ ^]^ allowing their expansion and differentiation into effector and memory cells in a cytokine‐dependent manner.^[^ ^36^ ^]^ Apart from the downregulation of DC endocytic activity, acquisition of motility to draining LN and naïve T cell stimulation by the antigen‐MHC complex presented by DC,^[^ ^37^ ^]^ secondary stimuli involving the upregulation of co‐stimulatory molecules, such as CD40, CD80, and CD86, on DC surface that interact with the CD40 ligand and CD28 receptor on naïve T cells, respectively, is required for the activation and clonal expansion of naïve T cells.^[^ ^38^ ^]^ The overexpression of the co‐stimulatory/maturation markers CD80/86 on the surface of activated circulating DC was significantly induced by Adpgk‐loaded POx‐Man NP. This outcome was expected, as the co‐delivery of tumor antigens and TLR ligands by a nanocarrier was previously shown to enhance antigen internalization, processing, and subsequent presentation, which is a key step to overcome host tolerance to tumor cells by improving effective T‐cell priming and lymphocyte expansion.^[^ ^17^, ^39^ ^]^ Compared to PEG‐Man nanovaccine, the co‐delivery of antigens and adjuvants by POx‐Man nanovaccine was expected to induce a cytotoxic CD8^+^/Th1 T‐cell response predicted by the enhanced activation of CD4^+^ and CD8^+^/CTL and by the highest levels of CD8^+^ and CD4^+^ T cells overexpressing the Th1 cytokines.

Despite NP composition, both PEG‐Man and POx‐Man nanovaccines, co‐delivering Adpgk neoantigens and immune potentiators, reduced the tumor growth rate when compared to the PBS‐treated group. A stronger antigen‐specific CTL immune response capable of suppressing CRC growth and improving animal survival was previously reported in MC38‐ and CT26‐bearing mice when antigen and adjuvants were delivered by adjuvant particulate nanovaccines, in contrast to soluble molecules and other controls.^[^ ^39b–f^ ^]^ Nanovaccines co‐entrapping both CpG‐ODN and Poly(I:C) allowed the multi‐targeting synergistic co‐stimulatory effect due to the simultaneous engagement of both TLR9 and TLR3, respectively, at the endosomal compartment. Previous studies have coined CpG and Poly(I:C) as important players in the induction of robust tumor‐specific T‐cell responses potentiated by APC activation and maturation when combined with vaccine formulations.^[^ ^40^ ^]^ In addition, it was recently reported the synergistic activity of the combination of these two TLR ligands, which led to stronger antigen‐specific T helper 1‐biased immunity against tumors,^[^ ^41^ ^]^ essential to control tumor homeostasis, especially concerning inflammatory and angiogenic events.^[^ ^42^ ^]^ Interestingly, POx nanovaccine controlled tumor growth in MC38‐bearing mice at a higher extent than the PLGA‐PEG‐Man formulation in a CD8^+^ T cell‐dependent mechanism, showing its promising application for the targeted co‐delivery of MC38 antigens and TLR ligands to DC, and subsequent anti‐tumor cytotoxic immune response. We also compared the outcome of our PLGA‐POx‐Man nanovaccine with some others already reported in the literature against solid tumors. The polymeric‐based‐nanovaccine code‐named PC7A NP comprises a cocktail of three tumor neoantigens (Reps1_P45A_, Adpgk_R304M_, Dpagt1_V213L_) into PC7A NP that were efficiently delivered to DC at draining LN inducing strong cytotoxic T‐cell responses.^[^ ^39b–f^ ^]^ However, our PLGA‐POx‐Man nanovaccine has shown improved MC38 growth inhibition at day 24 post‐tumor inoculation. In addition, Ni and co‐workers also reported the therapeutic efficacy of banNVs, which were formulated by encapsulating Adpgk into CpG/R848 NP.^[^ ^1a^ ^]^ However, these banNVs did not present an improved therapeutic efficacy over our PLGA‐POx‐Man NP at day 27 post‐tumor inoculation. Accordingly, the potential synergistic anti‐tumor effect of the new material‐based POx‐Man nanovaccine combined with modulatory therapies focused on blocking immune suppressive mechanisms involved in cancer progression was further explored.

Despite the potential role of nanovaccines in re‐educating host immunity against cancer cells, multiple processes and subsets of cells contribute to the pathogenesis of this complex process. The upregulation of the TGF‐β cytokine has been associated with metastasis and related poor prognosis in advanced cancer patients.^[^ ^7^ ^]^ TGF‐β1 signaling was also reported to play a pivotal role in the modulation of T‐cell development and in the promotion of regulatory functions and immunological tolerance in DC, crucial to initiate potent adaptive immune responses.^[^ ^43^ ^]^ The inhibition of this cytokine led to potent cytotoxic immune responses and prevention of metastatic solid tumors such as mCRC^[^ ^20b,c^ ^]^ and melanoma metastases.^[^ ^44^ ^]^ All these findings stimulated the development of strategies to inhibit the TGF‐β pathway, either as monotherapy or in combination with other therapies, to restore anti‐cancer immunity.^[^ ^45^ ^]^

TAM infiltration also plays an important role in melanoma and CRC progression, being correlated with poor clinical outcomes. Particularly, M2‐like TAM act as stimulators of Treg differentiation and tumor progression through the upregulation of immune suppressive cytokines such as TGF‐β and IL‐10.^[^ ^46^ ^]^ TGF‐β/IL‐10 signaling and secretion by Treg and myeloid‐derived suppressor cells are highly correlated with regulatory and immune suppressive functions, such as the inhibition of co‐stimulatory molecules, suppression of DC maturation, generation of regulatory DC, differentiation and expansion of Treg, and consequent prevention of effector T‐cell activation and proliferation.^[^ ^47^ ^]^ In addition, M2‐like TAM promote Treg accumulation within TIME, which is also associated with faster angiogenesis.^[^ ^46^, ^47^ ^]^ The modulation of CSF‐1/CSF‐1R signaling involved in the control of macrophage differentiation, function, and survival, has been reported to repolarize adaptive immune cells (converting them to anti‐tumor cells) by reducing TAM infiltration and promoting effector CD8^+^ T cells in CRC^[^ ^6a–g^ ^]^ and other anti‐tumor subsets.^[^ ^6^, ^48^ ^]^ Therefore, the tumor‐permissive and immune suppressive characteristics of TAM have fueled interest in therapeutically targeting these cells using CSF‐1R inhibitors (e.g., pexidartinib), as monotherapy or in combination with other immunotherapeutic strategies, including DC vaccination and checkpoint inhibition. The safety of pexidartinib alone (phase 1 dose escalation) or in combination with αPD‐L1 (Durvalumab) (recommended phase 2 dose), and the clinical activity of this combination (extension part) was recently reported in patients with advanced/mCRC and pancreatic cancer.^[^ ^49^ ^]^

Accordingly, we report the combined delivery of antigens, TLR ligands CpG and Poly(I:C), and siTGF‐β1, a DC immunosuppressive player, by a single nanoparticulate system aiming at the activation and maturation of DC. The delivery of the siTGF‐β1 within POx nanovaccine downregulated TGF‐β1 expression within the TME, which was not observed upon administration of the nanovaccine without siRNA or the peritumoral injection of TIME‐targeted NP. The POx‐based nanovaccine potentiated tumor‐specific T‐cell responses that correlated with delayed tumor growth when combined with pexidartinib to modulate M2‐like TAM. This dual therapy Nanovaccine_siTGF‐β1 + Pexidartinib overcame the need for the peritumoral administration of the TIME‐targeted NP. However, an increased level of tumor‐infiltrating CD8^+^ T cells expressing PD‐1 was also found in mice treated with this dual nano‐immunotherapy.

Previous studies have shown that the simultaneous administration of cancer nanovaccines and αPD‐1 further promoted the anti‐tumor efficacy and prolonged MC38‐bearing mouse survival when compared to αPD‐1 alone or combined antigens in the solution.^[^ ^1a^ ^]^ The combination Nanovaccine_siTGF‐β1 + Pexidartinib + αPD‐1 indeed strongly controlled the tumor growth and prolonged the survival of MC38‐, CT26‐, and B16F10‐bearing mice, in contrast to the association of pexidartinib and αPD‐1 with the delivery of antigens and adjuvants in solution. These outcomes demonstrate the potential use of our nanoplatform as a general nanotechnology‐based strategy for cancer immunotherapy by synergizing with inhibitors of pro‐tumor TAM function and TGF‐β1 secretion to turn a highly immunosuppressive milieu into an immunoreactive TME, thereby overcoming immune tolerance.

## 4 Experimental Section

### Materials and Reagents

Poly(*D,L*‐lactide‐*co*‐glycolide) (PLGA) Resomer RG 502 with a molecular weight (*M*_w_) range 7000–17 000 was purchased from Evonik. Poly(ethylene glycol) methyl ether‐block‐poly(lactide‐*co*‐glycolide) (PLGA‐PEG) (lactide:glycolide 50:50, PEG average *M*_w_ 2000, PLGA average *M*_w_ 11 500), PLGA Resomer RG 503H with a *M*_w_ range 24 000–38 000, poly(vinyl alcohol) (PVA, *M*_w_ 13 000–23 000 Da), dichloromethane (DCM), (deuterated) dimethyl sulfoxide (DMSO or dDMSO), dimethylformamide (DMF), 4‐dimethylaminopyridine (DMAP), *N*,*N*′‐dicyclohexylcarbodiimide, methanol, anhydrous sodium sulfate, hexane, *N,N*′‐disuccinimidyl‐carbamate (DSC), DNS, (deuterated) chloroform (CHCl_3_ or CDCl_3_), trifluoroacetic acid (TFA), toluene, diethyl ether, *N*‐hydroxysuccinimide (NHS), acetonitrile (ACN), *N,N*′‐diisopropylethylamine (DIPEA), methyl trifluoromethanesulfonate (methyltriflate), chlorobenzene, 2‐butyl‐2‐oxazoline, and 2‐methyl‐2‐oxazoline (MeOx) monomers, *N*‐boc‐piperazine, potassium carbonate, triisobutylsilane (TIBS), ethidium bromide solution, *D*‐mannosamine hydrochloride (Man), fluorescamine, (3‐aminopropyl)triethoxysilane, tetramethylbenzidine (TMB) ultra‐sensitive blue, formaldehyde solution 4% buffered pH 6.9 for histology, horseradish peroxidase substrate, bovine serum albumin (BSA), and monoclonal anti‐β‐Actin antibody (A5541) were purchased from Sigma‐Aldrich. Boc‐PEG‐amine (PEG, *M*_w_ 3000 Da) was purchased from IRIS Biotech GmbH. Cyanine5 (Cy5)‐carboxylic acid was purchased from Lumiprobe GmbH. Cy5‐grafted PLGA (PLGA‐Cy5) was synthesized by esterification based on Freichels et al.^[^ ^50^ ^]^ *N*‐butyl poly‐L‐arginine hydrochloride (pARG, *M*_w_ range 3000–3400) was purchased from Polypeptide Therapeutic Solutions. Bis(sulfosuccinimidy)suberate (BS^3^), 1‐ethyl‐3‐(3‐dimethylaminopropyl)carbodiimide hydrochloride (EDAC), PBS (pH 7.4), Quant‐iT PicoGreen dsDNA assay kit, Quant‐iT RNA Assay Kit (broad range), Quant‐iT OliGreen ssDNA assay kit, minimum essential medium (MEM)‐α (nucleosides, no ascorbic acid), Roswell Park Memorial Institute (RPMI) 1640 + Glutamax, MCDB 131 medium (no glutamine), HEPES buffer (1 m), heat‐inactivated fetal bovine serum (FBS), penicillin/streptomycin (PEST; Penicillin 10 000 unit mL^−1^ and Streptomycin 10 000 unit mL^−1^), sodium pyruvate (100 mm), L‐glutamine (200 mm), MEM non‐essential amino acids 100×, β‐mercaptoethanol (50 mm), trypsin‐ethylenediaminetetraacetic acid (trypsin‐EDTA, 0.25%), propidium iodide, LIVE/DEAD fixable yellow dead cell stain kit (for 405 nm excitation), ACK lysing buffer, CD28 mAb (37.51), eBioscience Brefeldin A Solution (1000X), eBioscience, Hoechst 332, wheat germ agglutinin Alexa Fluor 488, halt protease and phosphatase inhibitor cocktail were purchased from Thermo Fisher Scientific. Acrylamide/bisacrilamide, marker for sodium dodecyl sulfate‐polyacrylamide gel electrophoresis (SDS‐PAGE; Precision Plus Protein standards, all blue, *M*_w_ 10 000–250 000), Bio‐Rad protein assay kit, and secondary antibody conjugated to horseradish peroxidase were purchased from Bio‐Rad. Agarose, tris‐acetate‐EDTA (TAE) 50× buffer, loading buffer, paraformaldehyde (PFA) 16% m/v methanol free aqueous solution, and TRIzol reagent were purchased from VWR Scientific. Recombinant murine granulocyte macrophage‐colony stimulating factor (GM‐CSF) was purchased from PeproTech. Tumor‐associated peptides Adpgk_R304M_ MHC class I‐restricted peptide (MHCI‐Adpgk): ASMTN[R/M]ELM (AM‐9), Adpgk_R304M_ MHCII‐restricted peptide (MHCII‐Adpgk): GIPVHLELASMTN[R/M]ELMSSIVHQQVFPT (GT‐28), KRAS_G12D_ MHCI‐restricted peptide (MHCI‐KRAS_G12D_): VVGA[G/D]GVGK, KRAS_G12D_ MHCII‐restricted peptide (MHCII‐KRAS_G12D_): KLVVVGA[G/D]GVGKSALTI, and MUT30 MHCII‐restricted peptide (MHCII‐MUT30): PSKPSFQEFVDWE[K/N]VSPELNSTDQPFL were purchased from GeneCust. CpG‐ODN 1826 (TCCATGACGTTCCTGACGTT) and siRNA anti‐TGF‐β1 (siTGF‐β1) were purchased from Microsynth GmbH. Poly(I:C) (High *M*_w_) VacciGrade was purchased from InvivoGen. Pexidartinib was purchased from Selleckchem. In vivo monoclonal antibodies anti‐mouse PD‐1 (RMP1‐14) and CD8α (2.43), and rat IgG2b isotype control – anti‐keyhole limpet hemocyanin (LTF‐2) were purchased from BioXCell. H‐2D^b^‐restricted Adpgk_R304M_ (ASMTN[R/M]ELM) PE‐labeled tetramer was kindly provided by National Institutes of Health (NIH, USA). Corning Matrigel growth factor‐reduced basement membrane matrix, phenol red‐free, was supplied by Corning. Fluorochrome‐labeled antibodies and Inside Stain kit were purchased from Miltenyi Biotec and BioLegend. Collagenase type II, neutral protease (dispase) and DNase I were purchased from Worthington Biochemical Corporation. Rabbit polyclonal antibody against TGF‐β1 (ab92486) was purchased from Abcam. Rabbit monoclonal antibodies against macrophage colony‐stimulating factor 1 receptor (M‐CSF1R) and phospho‐M‐CSF1R (p‐M‐CSF1R, Tyr723, 49C10) were purchased from Cell Signaling Technology, Inc. ELISpot kit was purchased from R&D Systems Inc. Peroxidase AffiniPure Goat Anti‐Mouse IgG was purchased from Jackson Immuno Research Laboratories.

### Synthesis and Characterization of Mannose‐Grafted PLGA‐PEG/POx and RGD‐Grafted POx Polymers

Mannose‐grafted PLGA‐PEG polymer (PLGA‐PEG‐Man) was synthesized through standard amine‐coupling reactions using carbodiimide and NHS‐mediated chemistry from the synthesis of Boc‐PEG‐mannosamine and amine‐PEG‐mannosamine. Briefly, mannosamine (4.3 mg, 0.020 mmol, 4 eq.) was added to the reaction mixture between the BS^3^ (2.9 mg, 0.005 mmol, 1 eq.) and Boc‐PEG‐amine (100 mg, 0.033 mmol, 6.6 eq.) previously dissolved in 10 mm borate buffer pH 8.2 and let under magnetic stirring for 4 h at 40 °C. The resulting Boc‐PEG‐mannosamine was then dissolved in 10% v/v TFA in anhydrous DCM and allowed to stir for 2 h at room temperature. DCM was then removed by rotary evaporation and the crude mixture was purified by co‐evaporation with toluene/methanol/diethyl ether. The resulting amine‐PEG‐mannosamine compound was dried under a vacuum. Finally, NHS (5.3 mg, 0.046 mmol, 7 eq.) was added to the reaction mixture between the PLGA Resomer 503H (200 mg, 0.0065 mmol, 1 eq.) previously dissolved in dry DCM and EDAC (8.8 mg, 0.046 mmol, 7 eq.), and let under magnetic stirring for 1 h at room temperature. The resulting PLGA‐NHS product was precipitated with ice‐cold methanol (20 mL), recovered by centrifugation, and dried under a vacuum. The reaction among amine‐PEG‐mannosamine (20 mg, 0.0067 mmol, 1 eq.) and DMAP (7.3 mg, 0.060 mmol, 9.2 eq.) added to PLGA‐NHS (200 mg, 0.0065 mmol, 1 eq.) previously dissolved in dry DCM was allowed to stir for 18 h at room temperature. The resulting crude was then precipitated in ice‐cold mixture methanol:diethyl ether (7:3) and recovered by centrifugation. After being washed twice, the PLGA‐PEG‐mannose was isolated and dried under a vacuum. ^1^H‐NMR spectra were acquired using a Bruker Avance NMR spectrometer at 300 MHz using dDMSO as solvent (Figure S1C, Supporting Information). Chemical shift data was obtained as δH in ppm and referenced against the deuterated solvent used. PLGA‐PEG‐Man ^1^H‐NMR spectra were compared with the individual ^1^H‐NMR spectra of mannosamine, PLGA, and Boc‐PEG‐amine.

Poly(2‐butyl‐2‐oxazoline)‐*block*‐poly(2‐methyl‐2‐oxazoline) (POx) alone and conjugated to mannosamine (POx‐Man) or to the tripeptide motif RGD (POx‐RGD) were synthesized. The synthesis of POx‐Man and POx‐RGD polymers was carried out under inert conditions through the activation of the POx polymer with DSC, before the functionalization with mannosamine and RGD. POx polymer was primarily synthesized (Figure S1A,B, Supporting Information). Briefly, dried methyltriflate (0.37 g, 2.25 mmol, 1 eq.) was used as the initiator, and the first monomer 2‐butyl‐2‐oxazoline (BuOx) (2.86 g, 22.53 mmol, 10 eq.) were added to dried ACN and dried chlorobenzene. The polymerization of the first block was carried out for 3 h at 110 °C. The second monomer MeOx (6.7 g, 78.8 mmol, 35 eq.) was then added to the previous reaction mixture and the second block was allowed to polymerize for 4 h at 110 °C. Termination with *N*‐boc‐piperazine (2.1 g, 11.3 mmol, 5 eq.) occurred overnight at 40 °C. Afterward, the reaction mixture was neutralized with potassium carbonate overnight and the potassium carbonate residues were removed by centrifugation. The solvent was allowed to evaporate under vacuum and POx polymer dissolved in methanol and then precipitated in 20‐fold excess of ice‐cold diethyl ether. Ether and the precipitated polymer were separated by centrifugation and the polymer dried under vacuum. POx polymer was solubilized in water and lyophilized. The POx bears a non‐reactive methyl group (2.98 and 2.85 ppm, peak 1) (Figure S1A, Supporting Information). The signals of the polymer backbone 2 could be found in a broad peak (3.6–3.3 ppm) and those of the side‐chain 3 at 2.01 ppm (Figure S1A, Supporting Information). Degree of polymerization could be calculated from the integral intensity ratios of methyl end‐group protons and polymer backbone protons.^[^ ^51^ ^]^ The ratio of peaks 2 and 1 gives a degree of polymerization of 56 (Figure S1A, Supporting Information). The amount of 2‐butyl‐2‐oxazoline groups and thus the copolymer composition could be calculated from peaks 4 or 5 and 1, respectively, which gives a degree of polymerization of 11–12 (Figure S1A, Supporting Information). This structure was confirmed by matrix‐assisted laser‐desorption ionization coupled to time‐of‐flight (MALDI‐TOF) mass spectrometry (Figure S1B, Supporting Information). The distribution showed Δ*m*/*z* values that correspond to the molar masses of the monomers and display the molar masses that fit to the desired polymer structure (ionized by a proton). Previously to the POx‐Man and POx‐RGD polymer synthesis, the POx polymer was deprotected being solubilized in the deprotection solution (95% v/v TFA and 2.5% v/v TIBS in water) for 30 min, at room temperature. After stopping the reaction by adding a threefold excess of methanol and removing the solvent under a vacuum, the polymer was dissolved in water and lyophilized. Deprotected POx polymer (4.19 g mL^−1^, 1.067 mmol, 1 eq.) was then solubilized in DMF and added dropwise to an ice‐cold solution of DSC (0.82 g mL^−1^, 3.202 mmol, 3 eq.) in DMF (extra dry). Since DSC was not stable in water, DMF was selected as the reaction solvent. After the addition of DIPEA (0.365 g mL^−1^, 2.134 mmol, 2 eq.) as a catalyst agent, the mixture was allowed to stir for 1 h at 0 °C, following 3 days at room temperature, to activate the carbamate at the piperazine end group of the polymer. Afterward, the solvent was removed under vacuum, the residue was dissolved in methanol/chloroform (2:1) and the activated polymer precipitated twice in a 15‐fold excess of ice‐cold diethyl ether. After separating the polymer and ether by centrifugation, the polymer was dissolved in water and lastly lyophilized. After the DSC activation reaction, mannosamine (0.203 g mL^−1^, 0.937 mmol, 2 eq.) or RGD (30 g mL^−1^, 0.347 mmol, 1.2 eq.) dissolved in DMF (extra dry) were added to the activated POx polymer (2.22 or 1.37 g mL^−1^, 0.468 or 0.289 mmol, 1 eq. for mannosamine or RGD, respectively) also dissolved in DMF (extra dry). The mixture was cooled to 0 °C and subsequently, the DIPEA (0.319 or 0.1 g mL^−1^, 1.875 or 0.577 mmol, 4 or 2 eq. for mannosamine or RGD, respectively) was added. The reaction was allowed to stir for 30 min at 0 °C and then for 3–5 days at room temperature. Finally, the polymers were dried under a vacuum, solubilized in water, and lyophilized. Since the mannose end group does not provide evident peaks, being undetectable in the ^1^H‐NMR spectra of the mannose‐grafted POx polymer, the reaction was confirmed, and the DoL was determined by DNS assay (Figure S1D, Supporting Information). Similarly, POx and RGD signals overlapped in the ^1^H‐NMR spectra. The signal between 2.20 and 2.33 ppm results from the methylene group 3 of the POx polymer side chain from the BuOx block and the RGD end group (Figure S1E, Supporting Information). To confirm the reaction and to determine the DoL of POx‐RGD the sakaguchi assay was performed. The structures of POx‐Man and POx‐RGD (Figures S1F and S1G, Supporting Information, respectively) were confirmed by MALDI‐TOF mass spectrometry.

### Electrophoretic Mobility Shift Assay

siTGF‐β1 (50 pmol total) was mixed with increasing amounts of pARG (1:20, 1:10, 1:7, and 1:5 P/N ratios) in RNase‐free water, incubated under a slow stirring, for 1 h at room temperature. The optimal P/N ratio for polyplex formation and retardation of siRNA mobility was analyzed by electrophoresis on a 2% m/v agarose gel, for 30 min at 100 V in TAE 1× buffer.

### Synthesis of Polymeric Multifunctional NP

PLGA‐based NP were prepared by a double emulsion (water‐in‐oil‐in‐water [w/o/w]) solvent evaporation method, as reported elsewhere with modifications.^[^ ^52^ ^]^ Briefly, polymeric blends (Table S2, Supporting Information) were dissolved in DCM at 50 mg mL^−1^. The TLR ligands (CpG‐ODN at 0.1 mg mL^−1^ and Poly(I:C) at 0.2 mg mL^−1^) and the neoantigens (MHCI‐Adpgk/KRAS_G12D_ and MHCII‐Adpgk/KRAS_G12D_/MUT30 at 5 mg mL^−1^) were dissolved in 8% m/v PVA, to which the polyplex pARG‐siTGF‐β1 at 0.2 mg mL^−1^ (100 µL) was subsequently added. This aqueous internal phase was then added to the organic phase containing the polymer blends dissolved in DCM. The internal aqueous phase used for the synthesis of empty NP contained the pARG dissolved in the 8% m/v PVA. The mixture was emulsified under continuous sonication at 20% of amplitude for 15 s, using a microprobe ultrasonic processor. A second emulsion was performed by adding the 2.5% m/v PVA aqueous solution (400 µL) to that w/o emulsion under the same conditions. The resultant w/o/w double emulsion was subsequently added dropwise into a 0.25% m/v PVA aqueous solution and stirred for 1 h at room temperature. NP were separated by centrifugation at 22 000 × *g* for 40 min, at 4 °C, and resuspended in PBS or ultrapure water. Cy5‐labeled NP were prepared by adding 2.5 (in vitro) or 18.75 (in vivo) mg mL^−1^ of Cy5‐grafted PLGA to the polymer blend.

### Size Distribution and ζ Potential Measurements

A Zetasizer Nano ZS equipment (Malvern Instruments) was used to determine the NP mean diameter and PdI by DLS. The same equipment allowed for the determination of NP surface charge (ζ potential) by laser Doppler electrophoresis, in combination with phase analysis light scattering. NP (0.5 mg mL^−1^) were diluted in PBS or ultrapure water, and their Brownian motion based on laser light scattering (NP size) and electrophoretic mobility using the Helmholtz‐von Smoluchowski model (ζ Potential) were determined at 25 °C by cumulative analysis.

### Particle Morphology

NP surface morphology was characterized by AFM, using a Nanoscope IIIa Multimode AFM (Digital Instruments, Veeco). Samples were prepared by depositing a drop of final colloidal suspension (10 mg mL^−1^) onto freshly cleaved mica for 15 min at room temperature and dried with pure nitrogen. Samples were analyzed in tapping mode in air at room temperature using etched silicon tips (≈300 kHz), at a scan rate of ≈1.6 Hz.

### EE and LC of Neoantigens, Immune Modulators, and Gene Regulator

The amount of neoantigens (MHCI‐Adpgk/KRAS_G12D_ and MHCII‐Adpgk/KRAS_G12D_/MUT30), gene regulator (siTGF‐β1), and immune potentiators (CpG‐ODN and Poly(I:C)) entrapped in the NP were indirectly quantified in supernatants collected from centrifugation steps following the preparation of NP. EE (Equation (1)) and LC (Equation (2)) of neoantigens (MHCI‐Adpgk/KRAS_G12D_ and MHCII‐Adpgk/KRAS_G12D_) were quantified using fluorescamine. Fluorescence intensity was measured at 360/460 nm for the absorbance/emission wavelengths, using a FLUOstar Omega plate reader (BMG Labtech). MHCII‐Adpgk/MUT30 peptides were quantified on the Beckman HPLC system using a phase Inerstil ODS‐3 (4.6 × 150 mm, 3 µm) analytical column (GL Sciences). Analyses were performed at a flow rate of 1 mL min^−1^ at room temperature and the eluate was monitored at 220 nm. The gradient solvent system used was made of water + 0.1% TFA and ACN + 0.1% TFA. The percentage of ACN at 0, 4, and 8 min was 28, 55, and 28, respectively, and the running time was 11 min.

The amount of siTGF‐β1 and Poly(I:C) in the supernatants was determined using the Quant‐iT RNA Assay Kit (broad range), while CpG‐ODN was determined by the Quant‐iT OliGreen ssDNA Assay Kit, following manufacturer's instructions. Fluorescence generated by the binding of OliGreen reagents to CpG was measured using the fluorometer at 485 nm excitation and 520 nm emission wavelengths, while relative fluorescence for the RNA Assay kit was measured at 644 nm excitation and 673 nm emission wavelengths.

(1) $$\mathrm{EE}\left(\%\right)=\frac{\mathrm{initial}\;\mathrm{amount}\;\mathrm{of}\;\mathrm{biomolecule}-\mathrm{amount}\;\mathrm{of}\;\mathrm{biomolecule}\;\mathrm{in}\;\mathrm{the}\;\mathrm{supernatant}}{\mathrm{initial}\;\mathrm{amount}\;\mathrm{of}\;\mathrm{biomolecule}}\times 100$$

(2) $$\mathrm{LC}\left({\mu g\;\mathrm{mg}}^{-1}\right)=\frac{\mathrm{initial}\;\mathrm{amount}\;\mathrm{of}\;\mathrm{biomolecule}-\mathrm{amount}\;\mathrm{of}\;\mathrm{biomolecule}\;\mathrm{in}\;\mathrm{the}\;\mathrm{supernatant}}{\mathrm{total}\;\mathrm{amount}\;\mathrm{of}\;\mathrm{polymer}}$$

### Cell Culture Conditions

Murine bone marrow DC (JAWSII cell line [ATCC CRL‐11904]) were cultured in MEM‐α supplemented with 10% v/v FBS, 1% v/v PEST, 1% v/v sodium pyruvate, and 5 ng mL^−1^ GM‐CSF. Murine colon adenocarcinoma MC38 cells were cultured in RPMI 1640 + Glutamax supplemented with 10% v/v FBS and 1% v/v PEST. Human dermal microvascular endothelial HMEC1 cells (ATCC CRL‐3243) were cultured in MCDB 131 medium supplemented with 10% v/v FBS and 1% v/v PEST. All cells were cultured in a humidified incubator equilibrated with 5% CO_2_ at 37 °C.

### In Vitro Cell Viability and NP Internalization

JAWSII DC (3 × 10^4^ cells per well), MC38 CRC cells (6 × 10^3^ cells per well), and HMEC1 (6 × 10^3^ cells per well) were seeded in 96‐well plates and incubated overnight. Cells were then incubated with fluorescent Cy5‐labeled NP (0.5 mg mL^−1^; 646/662 nm of excitation/emission wavelengths) at different incubation time points. Cells were subsequently harvested by centrifugation, washed with PBS, and resuspended in propidium iodide solution (2 µg mL^−1^ in flow cytometry buffer (PBS with 2% v/v FBS); 535/617 nm excitation/emission wavelengths) for 15 min at room temperature, to detect dead cells. Non‐treated cells were used as negative controls. The individual fluorescence of 10 000 cells was collected for each sample using an LSR Fortessa cytometer (BD Biosciences) and analyzed with FlowJo software version 7.6.5 (TreeStar).

To evaluate the NP internalization by confocal microscopy, JAWSII DC cells (3 × 10^4^ cells per well) were seeded in 8‐well Ibidi µ‐Slide microscopy chambers and incubated overnight. Cells were incubated with fluorescent Cy5‐labeled NP (0.5 mg mL^−1^) for 6 and 24 h. Live cells were then washed and incubated with Hoechst 332 (1 µg mL^−1^) and wheat germ agglutinin Alexa Fluor 488 (5 µg mL^−1^) for 10 min to stain the nuclei and the cell membrane, respectively. Non‐treated (no NP) cells were used as the negative control. Particle internalization was analyzed by confocal microscopy using a Leica TCS SP8 (Leica Microsystems CMS GmbH, Mannheim, Germany) inverted microscope (DMi8) with a 63× oil (1.4 numerical aperture). Excitation of Hoechst, Alexa Fluor 488, and Cy5‐labeled NP was performed using 405, 488, and 638 nm diode lasers, respectively. Images were processed using Fiji software (Bethesda, USA).

### Animal Studies

Female C57BL/6J (10–13 weeks old) and Balb/c (11 weeks old) mice were purchased from Charles River or Instituto Gulbenkian de Ciência (IGC) and housed in the animal facility of the Faculty of Pharmacy at the University of Lisbon. All animal procedures were completed in compliance with the Faculty of Pharmacy, University of Lisbon guidelines. Protocols were reviewed and approved by the Portuguese competent authority for animal protection, Direção‐Geral de Alimentação e Veterinária (Reference 0421/000/000/2021). Animals were housed under a 12 h light, 12 h dark cycle, with food and water available ad libitum and handled in compliance with the NIH guidelines and the European Union rules for the care and handling of laboratory animals (Directive 2010∖63∖EU). Mice body weight change was monitored two to three times per week. Mice were euthanized according to ethical protocol when showing signs of distress or with rapid weight loss (above 10% within a few days or 20% from the initial weight). Tumor‐bearing mice were euthanized in case the tumor size exceeded 1500 mm^3^ (MC38 model) or 2000 mm^3^ (CT26 and B16F10 models), or if the tumor was necrotic or ulcerative.

### In Vivo Study of NP Internalization by Myeloid APC, and CD11b^+^CD11c^+^ Activation and Maturation in Draining LN

10 weeks old female C57BL/6J mice (*n* = 3 animals per group) were s.c. immunized at the inguinal site at both right and left flanks, with fluorescent Cy5‐labeled plain (empty) or Adpgk‐loaded NP (100 µg of Adpgk/20 µg of CpG/40 µg of Poly(I:C) per mouse; 100 µL of NP per side). PBS‐treated mice were used as negative controls. Inguinal LN were harvested 14 h post‐immunization. A single cell suspension was stained with fluorochrome‐labeled anti‐mouse antibodies against CD11b‐VioGreen (Miltenyi Biotec, Cat.# 130‐113‐811, clone: REA592, 1:50), CD11c‐FITC (Miltenyi Biotec, Cat.# 130‐110‐837, clone: REA754, 1:50), MHC Class II (I‐Ab)‐VioBlue (Miltenyi Biotec, Cat.# 130‐112‐237, clone: REA813, 1:50), CD80‐PE‐Vio 770 (Miltenyi Biotec, Cat.# 130‐116‐462, clone: REA983, 1:50), and CD86‐PE (Miltenyi Biotec, Cat.# 130‐122‐129, clone: REA1190, 1:50), for 15 min at 4 °C. Samples were analyzed using a LSR Fortessa cytometer (BD Biosciences) and FlowJo software version 7.6.5 for Microsoft (TreeStar).

### In Vivo Study of the Impact of Booster Doses on the Systemic T‐Cell Activation and Cytokine Secretion from Naïve Mice

13 weeks old female C57BL/6J mice (*n* = 3 animals per group) were s.c. immunized at the inguinal site at both right and left flanks, with Adpgk‐loaded NP (100 µg of Adpgk/20 µg of CpG/40 µg of Poly(I:C) per mouse; 100 µL of NP per side), two times, 7 days apart. PBS‐treated mice were used as negative controls. Splenocytes were harvested 7 days after the second immunization.

For the T‐cell activation assay, a single cell suspension was stained with fluorochrome‐labeled anti‐mouse antibodies against CD44‐Brilliant Violet 711 (BioLegend, Cat.# 103057, clone: IM7, 1:80), CD8a‐FITC (BioLegend, Cat.# 100706, clone: 53‐6.7, 1:50), CD4‐PerCP‐Cy5.5 (BioLegend, Cat.# 100434, clone: GK1.5, 1:80), CD107b‐Alexa Fluor 647 (BioLegend, Cat.# 108512, clone: M3/84, 1:200), and CD3‐APC/Cyanine7 (BioLegend, Cat.# 100222, clone: 17A2, 1:80), for 15 min at 4 °C.

For the cytokine priming assay, splenocytes were seeded for 6 h in a complete RPMI medium in the presence of MHCI‐Adpgk (0.25 mg mL^−1^) and MHCII‐Adpgk (0.25 mg mL^−1^) peptides and CD28 (0.002 mg mL^−1^), which was followed by the incubation with Brefeldin A (1×) for 2 h. A single cell suspension was then stained with fluorochrome‐labeled anti‐mouse antibodies against TNF‐α‐Brilliant Violet 421 (BioLegend, Cat.# 506328, clone: MP6‐XT22, 1:80), IL‐2‐Brilliant Violet 711 (BioLegend, Cat.# 503837, clone: JES6‐5H4, 1:40), IFN‐ɣ‐Brilliant Violet 785 (BioLegend, Cat.# 505838, clone: XMG1.2, 1:80), CD8a‐FITC (BioLegend, Cat.# 100706, clone: 53‐6.7, 1:50), CD4‐PerCP‐Cy5.5 (BioLegend, Cat.# 100434, clone: GK1.5, 1:80), IL‐10‐APC (BioLegend, Cat.# 505010, clone: JES5‐16E3, 1:80), and CD3‐APC/Cyanine7 (BioLegend, Cat.# 100222, clone: 17A2, 1:80), for 15 min at 4°C. Samples were analyzed using a Cytek Aurora cytometer (Cytek) and FlowJo software version 7.6.5 for Microsoft (TreeStar).

### Therapeutic Intervention Study Design

On day 0, female C57BL/6J (10–13 weeks old) or Balb/c (11 weeks old) mice were s.c. inoculated at the right flank with 0.5 × 10^6^ MC38, 0.5 × 10^6^ CT26, or 0.15 × 10^6^ B16F10 cells (100 µL), respectively, in PBS or mixed (1:1) with growth‐factor reduced Matrigel, as reported by Luo et al.^[^ ^39b^ ^]^ Prior to tumor inoculation, the hair from the right dorsal area of the mice was removed using a shaver.

For the immunization study evaluating the anti‐tumor immune‐mediated response induced by PLGA‐PEG‐Man and PLGA‐POx‐Man nanovaccine candidates and its dependence on CD8^+^ T cell‐mediated mechanism, mice were randomized into seven groups (*n* = 5 animals per group, replicated in two independent experiments for PBS, PLGA‐POx‐Man and PLGA‐PEG‐Man groups) according to Table S3, Supporting Information, on day 6 following tumor inoculation. Nanovaccines (200 µL) were administered to each mouse by hock immunization, via s.c. injection proximal to inguinal LN. Half dose was injected on the right side, while the other half was injected on the left side. The MHCI‐Adpgk and MHCII‐Adpgk peptide antigens were administered at both sides of each mouse (groups 2–7). Mice were treated with NP (10–20 mg mL^−1^, according to MHCI/II‐Adpgk loadings; 50 µL per side) containing 100 µg of Adpgk antigen (50 µg of MHCI‐Adpgk and 50 µg of MHCII‐Adpgk), 20 µg of CpG‐ODN, and 40 µg of Poly(I:C). For CD8^+^ T‐cell depletion, α‐CD8 or IgG2b isotype control mAb were administered i.p. at 10 mg kg^−1^ following the schedule in Figure 2Q.^[^ ^53^ ^]^

For intervention therapeutic studies evaluating the anti‐tumor efficacy of the combinational treatments, mice were randomized into different groups (*n* = 5–8 animals per group) according to Table S4, Supporting Information, on days 5–10 following tumor inoculation. Different schedules were used for the different intervention combinatorial studies without or with the addition of αPD‐1 (Figures 3, 5, and 6A, respectively). PBS and nanovaccine or Nanovaccine_siTGF‐β1 were s.c. administered to mice by hock immunization, via injection proximal to inguinal LN (200 µL), on days 8/10, 15/17, and 22/24 following tumor inoculation (tumor size ≈ 50–100 mm^3^). Similarly to the previous in vivo study, half dose was injected on the right side, while the other half was administered on the left side. MHCI‐Adpgk (nanovaccine), MHCI‐Adpgk/KRAS_G12D_ + siTGF‐β1 and MHCII‐Adpgk/KRAS_G12D_ (Nanovaccine_siTGF‐β1 for CRC models), and MHCII‐MUT30 + siTGF‐β1 (Nanovaccine_siTGF‐β1 for melanoma model) peptide antigens were administered at both sides of each mouse (50 µL per side). Each dose per mouse contained 100 µg of Adpgk/KRAS_G12D_ antigen (50 µg of MHCI‐Adpgk/KRAS_G12D_ and 50 µg of MHCII‐Adpgk/KRAS_G12D_) or 50 µg of MUT30 antigen, 20 µg of siTGF‐β1 (Nanovaccine_siTGF‐β1), and 20 µg of CpG‐ODN and 40 µg of Poly(I:C), entrapped into NP (10 and 12.5 mg mL^−1^ according to MHCII‐ and MHCI‐Adpgk loadings, respectively; 14.3 and 15.4 mg mL^−1^ according to MHCII‐ and MHCI‐KRAS_G12D_ loadings, respectively; or 25 mg mL^−1^ according to MHCII‐MUT30 loadings). TIME‐targeted NP containing 20 µg of siTGF‐β1, 20 µg of CpG‐ODN, and 40 µg of Poly(I:C) entrapped into NP (50 µL at 25 mg mL^−1^), were peritumorally administered, following the treatment schedule also used for nanovaccines (Figure 3A,I). Pexidartinib (TAM inhibitor) and αPD‐1 were administered i.p. at 10 mg kg^−1^.^[^ ^6^, ^18^ ^]^ Tumor size was monitored two to three times per week using a digital caliper. Tumor volume (mm^3^) was determined by$\;\frac{d^{2}\times D}{2}$ where *d* and *D* were the shortest and longest diameter in mm, respectively.

### Flow Cytometry Analysis of Immune Subsets

Tumors, spleens, and LN (*n* = 3–5 animals per group) were isolated from mice after euthanasia and homogenized in a single‐cell suspension in cold sterile PBS. Tumor single‐cell suspensions were obtained by mechanical disruption and enzymatic digestion (0.5% m/v BSA, 0.1% m/v collagenase type II, 0.1% m/v neutral protease (dispase) and powders of DNAse I in RPMI 1640 + Glutamax) of the tumor tissues, for 1 h at 37°C. After digestion, tumor single‐cell suspensions were depleted of erythrocytes using ACK lysing buffer for 5 min at 37 °C and filtered through a 70 µm with cold PBS to remove the debris. Spleens were also mechanically disrupted, and single‐cell suspensions depleted of erythrocytes were prepared and further filtered, as previously described. For the T‐cell activation and cytokine priming assay, splenocytes were seeded for 6 h in a complete RPMI medium in the presence of MHCI‐Adpgk (0.25 mg mL^−1^) and MHCII‐Adpgk (0.25 mg mL^−1^) peptides and CD28 (0.002 mg mL^−1^), which was followed by the incubation with Brefeldin A (1×) for 2 h. LN cell suspensions were obtained by mechanical disruption and filtered as described. Cells were stained with fluorochrome‐labeled anti‐mouse antibodies for surface markers, fixed with PFA 3.7% or eBioscience Fix (Thermo Fisher Scientific, Cat.# 00‐5523‐00), and permeabilized using the Inside Stain kit (Miltenyi Biotec, Cat.# 130‐090‐477) or eBioscience Foxp3/Transcription Factor Staining Buffer Set (Thermo Fisher Scientific, Cat.# 00‐5523‐00) following intracellular staining with fluorochrome‐labeled anti‐mouse antibodies, according to manufacturer's instructions. Samples were analyzed using a LSR Fortessa cytometer (BD Biosciences) or a Cytek Aurora cytometer (Cytek) and FlowJo software version 7.6.5 for Microsoft (TreeStar).

### T‐Cell Activation and Cytokine Secretion Panel

TNF‐α‐Brilliant Violet 421 (BioLegend, Cat.# 506328, clone: MP6‐XT22, 1:80), FOXP3‐eFluor 450 (eBioscience, Cat.# 48‐5773‐82, clone: FJK‐16s, 1:300), IL‐2‐Brilliant Violet 711 (BioLegend, Cat.# 503837, clone: JES6‐5H4, 1:40), IFN‐ɣ‐Brilliant Violet 785 (BioLegend, Cat.# 505838, clone: XMG1.2, 1:80), CD8a‐FITC (BioLegend, Cat.# 100706, clone: 53‐6.7, 1:50), CD4‐PerCP‐Cy5.5 (BioLegend, Cat.# 100434, clone: GK1.5, 1:80), CD25‐PE/Cy5 (BioLegend, Cat.# 102010, clone: PC61, 1:80), CD44‐PE/Cyanine7 (BioLegend, Cat.# 103029, clone: IM7, 1:80), IL‐10‐APC (BioLegend, Cat.# 505010, clone: JES5‐16E3, 1:80), CD107b‐Alexa Fluor 647 (BioLegend, Cat.# 108512, clone: M3/84, 1:200), CD3‐APC/Cyanine7 (BioLegend, Cat.# 100222, clone: 17A2, 1:80), and LIVE/DEAD Fixable Yellow Dead Cell Stain (Thermo Fisher Scientific, Cat.# L34967, 1:7500).

### TAM panel

MHC Class II‐VioBlue (Miltenyi Biotec, Cat.# 130‐112‐237, clone: REA813, 1:50), CD206 (mannose receptor)‐Brilliant Violet 711 (BioLegend, Cat.# 141727, clone: C068C2, 1:40), CD11b‐Vio Bright FITC (Miltenyi Biotec, Cat.# 130‐113‐805, clone: REA592, 1:50), CD45‐PerCP‐Vio 700 (Miltenyi Biotec, Cat.# 130‐110‐663/130‐110‐801, clone: REA737, 1:50), F4/80‐PE‐Vio 770 (Miltenyi Biotec, Cat.# 130‐118‐459, clone: REA126, 1:50), Gr1‐APC (Miltenyi Biotec, Cat.# 130‐112‐307, clone: REA810, 1:50), and LIVE/DEAD Fixable Yellow Dead Cell Stain (Thermo Fisher Scientific, Cat.# L34967, 1:1000).

### T Lymphocyte Panel

CD4‐Pacific Blue (BioLegend, Cat.# 100531, clone: RM4‐5, 1:50), CD8a‐FITC (BioLegend, Cat.# 100706, clone: 53‐6.7, 1:50), CD45‐PerCP (BioLegend, Cat.# 103130, clone: 30‐F11, 1:80), CD279 (PD‐1)‐PE (BioLegend, Cat.# 135206, clone: 29F.1A12, 1:20), CD3‐APC/Cyanine7 (BioLegend, Cat.# 100222, clone: 17A2, 1:80), and LIVE/DEAD Fixable Yellow Dead Cell Stain (Thermo Fisher Scientific, Cat.# L34967, 1:7500).

### T‐Cell Activated/Memory Panel

CD44‐Brilliant Violet 711 (BioLegend, Cat.# 103057, clone: IM7, 1:80), CD8a‐FITC (BioLegend, Cat.# 100706, clone: 53‐6.7, 1:50), CD62L‐PE (BioLegend, Cat.# 104407, clone: MEL‐14, 1:80), CX3CR1‐PE/Cyanine 7 (BioLegend, Cat.# 149015, clone: SA011F11, 1:1333), CD107b‐Alexa Fluor 647 (BioLegend, Cat.# 108512, clone: M3/84, 1:200), CD3‐APC/Cyanine7 (BioLegend, Cat.# 100222, clone: 17A2, 1:80), and LIVE/DEAD Fixable Yellow Dead Cell Stain (Thermo Fisher Scientific, Cat.# L34967, 1:7500).

### Cytokine Panel

TNF‐α‐Brilliant Violet 421 (BioLegend, Cat.# 506328, clone: MP6‐XT22, 1:80), IL‐2‐Brilliant Violet 711 (BioLegend, Cat.# 503837, clone: JES6‐5H4, 1:40), IFN‐ɣ‐Brilliant Violet 785 (BioLegend, Cat.# 505838, clone: XMG1.2, 1:80), CD8a‐FITC (BioLegend, Cat.# 100706, clone: 53‐6.7, 1:50), CD3‐APC/Cyanine7 (BioLegend, Cat.# 100222, clone: 17A2, 1:80), and LIVE/DEAD Fixable Yellow Dead Cell Stain (Thermo Fisher Scientific, Cat.# L34967, 1:7500).

### GC/Tfh/Tfr Panel

FOXP3‐eFluor 450 (eBioscience, Cat.# 48‐5773‐82, clone: FJK‐16s, 1:333), CD95 (Fas)‐Brilliant Violet 605 (BioLegend, Cat.# 152612, clone: SA367H8, 1:80), CD185 (CXCR5)‐Brilliant Violet 711 (BioLegend, Cat.# 145529, clone: L138D7, 1:40), CD4‐PerCP/Cyanine5.5 (BioLegend, Cat.# 100434, clone: GK1.5, 1:80), CD279 (PD‐1)‐PE (BioLegend, Cat.# 135206, clone: 29F.1A12, 1:20), CD25‐PE/Cy5 (BioLegend, Cat.# 102010, clone: PC61, 1:80), GL7‐eFluor 660 (eBioscience, Cat.# 50‐5902‐82, clone: GL7, 1:80), CD19‐APC/Cyanine7 (BioLegend, Cat.# 115530, clone: 6D5, 1:20), and LIVE/DEAD Fixable Yellow Dead Cell Stain (Thermo Fisher Scientific, Cat.# L34967, 1:7500).

### TGF‐β1 and CSF‐1R Expression in Tumors

Tumor samples isolated from mice (*N* = 2–3) of first COMBI assay were frozen in dry ice immediately post‐euthanasia to quantify the TGF‐β1 and CSF‐1R expression, at both mRNA and protein levels.

### RT‐PCR

Total RNA was extracted from mice tumor samples using the TRIzol reagent according to the manufacturer's instructions. Total RNA was quantified using Nanodrop ND‐1000 Spectrophotometer (NanoDrop Technologies, Thermo Fisher Scientific) and converted into cDNA using NZY First‐Strand cDNA Synthesis Kit (NZYTech), according to the manufacturer's instructions. Quantitative real‐time RT‐PCR (qPCR) was performed in the QuantStudio 7 Flex Real‐Time PCR System (Applied Biosystems, Thermo Fisher Scientific). Primers for *Tgf‐β1* gene (sequences: 5′ CTG CTG ACC CCC ACT GAT AC 3′ [forward] and 5′ GTG AGC GCT GAA TCG AAA GC 3′ [reverse]) and for the hypoxanthine phosphoribosyltransferase (*Hprt*) gene (sequences: 5′ GGT GAA AAG GAC CTC TCG AAG TG 3′ [forward] and 5′ ATA GTC AAG GGC ATA TCC AAC AAC A 3′ [reverse]) were used. Two independent reactions for each primer set were assessed in a total volume of 5 µL containing 2× SensiFAST SYBR Hi‐ROX kit (Bioline, Meridian Bioscience, Inc.) and 0.6 µm (each) primer. The relative amount of *Tgf‐β1* was calculated based on the standard curve and was normalized to the level of *Hprt*, being expressed as fold change related to PBS control.

### Protein Extraction and Immunoblotting

For total protein isolation, tumor samples were homogenized using a motor‐driven grinder on ice‐cold lysis buffer (10 mm Tris‐HCl [pH 7.6], 5 mm MgCl_2_, 1.5 mm potassium acetate, % Nonidet P‐40, and 2 mm dithiothreitol), 1× halt protease, and phosphatase inhibitor cocktail. The lysate was sonicated at 80% power for 10 s and centrifuged at 10 000 × *g* for 10 min at 4°C. The supernatants were recovered and stored at – 80°C. Protein concentrations were determined using the Bio‐Rad protein assay kit (Bio‐Rad Laboratories), according to the manufacturer's instructions. 30 µg of total protein extracts were separated on a 10% SDS‐PAGE. Following electrophoretic transfer onto nitrocellulose membranes and blocking with 5% m/v milk solution, blots were incubated overnight at 4°C with primary rabbit polyclonal antibody against TGF‐β1 (1:1000), p‐M‐CSF1R (1:1000), or M‐CSF1R (1:1000), and with a secondary antibody conjugated with horseradish peroxidase (1:5000 in blocking solution) for 2 h at room temperature. Membranes were processed for protein detection using Immobilon Western Chemiluminescent HRP Substrate (Millipore, Merck Life Science S.L.U.) on a ChemiDoc XRS+ imaging system (Bio‐Rad). β‐actin (1:40 000) was used as a loading control. The relative intensities of protein bands were analyzed using the Image Lab densitometric analysis software (version 5.1, Bio‐Rad Laboratories).

### Functional Assessment of Antigen‐Specific T cells

For the ELISpot assay, mice were randomized into different groups (*n* = 5 animals per group) according to Table S4, Supporting Information, and treated according to the schedule described in Figure 3I. On day 19, mice were euthanized, spleens harvested and splenocytes isolated. Splenocytes (2 × 10^5^ cells per well) were seeded for 20 h in 96‐well plates coated with the IFN‐γ antibody (R&D Systems Inc.) in the presence of MHCI/MHCII‐Adpgk peptides (1 mg mL^−1^) and CD28 (0.002 mg mL^−1^). The secreted and captured IFN‐γ was subsequently detected using a biotinylated antibody specific for IFN‐γ and an alkaline‐phosphatase conjugated to streptavidin. After the addition of the substrate solution, a blue precipitate formed and appeared as spots at the sites of cytokine localization. Automated spots were revealed using Cytation 7 (Biotek).

For the functional assessment of antigen‐specific T cells by tetramer staining assay, female C57BL/6J, 9 weeks old mice (*n* = 3 animals per group) were treated according to the second combinatorial scheme (Table S3, Supporting Information). Spleens were harvested 10 and 11 days after the first and third immunizations, respectively, and homogenized in a single‐cell suspension. Cells were incubated with FcR blocking reagent (Miltenyi Biotec) for 10 min at 4 °C and stained with the H‐2D^b^‐restricted Adpgk_R304M_ (ASMTN[R/M]ELM) PE‐labeled tetramer (NIH Tetramer Core Facility), for 30 min at 4 °C. Cells were subsequently washed to remove unbound tetramer, centrifuged at 1300 r.p.m. for 10 min at 4 °C, and resuspended in anti‐PE microbeads (Miltenyi Biotec). A QuadroMACS separator (Miltenyi Biotec) was used for the magnetic enrichment of the samples, following the manufacturer's instructions. Cells were plated in 96‐well plates and incubated with LIVE/DEAD fixable yellow dead cell stain dye. After 30 min, cells were stained with fluorochrome‐labeled anti‐mouse antibodies CD3‐APC‐Vio 770 (Miltenyi Biotec, Cat.# 130‐119‐793, clone: REA641, 1:50), CD19‐APC (Miltenyi Biotec, Cat.# 130‐112‐036, clone: REA749, 1:50), and CD8‐PE‐Vio 770 (Miltenyi Biotec, Cat.# 130‐118‐946, clone: REA601, 1:50), for 15 min at 4 °C, protected from light. Cells were washed, centrifuged, and resuspended in a flow cytometry buffer to assess the percentage of Adpgk‐specific CD3^+^ CD8^+^ T cells by using a LSR Fortessa cytometer (BD Biosciences) and FlowJo software version 7.6.5 for Microsoft (TreeStar).

### Enzyme‐Linked Immunosorbent Assay

Enzyme‐linked immunosorbent assay was performed to detect Adpgk‐specific antibodies in the serum of mice treated according to the schedule described in Figure 3I. Corning High binding 96‐well plates were precoated with MHCII‐Adpgk peptide (10 µg mL^−1^) overnight at 4 °C in carbonate buffer (pH 9.6). Plates were washed three times with PBS + 0.05% Tween‐20 (PBS‐T) and blocked with 3% BSA in PBS‐T for 2 h at 37 °C. After three washes with PBS‐T, plates were incubated with diluted (1:135) mouse serum in PBS‐T/1% BSA for 1 h at 24 °C. Following washing, Peroxidase AffiniPure Goat Anti‐Mouse IgG was added for 1 h at 24 °C. The plates were washed with PBS‐T and reactions were developed with TMB. The reaction was stopped by adding 0.5 m of sulfuric acid. Plates were read at 405 nm absorbance using the Varioskan Lux Reader (Thermo Fisher Scientific).

### Hematological and Biochemical Analysis

Blood was collected by cardiac puncture. Part of the blood was centrifuged at 13 000 r.p.m. for 20 min at 4 °C to obtain the serum and the remaining blood was dropped into EDTA tubes. Serum and blood samples were delivered to DNAtech (Portugal) to be analyzed. A serum biochemical study was performed to evaluate the activity of AST, ALT, and GGT, known as liver function markers. Urea and creatinine levels in serum were also assessed as markers of kidney function.

### Histopathological Analysis

The major organs (heart, liver, kidney, and spleen) were recovered post‐animal euthanasia. Tissues were fixed in 4% buffered formaldehyde solution for 24 h at 4 °C, processed overnight using a Tissue HistoCore Pearl (Leica), and embedded in paraffin (Cat.# 39602012, Leica). Paraffin blocks were sectioned into slides, each one with two sections 3 µm thickness, using a microtome (Minot Microtome Leica RM2145). Slides were stained with hematoxylin (Cat.# 0506004E. Bio‐optica) and eosin (Cat.# 110132‐1L, Sigma‐Aldrich) (H&E) for morphological examination and histopathological analysis (IGC).

### Statistical Methods

Sample sizes (*n*) were selected based on preliminary data from pilot experiments. Accordingly, group sizes of three animals per group were used for DC maturation and T‐cell activation studies and five to eight animals per group for therapeutic assays. Data were presented as mean ± standard deviation (s.d.) and mean ± standard error of the mean (s.e.m.) for in vitro and in vivo assays, respectively. Statistical significance was assessed by the Student's *t*‐test, one‐way and two‐way analysis of variance (ANOVA), followed by Tukey and Dunnett multiple comparisons post‐hoc test for multiple comparisons, using GraphPad Prism 6, 8, or 9 (GraphPad Software, Inc.). The statistical analysis for overall survival was determined with a log‐rank test using GraphPad Prism 6, 8, or 9 (GraphPad Software, Inc.). *p* < 0.05 were considered statistically significant.

## Conflict of Interest

Prof. R.S.‐F. is a board director at Teva Pharmaceutical Industries Ltd. All other authors declare no conflict of interest.

## Author Contributions

A.I.M., R.S.‐F., and H.F.F. conceived and designed the research. A.I.M., C.P., B.C., L.I.F.M., and R.C.A. performed the research, with the contribution of T.V., E.W., F.M.F.S., A.M.‐B., A.S.V., P.M.P.G., L.C.S., and M.B.A. for specific tasks. A.I.M., R.S.‐F., and H.F.F. wrote the manuscript. A.I.M., D.A., C.M.P.R., L.G., R.J., R.S.‐F., and H.F.F. revised the manuscript and provided comments. R.S.‐F. and H.F.F. supervised the project, overseeing the overall direction and planning of this study.

## Supporting information

Supporting Information

## Data Availability

The data that support the findings of this study are available in the supplementary material of this article.
