# Supplementary material for: Polyoxazoline‐Based Nanovaccine Synergizes with Tumor‐Associated Macrophage Targeting and Anti‐PD‐1 Immunotherapy against Solid Tumors
Source: Adv Sci (Weinh). 2023 Jul 11;10(25):2300299. doi: 10.1002/advs.202300299 (PMC10477894; doi:10.1002/advs.202300299)
Supplement: Supplementary file 1 — Supporting Information [file ADVS-10-2300299-s001.pdf]

ADVANCED SCIENCE
Open Access

Supporting Information

for *Adv. Sci.*, DOI 10.1002/advs.202300299

Polyoxazoline-Based Nanovaccine Synergizes with Tumor-Associated Macrophage Targeting and Anti-PD-1 Immunotherapy against Solid Tumors

Ana I. Matos, Carina Peres, Barbara Carreira, Liane I. F. Moura, Rita C. Acúrcio, Theresa Vogel, Erik Wegener, Filipa Ribeiro, Marta B. Afonso, Fábio M. F. Santos, Águeda Martínez-Barriocanal, Diego Arango, Ana S. Viana, Pedro M. P. Góis, Liana C. Silva, Cecília M. P. Rodrigues, Luis Graca, Rainer Jordan, Ronit Satchi-Fainaro and Helena F. Florindo**

Polyoxazoline-based nanovaccine synergizes with tumor-associated macrophage targeting and anti-PD-1 immunotherapy against solid tumors

Ana I. Matos, Carina Peres, Barbara Carreira, Liane I. F. Moura, Rita C. Acúrcio, Theresa Vogel, Erik Wegener, Filipa Ribeiro, Marta B. Afonso, Fábio M. F. Santos, Águeda Martínez-Barriocanal, Diego Arango, Ana S. Viana, Pedro M. P. Góis, Liana C. Silva, Cecília M. P. Rodrigues, Luis Graca, Rainer Jordan, Ronit Satchi-Fainaro, and Helena F. Florindo**

A. I. Matos, C. Peres, B. Carreira, L. I. F. Moura, R. C. Acúrcio, M. B. Afonso, F. M. F. Santos, P. M. P. Góis, L. C. Silva, C. M. P. Rodrigues, H. F. Florindo
Research Institute for Medicines (iMed.ULisboa), Faculty of Pharmacy, Universidade de Lisboa
1649-003 Lisboa, Portugal
E-mail: hflorindo@ff.ulisboa.pt

A. I. Matos, C. Peres, F. Ribeiro, L. Graca
Instituto de Medicina Molecular (IMM), Faculdade de Medicina, Universidade de Lisboa
1649-028 Lisboa, Portugal

T. Vogel, E. Wegener, R. Jordan
Department Chemie, Technische Universität Dresden
01062 Dresden, Germany

A. Martínez-Barriocanal, D. Arango
Group of Biomedical Research in Digestive Tract Tumors, CIBBIM-Nanomedicine, Vall d'Hebron Research Institute (VHIR), Universitat Autònoma de Barcelona (UAB)
08035 Barcelona, Spain
Group of Molecular Oncology, IRBLleida
25198, Lleida, Spain

A. S. Viana
Centro de Química Estrutural, Institute of Molecular Sciences, Departamento de Química e Bioquímica, Faculdade de Ciências, Universidade de Lisboa
1749-016 Lisboa, Portugal

R. Satchi-Fainaro
Department of Physiology and Pharmacology, Sackler Faculty of Medicine, Tel Aviv University
Sagol School of Neuroscience, Tel Aviv University
Tel Aviv 69978, Israel
E-mail: ronitsf@tauex.tau.ac.il

SUPPLEMENTARY TABLES

Table S1. Physicochemical properties and loading capacity of nanocarriers. Particle size, polydispersity index (PdI) and ζ Potential, and Entrapment Efficiency (EE) and Loading Capacity (LC) for combinations of Adpgk MC38 / $KRAS_{G12D}$ CT26 MHC class I and MHC class II neoantigen peptides, with siTGF-β1 and TLR ligands.

				Adpgk neoantigen		Poly(I:C) + siTGF-β1	
**NP**	**Size (nm ± SD)**	**PdI ± SD**	**ζ Potential (mV ± SD)**	**EE (% ± SD)**	**LC (μg mg^{-1} ± SD)**	**EE (% ± SD)**	**LC (μg mg^{-1} ± SD)**
PLGA-PEG (100% m/m)	180 ± 19	0.17 ± 0.03	-5.87 ± 0.53				
PLGA-PEG (10% m/m)	214 ± 11	0.09 ± 0.01	-0.89 ± 0.11				
PLGA-PEG-Man (10% m/m)	217 ± 10	0.12 ± 0.02	-0.98 ± 0.07				
**PEG-Man nanovaccine:** PLGA-PEG-Man (20% m/m)	216 ± 8	0.11 ± 0.03	-0.86 ± 0.22				
MHCI-Adpgk	231 ± 16	0.17 ± 0.01	-2.46 ± 1.20	55.6 ± 1.1[a]	27.8 ± 0.6[a]		
MHCII-Adpgk	239 ± 5	0.16 ± 0.05	-6.64 ± 1.07	70.5 ± 1.4[a]	35.2 ± 0.7[a]		
PLGA-PEG-Man (30% m/m)	218 ± 21	0.14 ± 0.01	-0.88 ± 0.11				
PLGA-POx (10% m/m)	204 ± 8	0.15 ± 0.02	-0.95 ± 0.21				
PLGA-POx (20% m/m)	185 ± 17	0.09 ± 0.01	-0.92 ± 0.16				
PLGA-POx (30% m/m)	164 ± 4	0.11 ± 0.01	-0.94 ± 0.14				
**POx-Man nanovaccine:** PLGA-POx-Man (20% m/m)	194 ± 18	0.11 ± 0.02	-0.80 ± 0.03				
MHCI-Adpgk	211 ± 17	0.18 ± 0.05	-7.86 ± 1.12	78.3 ± 8.4[a]	39.1 ± 4.2[a]	96.1 ± 2.5	30.8 ± 0.8
MHCII-Adpgk	189 ± 13	0.13 ± 0.02	-6.23 ± 1.16	97.2 ± 4.4[a]<br>77.5 ± 6.6[b]	48.6 ± 2.2[a]<br>38.7 ± 3.3[b]		
MHC I-$KRAS_{G12D}$	190 ± 11	0.14 ± 0.04	-1.18 ± 0.54	64.6 ± 9.4[a]	32.3 ± 4.7[a]		
MHC II-$KRAS_{G12D}$	187 ± 14	0.10 ± 0.03	-1.32 ± 0.56	70.4 ± 6.9[a]	35.2 ± 3.5[a]		
MHC II-MUT30	203 ± 10	0.13 ± 0.05		18.9 ± 5.6[a]	18.9 ± 5.6[a]		
PLGA-POx-RGD (10% m/m)	202 ± 11	0.12 ± 0.03	-0.77 ± 0.24				
**TIME-targeted NP:** PLGA-POx-RGD (30% m/m)	193 ± 12	0.19 ± 0.03	-10.1 ± 2.60			96.1 ± 0.6	46.1 ± 0.3

DC: dendritic cell; EE: entrapment efficiency; LC: loading capacity; Man: mannose; MHC: major histocompatibility complex; NP: nanoparticle; ODN: oligodeoxynucleotides; PdI, polydispersity index; PEG: poly(ethylene glycol); Poly(I:C): polyinosinic:polycytidylic acid; PLGA: poly(lactic-co-glycolic) acid; POx: poly(2-oxazoline); RGD: tripeptide motif Arg-Gly-Asp; SD: standard deviation; siRNA: small interfering RNA; siTGF-β1: siRNA anti-TGF-β1; TIME: tumor-immune microenvironment; TLR: toll-like receptor. **PEG/POx-Man nanovaccine:** NP loading MHCI/II-Adpgk / $KRAS_{G12D}$ / MUT30, TLR ligands (CpG, Poly(I:C)), with or without siTGF-β1. **TIME-targeted NP:** NP loading TLR ligands (CpG, Poly(I:C)) and siTGF-β1. For each formulation, at least 3 independent batches ($N \geq 3$) were prepared and measured in triplicate ($n = 3$). [a] EE and LC were quantified using fluorescamine assay. [b] EE and LC quantified using High-Performance Liquid Chromatography (HPLC) method.

Table S2. Polymeric blends and biomolecules co-entrapped in NP formulations.

NP	Polymeric blend	CRC antigen	TLR ligands	Gene regulator
PLGA-PEG (100% m/m)	PLGA-PEG (100% m/m)			
PLGA-PEG (10% m/m)	PLGA (90% m/m) / PLGA-PEG (10% m/m)			
PLGA-PEG-Man (10% m/m)	PLGA (90% m/m) / PLGA-PEG-Man (10% m/m)			
**PEG-Man nanovaccine:** PLGA-PEG-Man (20% m/m)	PLGA (80% m/m) / PLGA-PEG-Man (20% m/m)	MHCI/II-Adpgk	CpG-OND / Poly(I:C)	
PLGA-PEG-Man (30% m/m)	PLGA (70% m/m) / PLGA-PEG-Man (30% m/m)			
PLGA-POx (10% m/m)	PLGA (90% m/m) / POx (10% m/m)			
PLGA-POx (20% m/m)	PLGA (80% m/m) / POx (20% m/m)			
PLGA-POx (30% m/m)	PLGA (70% m/m) / POx (30% m/m)			
**POx-Man nanovaccine:** PLGA-POx-Man (20% m/m)	PLGA (80% m/m) / POx-Man (20% m/m)	MHCI/II-Adpgk / KRAS$_{G12D}$ / MUT30	CpG-OND / Poly(I:C)	siTGF-β1
PLGA-POx-RGD (10% m/m)	PLGA (90% m/m) / POx-RGD (10% m/m)			
**TIME-targeted NP:** PLGA-POx-RGD (30% m/m)	PLGA (70% m/m) / POx-RGD (30% m/m)		CpG-OND / Poly(I:C)	siTGF-β1

CpG: cytosine phosphorothioate-guanine motifs; CRC: colorectal cancer; Man: mannose; MHC: major histocompatibility complex; NP: nanoparticle; ODN: oligodeoxynucleotides; PEG: poly(ethylene glycol); PLGA: poly(lactic-co-glycolic) acid; Poly(I:C): polyinosinic:polycytidylic acid; POx: poly(2-oxazoline); RGD: tripeptide motif Arg-Gly-Asp; siRNA: small interfering RNA; siTGF-β1: siRNA anti-TGF-β1; TLR: toll-like receptor. **PEG/POx-Man nanovaccine:** NP loading MHCI/II-Adpgk / KRAS$_{G12D}$ / MUT30, TLR ligands (CpG, Poly(I:C)), with or without siTGF-β1. **TIME-targeted NP:** NP loading TLR ligands (CpG, Poly(I:C)) and siTGF-β1.

Table S3. Immunization study design.

<table>
<tr><th>Group</th><th>Treatment</th><th>Entrapped biomolecules</th><th>Route of administration</th></tr>
<tr><td>1</td><td>PBS</td><td></td><td></td></tr>
<tr><td>2</td><td>PLGA-PEG-Man nanovaccine</td><td rowspan="6">MHCI/II-Adpgk + CpG-ODN + Poly(I:C)</td><td rowspan="2">s.c.</td></tr>
<tr><td>3</td><td>PLGA-POx-Man nanovaccine</td></tr>
<tr><td>4</td><td>PLGA-PEG-Man nanovaccine + α-CD8 mAb</td><td rowspan="4">s.c. + i.p.</td></tr>
<tr><td>5</td><td>PLGA-POx-Man nanovaccine + α-CD8 mAb</td></tr>
<tr><td>6</td><td>PLGA-PEG-Man nanovaccine + IgG2b isotype control mAb</td></tr>
<tr><td>7</td><td>PLGA-POx-Man nanovaccine + IgG2b isotype control mAb</td></tr>
</table>

CpG: cytosine phosphorothioate-guanine motifs; IgG: immunoglobulin G; i.p.: intraperitoneal; mAb: monoclonal antibody; MHC: major histocompatibility complex; NP: nanoparticle; ODN: oligodeoxynucleotides; PBS: phosphate-buffered saline; Poly(I:C): polyinosinic:polycytidylic acid; s.c.: subcutaneous; TLR: toll-like receptor. Nanovaccines: NP loading MHCI/II-Adpgk and TLR ligands (CpG-ODN, Poly(I:C)).

Table S4. Therapeutic groups and administration routes for combinatorial assays.

Assays	Group	Treatment	Route of administration
1st COMBI	1	PBS	s.c.
	2	Pexidartinib (TAM inhibitor)	i.p.
	3	Nanovaccine (MHCI-Adpgk / MHCII-Adpgk)	s.c.
	4	Nanovaccine + TIME-targeted NP (siTGF-β1)	s.c. + i.t.
	5	Nanovaccine_siTGF-β1 (MHCI-Adpgk + siTGF-β1 / MHCII-Adpgk) + TIME-targeted NP (siTGF-β1)	s.c. + i.t.
	6	Nanovaccine_siTGF-β1 + TIME-targeted NP (siTGF-β1) + Pexidartinib	s.c. + i.t. + i.p.
2nd COMBI	1	PBS	s.c.
	2	Free vaccine_siTGF-β1 (MHCI-Adpgk + siTGF-β1 / MHCII-Adpgk)	s.c.
	3	Free vaccine_siTGF-β1 + Pexidartinib (TAM inhibitor)	s.c.+ i.p.
	4	Nanovaccine (MHCI-Adpgk / MHCII-Adpgk)	s.c.
	5	Nanovaccine_siTGF-β1 (MHCI-Adpgk + siTGF-β1 / MHCII-Adpgk)	s.c.
	6	Nanovaccine_siTGF-β1 + TIME-targeted NP (siTGF-β1) + Pexidartinib	s.c. + i.t. + i.p.
	7	Nanovaccine_siTGF-β1 + Pexidartinib	s.c + i.p.
	8	Nanovaccine_siTGF-β1 + TIME-targeted NP (siTGF-β1)	s.c. + i.t.
	9	TIME-targeted NP (siTGF-β1) + Pexidartinib	i.t. + i.p.
	10	TIME-targeted NP (siTGF-β1)	i.t.
3rd COMBI	1	PBS	s.c.
	2	Nanovaccine_siTGF-β1 (MHCI-Adpgk + siTGF-β1 / MHCII-Adpgk) + Pexidartinib + αPD-1	s.c + i.p + i.p.
	3	Free vaccine_siTGF-β1 + Pexidartinib + αPD-1	s.c + i.p + i.p.
4th COMBI	1	PBS	s.c.
	2	Nanovaccine_siTGF-β1 (MHCI-$KRAS_{G12D}$ + siTGF-β1 / MHCII-$KRAS_{G12D}$) + Pexidartinib + αPD-1	s.c + i.p + i.p.
	3	Free vaccine_siTGF-β1 + Pexidartinib + αPD-1	s.c + i.p + i.p.
	4	Pexidartinib + αPD-1	i.p + i.p.
5th COMBI	1	PBS	s.c.
	2	Nanovaccine_siTGF-β1 (MHCII-MUT30 + siTGF-β1) + Pexidartinib + αPD-1	s.c + i.p + i.p.
	3	Free vaccine_siTGF-β1 + Pexidartinib + αPD-1	s.c + i.p + i.p.
	4	Pexidartinib + αPD-1	s.c.

COMBI: combination; CpG: cytosine phosphorothioate-guanine motifs; i.p.: intraperitoneal; i.t.: intratumoral; MHC: major histocompatibility complex; NP: nanoparticle; ODN: oligodeoxynucleotides; PBS: phosphate-buffered saline; Poly(I:C): polyinosinic:polycytidylic acid; s.c.: subcutaneous; siRNA: small interfering RNA; siTGF-β1: siRNA anti-TGF-β1; TLR: toll-like receptor. Nanovaccine: NP loading MHCI/II-Adpgk and TLR ligands (CpG-ODN, Poly(I:C)). TIME-targeted NP: NP

loading siTGF-β1 and TLR ligands (CpG-ODN, Poly(I:C)). Nanovaccine_siTGF-β1 for colorectal cancer models: NP loading MHCI-Adpgk/KRAS$_{G12D}$ + siTGF-β1 / MHCII-Adpgk/KRAS$_{G12D}$ and TLR ligands (CpG-ODN, Poly(I:C)). Nanovaccine_siTGF-β1 for melanoma model: NP loading MHCII-MUT30 + siTGF-β1 and TLR ligands (CpG-ODN, Poly(I:C)).

SUPPLEMENTARY FIGURES

A

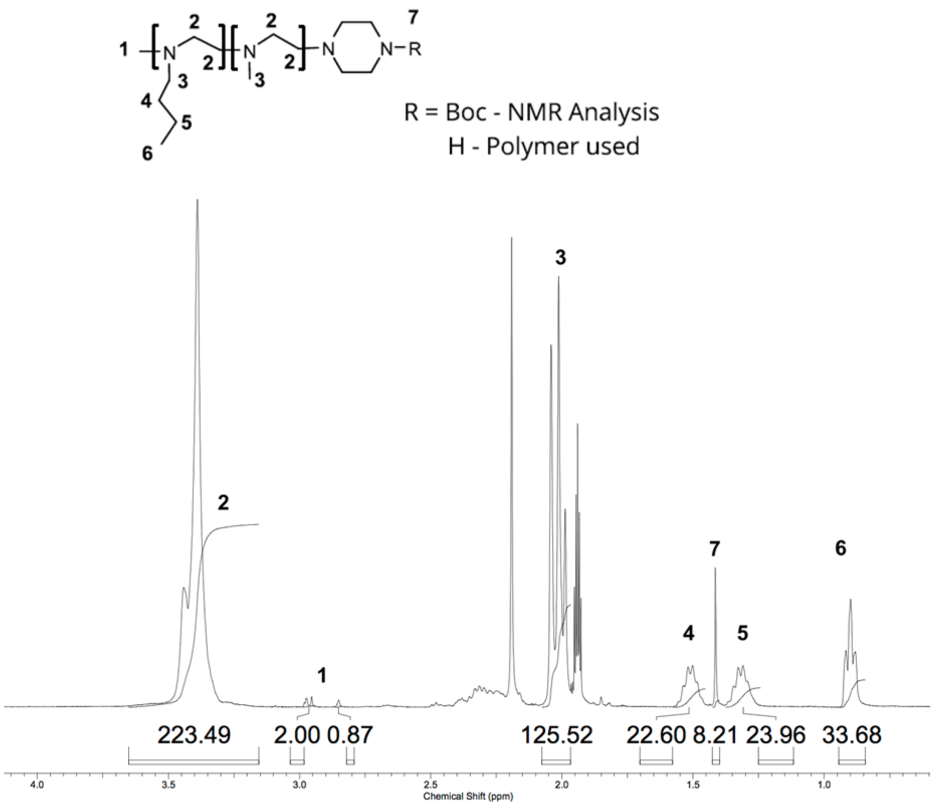

B

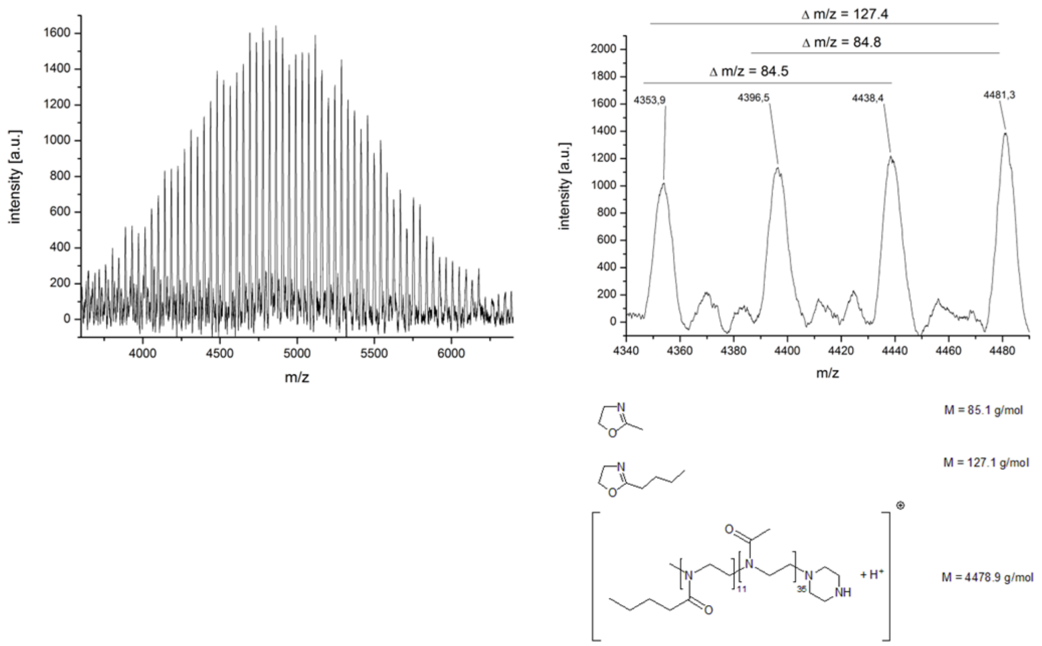

C

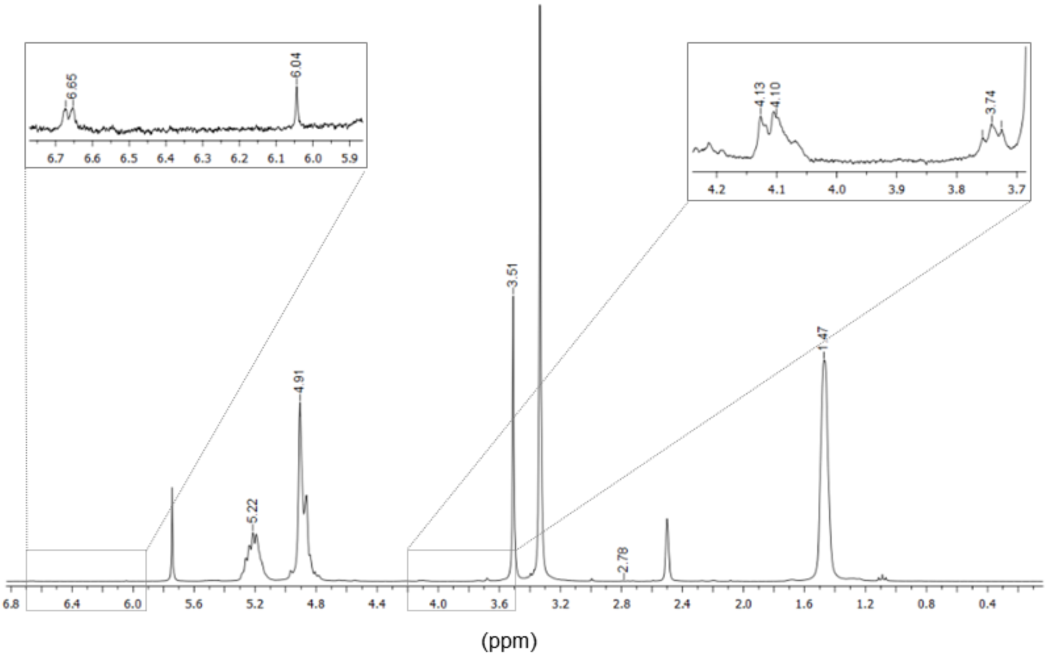
6.65
6.04
4.13
4.10
3.74
3.51
4.91
5.22
2.78
1.47
(ppm)

D

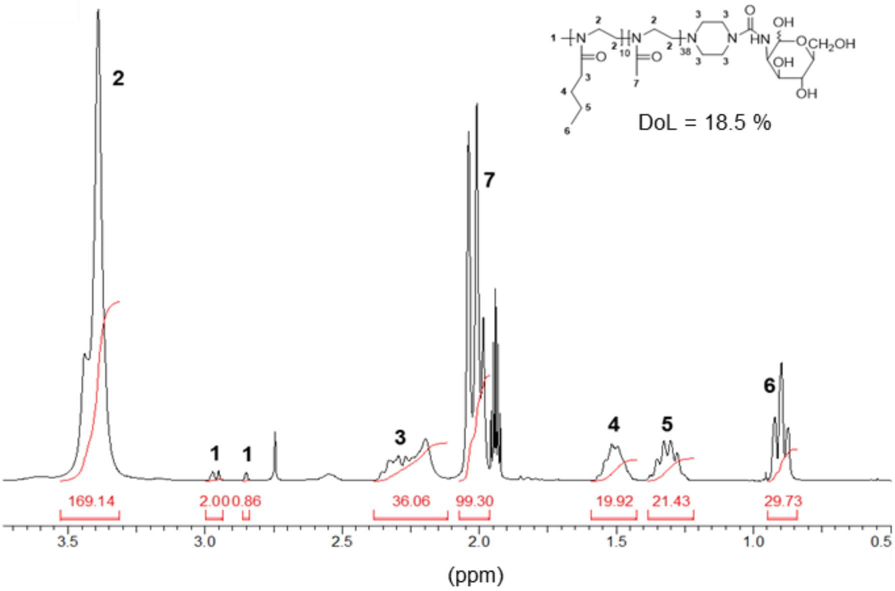
DoL = 18.5 %
169.14
2.00
0.86
36.06
99.30
19.92
21.43
29.73
(ppm)

E

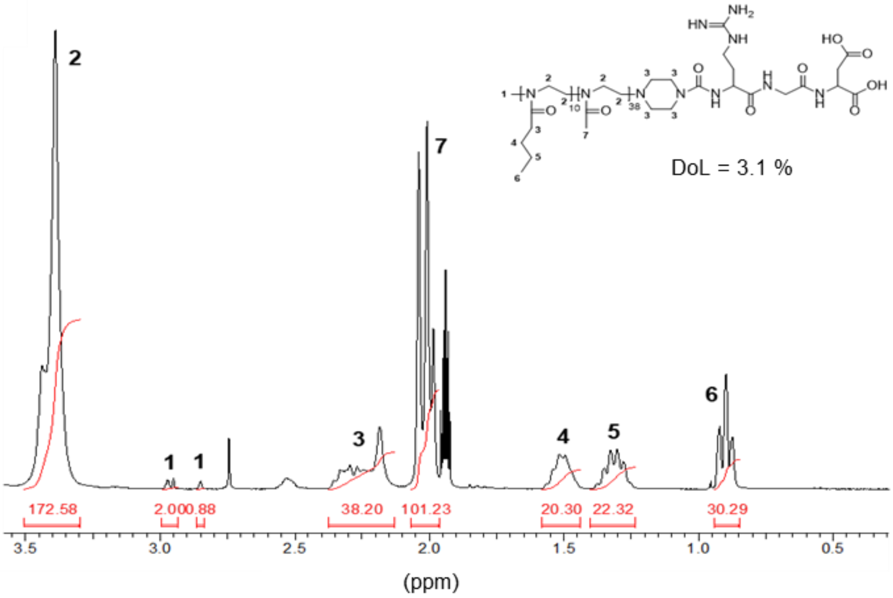
DoL = 3.1 %
172.58
2.00
0.88
38.20
101.23
20.30
22.32
30.29
(ppm)

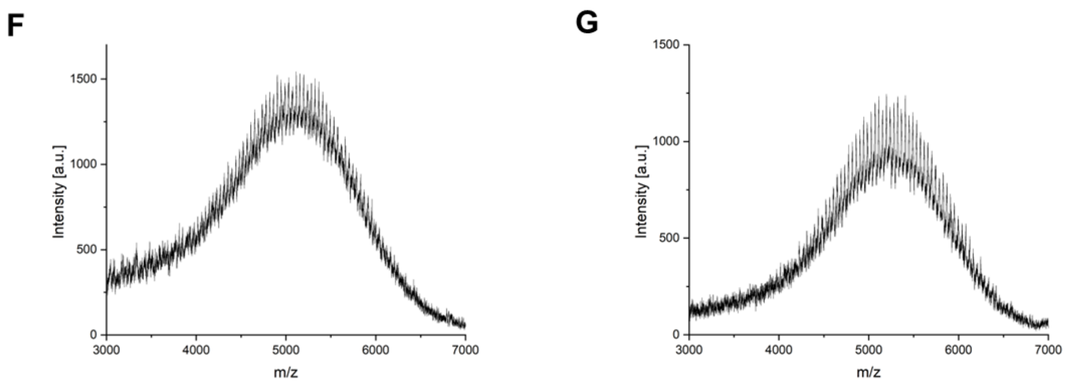

Figure S1. (**A**) ^{1}H-NMR spectrum (ACN-d3) and structure of poly(2-methyl-2-oxazonline-*block*-2-butyl-oxazoline) (POx). (**B**) MALDI spectra and peak assignment of POx. (**C** to **E**) ^{1}H-NMR spectra of PLGA-PEG-Man in DMSO-d6 (**C**), POx-Man in ACN-d3 (**D**), and POx-RGD in ACN-d3 (**E**). (**F** and **G**) MALDI-TOF spectra of POx-Man (**F**) and POx-RGD (**G**).

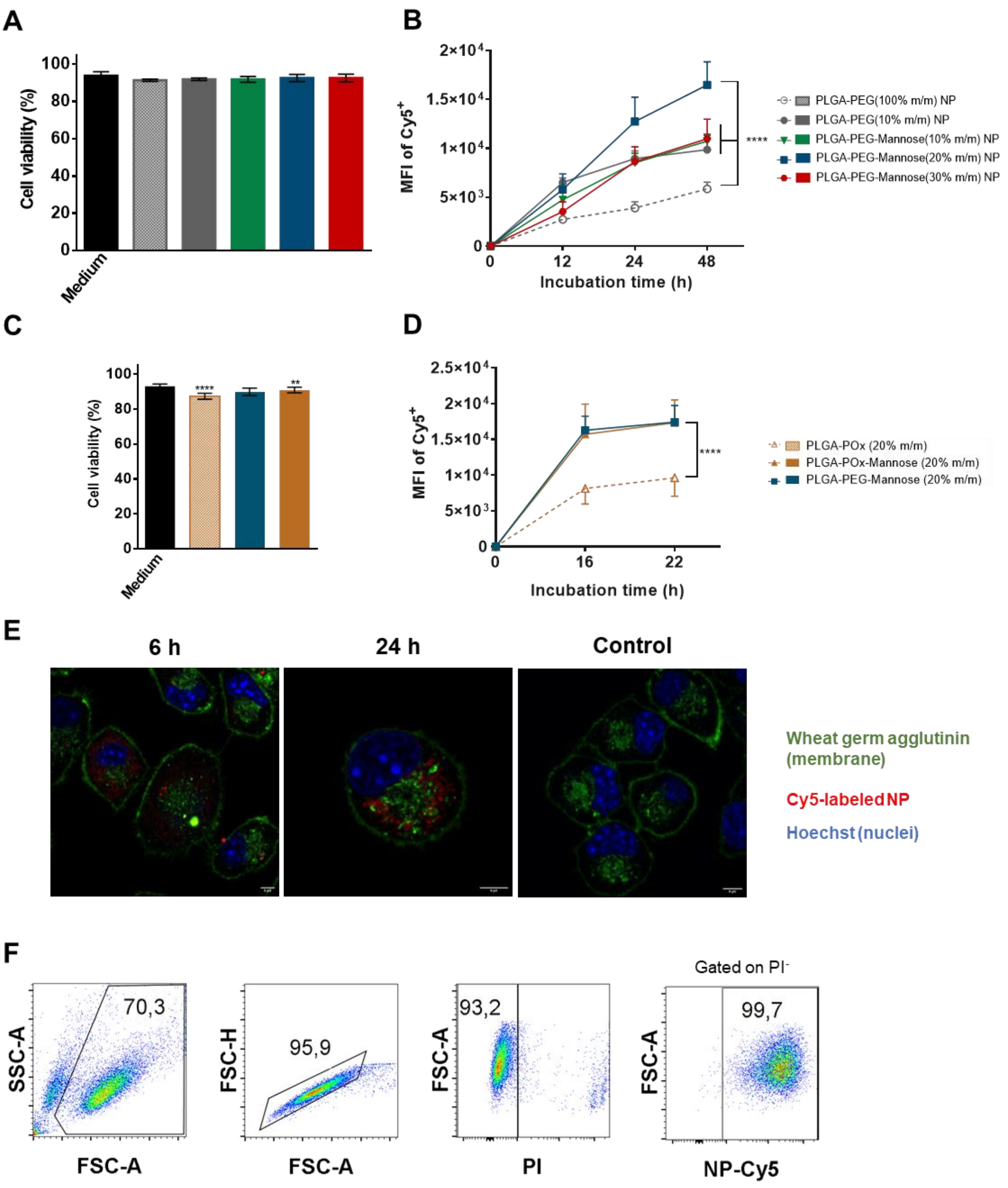

Figure S2. Mannose-functionalized NP are biocompatible and efficiently internalized by DC. Flow cytometry analysis of cell viability (**A** and **C**) and NP internalization (**B** and **D**) by murine immature DC (JAWSII) after incubation of (**A**) and (**B**) mannose-functionalized PEGylated NP, at different percentages of mannose, for 48 h; and (**C**) and **D**) mannose-functionalized POx NP, for 22 h. Non-treated cells and non-targeted (no targeting moiety) NP were used as controls. Data are presented as mean ± s.d., N = 3, independent experiments (cell line passages) performed with duplicates of NP (n = 2) (**A** and **B**) and N = 2, independent experiments (cell line passages), performed with triplicates of NP (n = 3) (**C** and **D**), with three technical replicates per NP. **P < 0.01, ****P < 0.0001, analyzed by one-way (**A** and **C**) and two-way (**B** and **D**) analysis of variance (ANOVA) with Tukey multiple comparisons post-hoc test. (**E**) Confocal images of DC (JAWSII) after 6 (left) and 24 (middle) hours of incubation with mannose-functionalized POx NP. Cells with

no NP (right) were used as control. Nuclei (blue) and plasma membrane (green) were stained with Hoechst 332 and wheat germ agglutinin Alexa Fluor 488, respectively. NP (red) were labeled with Cy5. Z-stacks of N = 2, independent experiments performed with triplicates of NP (n = 3). Scale bars, 5 µm. (**F**) Representative gating strategy used for (**A**) and (**C**) cell viability (PI^-) and (**B**) and (**D**) NP internalization (fluorescent NP-$Cy5^+$ (APC-Cy7 channel)) analysis.

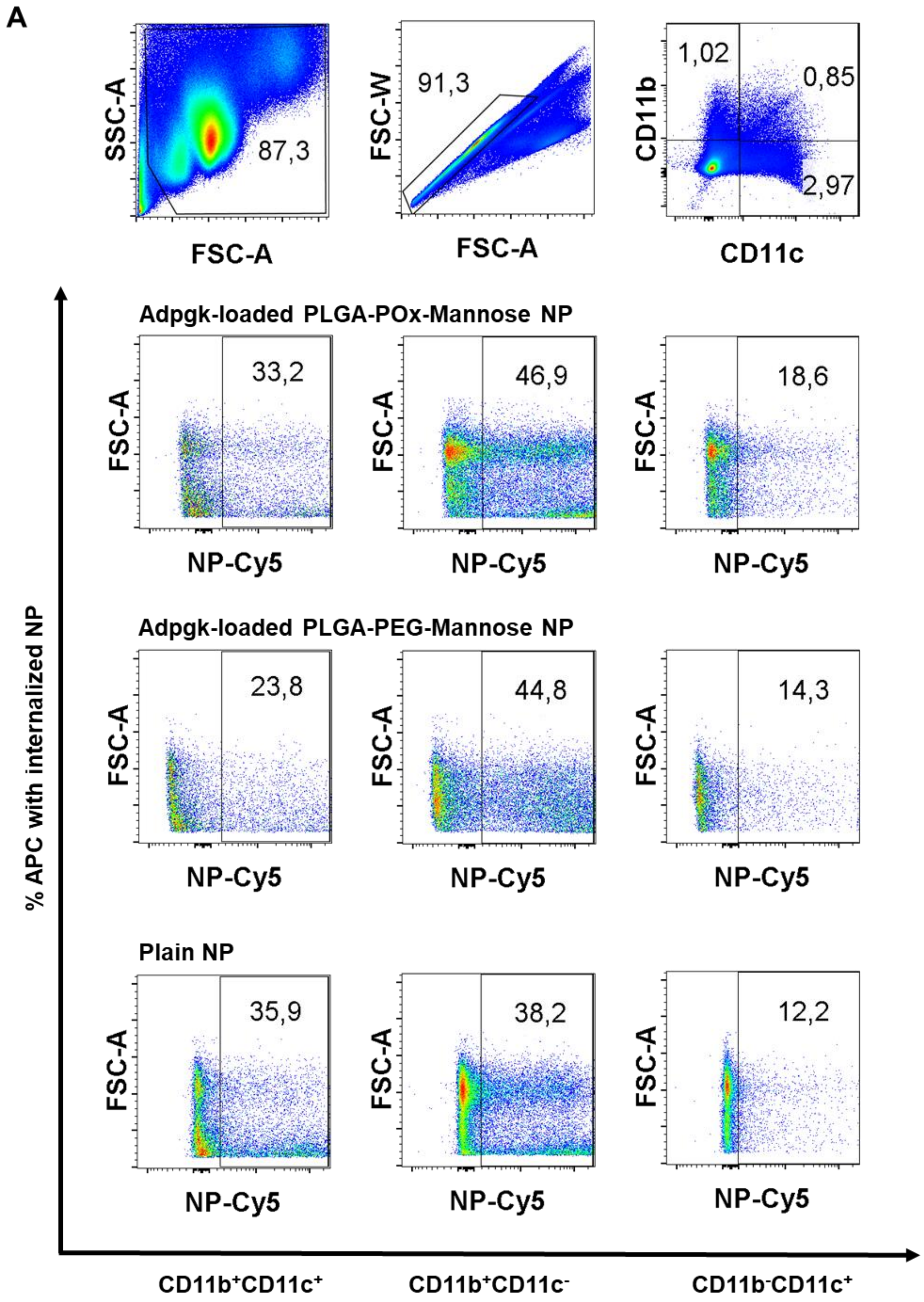
A
SSC-A
FSC-A
87,3
FSC-W
FSC-A
91,3
CD11b
CD11c
1,02
0,85
2,97
Adpgk-loaded PLGA-POx-Mannose NP
FSC-A
NP-Cy5
33,2
46,9
18,6
Adpgk-loaded PLGA-PEG-Mannose NP
23,8
44,8
14,3
Plain NP
35,9
38,2
12,2
% APC with internalized NP
CD11b+CD11c+
CD11b+CD11c-
CD11b-CD11c+

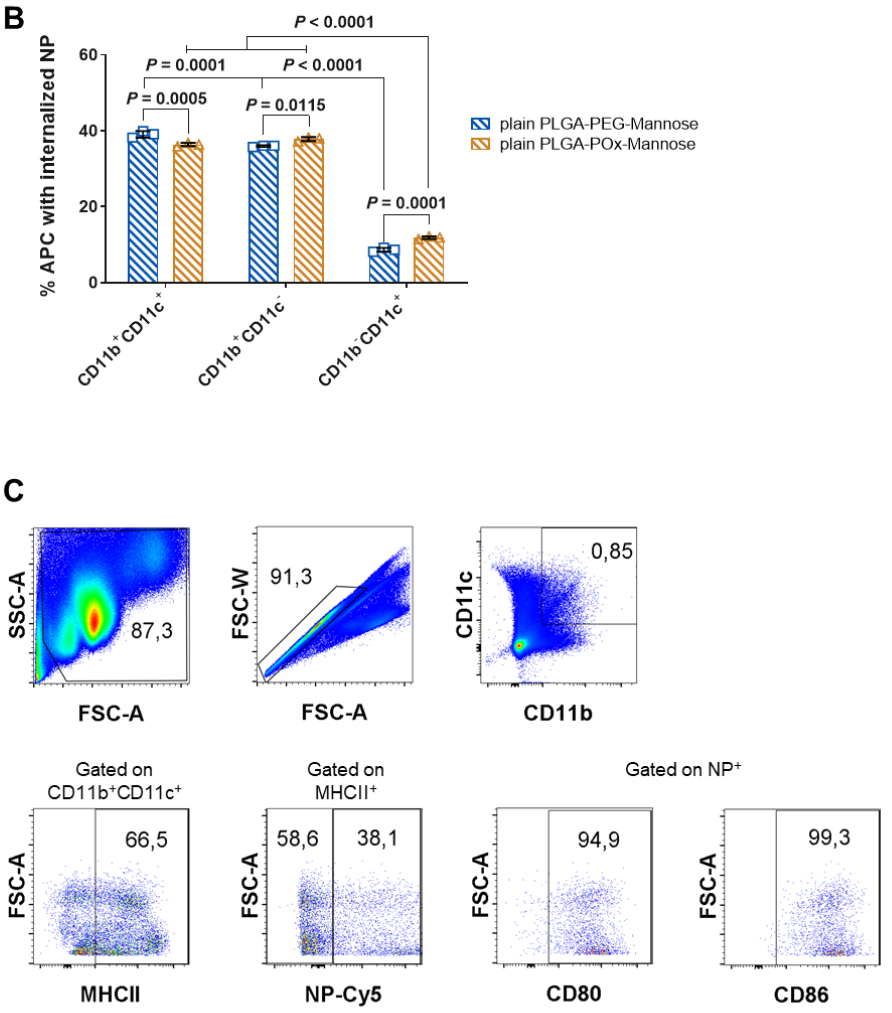

Figure S3. Favored internalization of Adpgk-loaded PLGA-POx-Mannose NP by APC. Lymph nodes of C57BL/6J mice (*n* = 3) were collected 14 h after immunization with plain and Adpgk-loaded PLGA-POx-Man or PLGA-PEG-Man NP. (**A**) Representative gating strategy and scatter plots for a percentage of plain and antigen-loaded PLGA-POx-Man or PLGA-PEG-Man NP internalized by APC, as analyzed by CD11b and CD11c staining. (**B**) Percentage of CD11b$^+$CD11c$^+$, CD11b$^+$CD11c$^-$, and CD11b$^-$CD11c$^+$ cells, with internalized plain PLGA-PEG-Man or PLGA-POx-Man NP, 14 h after immunization. The data show mean ± s.d. Statistical significance was calculated by two-way analysis of variance (ANOVA) with Tukey multiple comparisons post-hoc test. (**C**) Representative gating strategy for median fluorescence intensity (MFI) of activation and maturation markers on the surface of activated circulating DC that internalized and did not internalize antigen-loaded NP, as analyzed by CD11b, CD11c, MHCII, CD80, and CD86 staining.

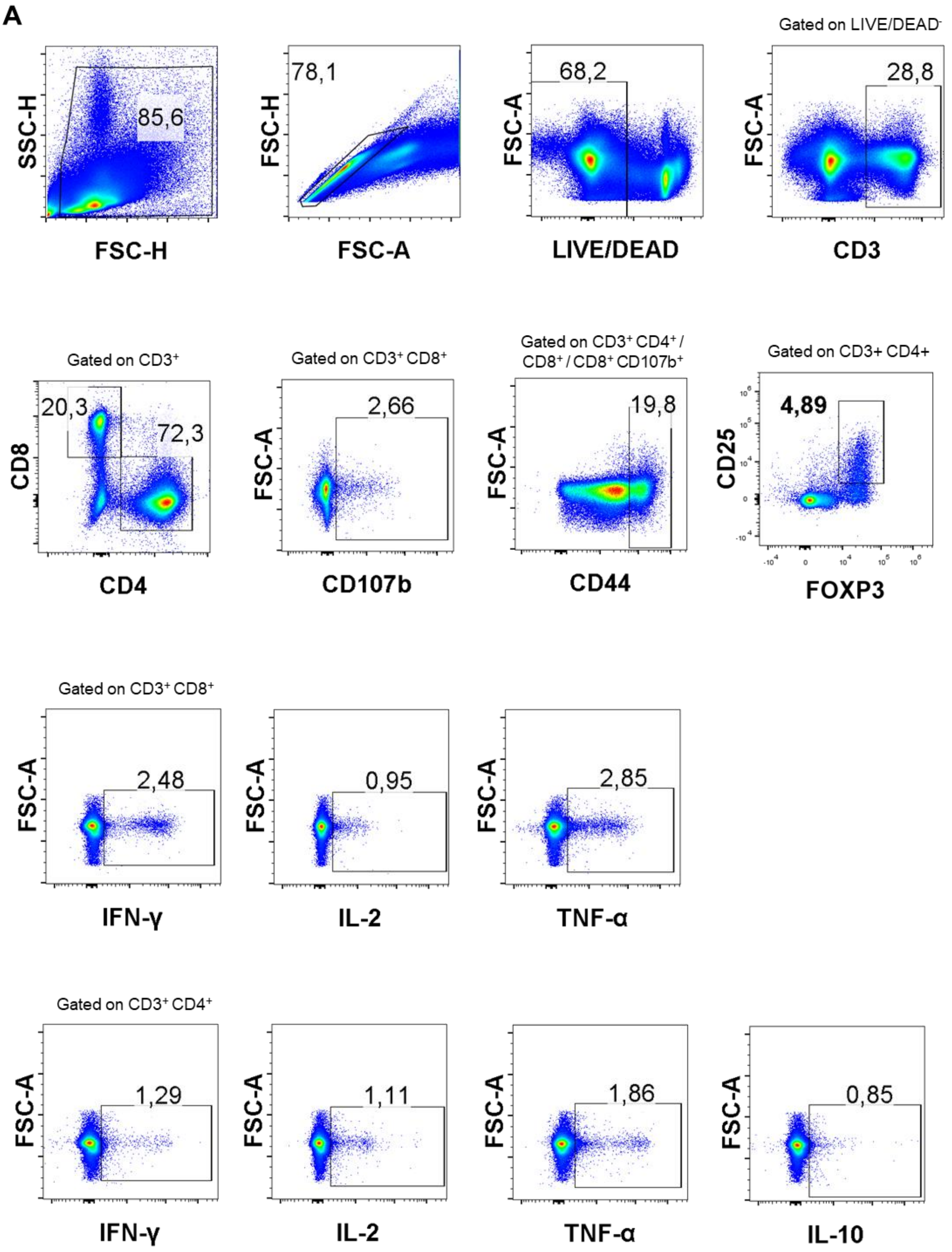
A
Gated on LIVE/DEAD-
85,6
SSC-H
FSC-H
78,1
FSC-H
FSC-A
68,2
FSC-A
LIVE/DEAD
28,8
FSC-A
CD3
Gated on CD3+
20,3
72,3
CD8
CD4
Gated on CD3+ CD8+
2,66
FSC-A
CD107b
Gated on CD3+ CD4+ / CD8+ / CD8+ CD107b+
19,8
FSC-A
CD44
Gated on CD3+ CD4+
4,89
CD25
FOXP3
Gated on CD3+ CD8+
2,48
FSC-A
IFN-γ
0,95
FSC-A
IL-2
2,85
FSC-A
TNF-α
Gated on CD3+ CD4+
1,29
FSC-A
IFN-γ
1,11
FSC-A
IL-2
1,86
FSC-A
TNF-α
0,85
FSC-A
IL-10

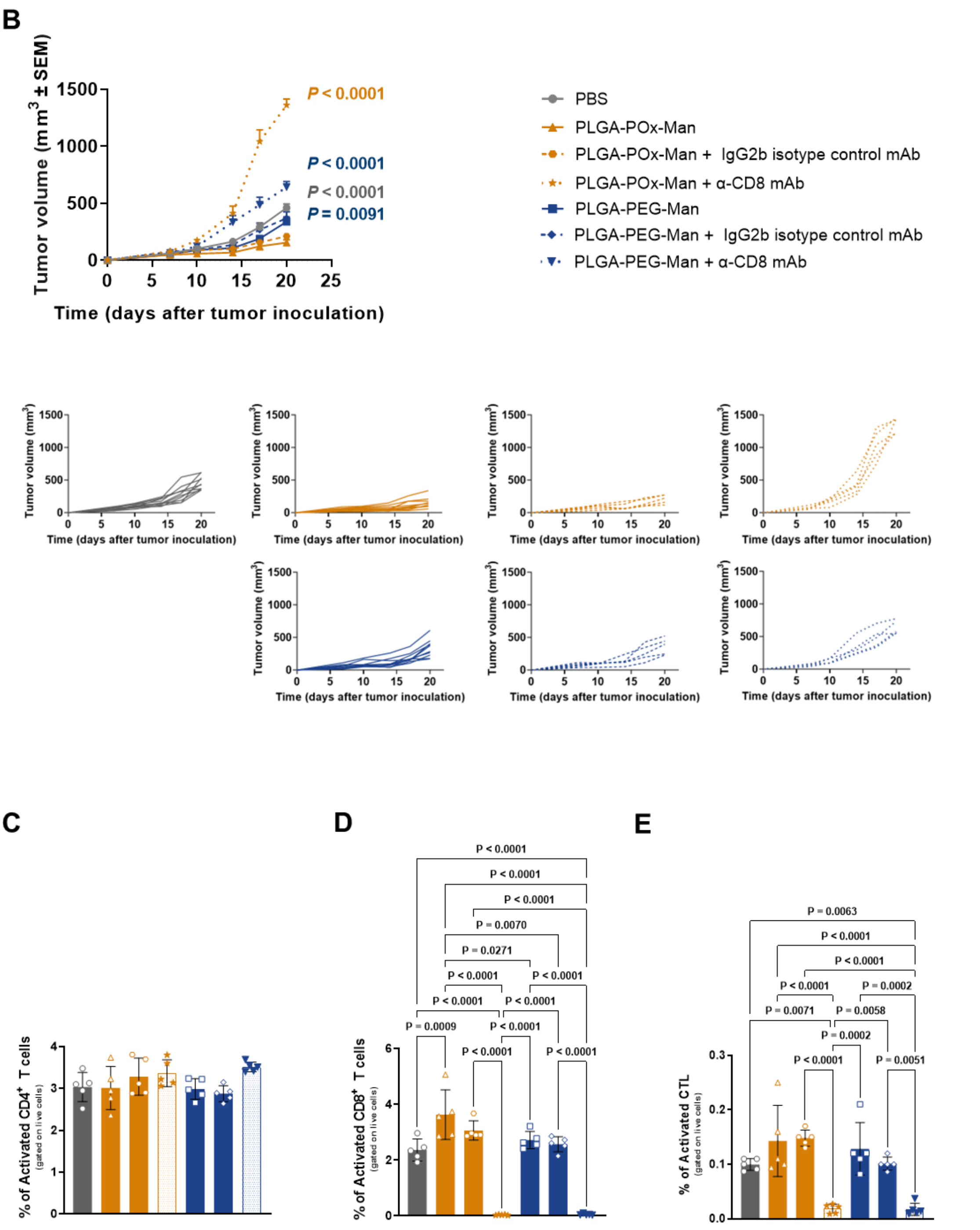
B
Tumor volume (mm³ ± SEM)
Time (days after tumor inoculation)
P < 0.0001
P < 0.0001
P < 0.0001
P = 0.0091
PBS
PLGA-POx-Man
PLGA-POx-Man + IgG2b isotype control mAb
PLGA-POx-Man + α-CD8 mAb
PLGA-PEG-Man
PLGA-PEG-Man + IgG2b isotype control mAb
PLGA-PEG-Man + α-CD8 mAb
Tumor volume (mm³)
Time (days after tumor inoculation)
C
% of Activated CD4⁺ T cells (gated on live cells)
D
% of Activated CD8⁺ T cells (gated on live cells)
E
% of Activated CTL (gated on live cells)

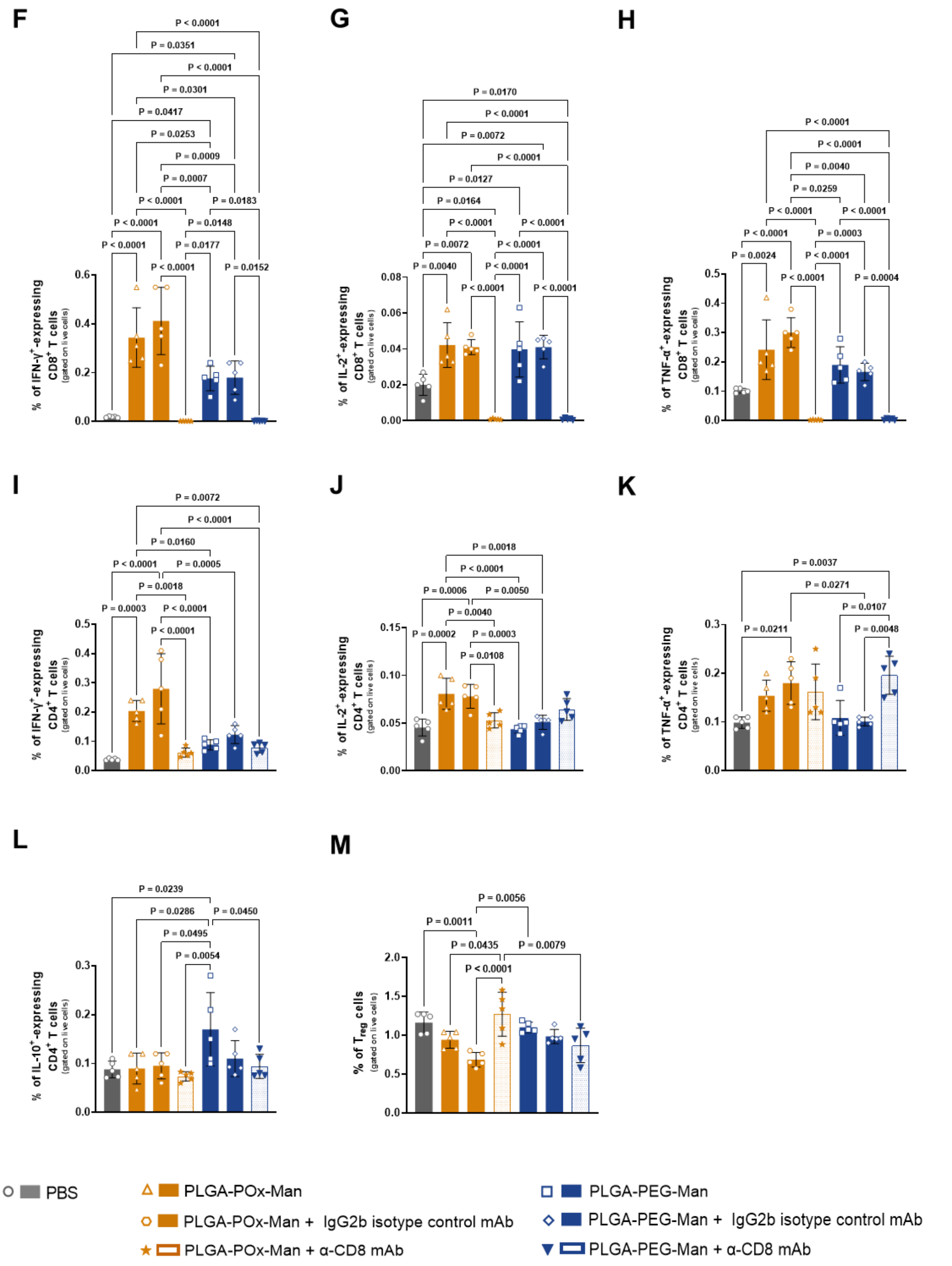

Figure S4. The co-delivery of antigens and adjuvants by POx-Man nanovaccines strongly inhibits tumor growth in MC38-bearing mice by enhancing the systemic activation of T lymphocytes and the secretion of Th1 cytokines, being strongly mediated by CD8$^+$ T cells. (**A**) Representative gating strategy used for the activated CD4/CD8 T cells/CTL (CD3$^+$ CD4$^+$/CD8$^+$/CD8$^+$ CD107b$^+$ CD44$^+$) and T regulatory (Treg) cells (CD3$^+$ CD4$^+$ CD25$^+$ Foxp3$^+$), and cytokines-expressing CD4/CD8 T cells (CD4/CD8 Th1: CD3$^+$ CD4$^+$/CD8$^+$ IFN-γ$^+$/IL-2$^+$/TNF-α$^+$ and CD4 Th2: CD3$^+$ CD4$^+$ IL-10$^+$) analyzed in spleen cell suspensions after *ex vivo* re-stimulation with

Adpgk MHCI/MHCII peptides for 6 h (*n* = 3-5 animals/group). (**B**) C57BL/6J mice were inoculated subcutaneously with 0.5x10^6 MC38 tumor cells and treated with Adpgk-loaded PEG-Man and POx-Man nanovaccines on days 7 and 14 in combination with αCD8 or IgG2b isotype control monoclonal antibodies (mAb) (10 mg kg^{-1}), on days 6, 9, 12 and 15. Average and individual MC38 tumor growth curves. The data are presented as mean ± s.e.m of MC38-bearing mice (*n* = 5 animals), replicated in two independent experiments for PBS, PLGA-POx-Man, and PLGA-PEG-Man groups. Statistical significance was analyzed by one-way analysis of variance (ANOVA) followed by Tukey multiple comparisons post-hoc test and *P* values correspond to tumor volume at day 20 after tumor inoculation, compared to the PLGA-POx-Man nanovaccine group. (**C** to **E**) The activation of CD4 (**C**), CD8 (**D**), and CTL (**E**) is enhanced by the co-delivery of antigens and adjuvants by POx-Man nanovaccine. (**F** to **M**) Secretion of IFN-γ (**F** and **I**), IL-2 (**G** and **J**), TNF-α (**H** and **K**), and IL-10 (**L**) by $CD8^+$ and $CD4^+$ T cells after re-stimulation of splenocytes in culture with relevant peptides for 6 h. The highest levels of the triad IFN-γ, IL-2, and TNF-α induced by Adpgk-loaded POx-Man nanovaccine predict an improved cytotoxic $CD8^+$/Th1 T-cell activity. This nanovaccine also modulated the Th2 cytokine secretion profile, while inducing lower levels of $CD4^+$ T cells expressing IL-10 and Treg (**M**) cells when compared to PEG-Man nanovaccine. The data are presented as the mean ± s.d., *n* = 5. Statistical significance was calculated by one-way ANOVA followed by Tukey multiple comparisons post-hoc test.

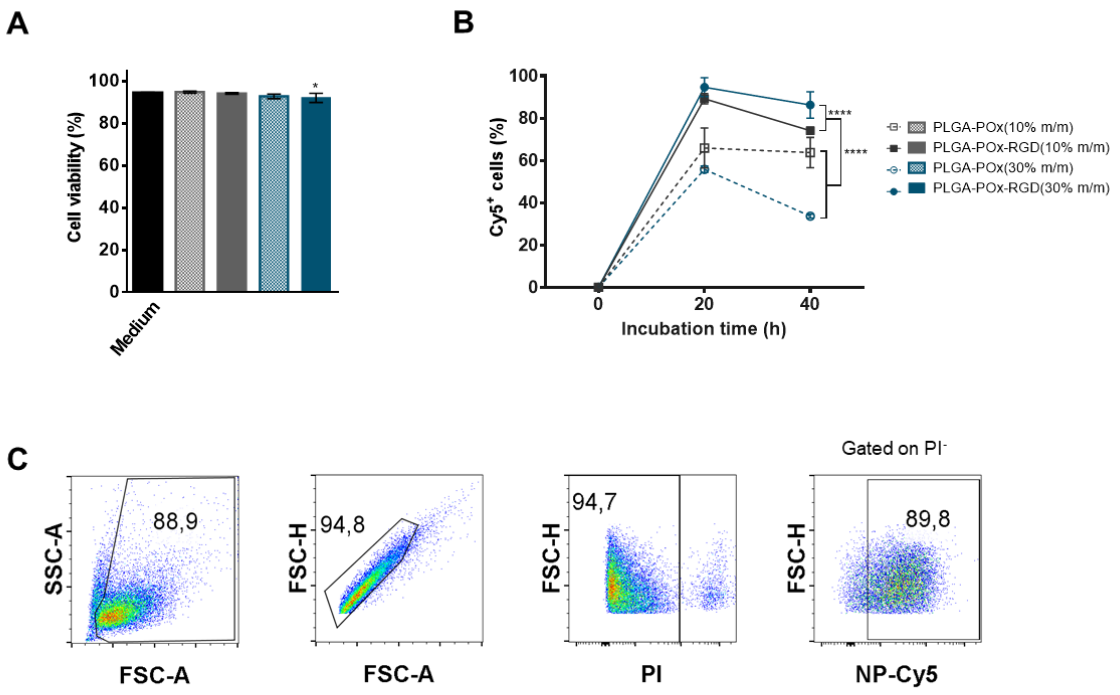

Figure S5. RGD-functionalized NP are safe and readily internalized by MC38 cells. Flow cytometry analysis of cell viability (**A**) and NP internalization (**B**) after incubation of RGD-functionalized POx NP, at different percentages of RGD, incubated with MC38 cells, for 40 h. Non-treated cells and non-targeted (no targeting moiety) NP were used as controls. Data are presented as mean ± s.d., $N = 1$, independent experiments (cell line passages) performed with triplicates of NP ($n = 3$), with three technical replicates per NP. *$P < 0.05$, ****$P < 0.0001$, analyzed by one-way (**A**) two-way (**B**) analysis of variance (ANOVA) with Tukey multiple comparisons post-hoc test. (**C**) Representative gating strategy used for (**A**)**,** cell viability (PI$^-$) and (**B**)**,** NP internalization (fluorescent NP-Cy5$^+$ (APC-Cy7 channel)) analysis.

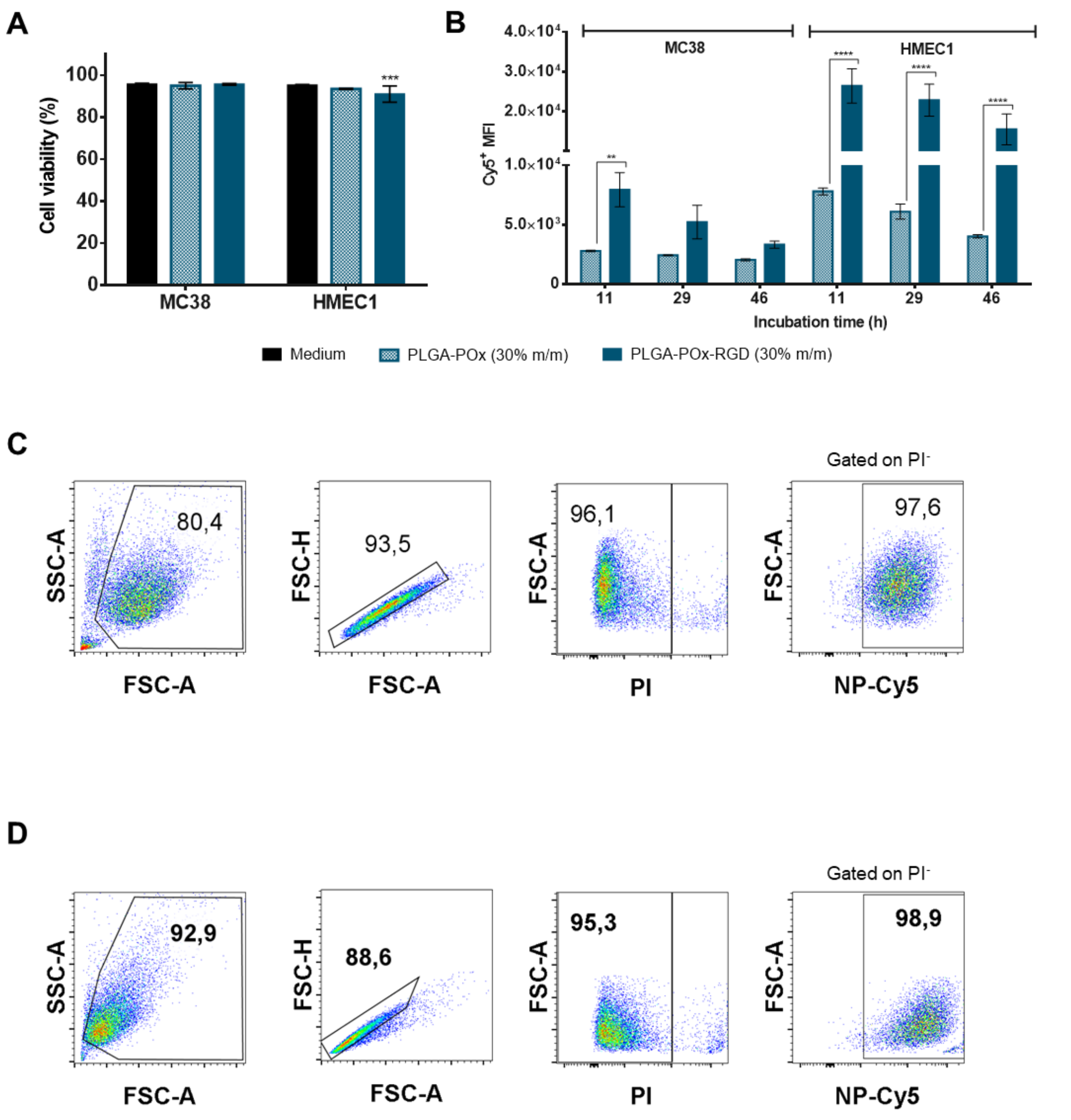

Figure S6. Internalization of TIME-targeted NP is mediated by RGD receptors. Flow cytometry analysis of cell viability (**A**) and NP internalization (**B**) after incubation of PLGA-POx-RDG (30% m/m) NP incubated with MC38 and HMEC1 ($\alpha_v\beta_3^+\alpha_v\beta_5^+$) dermal microvascular endothelial cells, for 46 hours. Non-treated cells and non-targeted (no targeting moiety) NP were used as controls. Data are presented as mean ± s.d., $N = 1$, independent experiments (cell line passages) performed with duplicates and quadruplicates ($n = 2\text{-}4$) of non-targeted and RGD-targeted NP, respectively, with three technical replicates per NP. $^{**}P < 0.01$, $^{***}P < 0.001$, $^{****}P < 0.0001$, analyzed by two-way analysis of variance (ANOVA) with Tukey multiple comparisons post-hoc test. Representative gating strategy used for MC38 (**C**) and HMEC1 ($\alpha_v\beta_3^+\alpha_v\beta_5^+$) dermal microvascular endothelial (**D**) cells regarding (**A**)**,** cell viability (PI$^-$) and (**B**)**,** NP internalization (fluorescent NP-Cy5$^+$ (APC-Cy7 channel)) analysis.

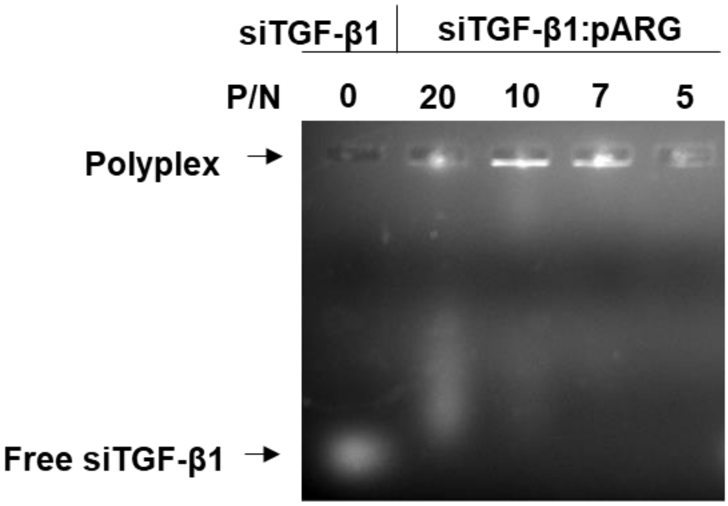

Figure S7. Electrophoretic mobility shift assay determine the optimal P/N ratio of 7 for polyplex establishment. Electrostatic-based polyplex formation of siTGF-β1 (total of 50 pmol) with pARG, at different Phospate/Nitrogen (P/N) ratios tested in RNAse free water. Free siTGF-β1 was used as a control.

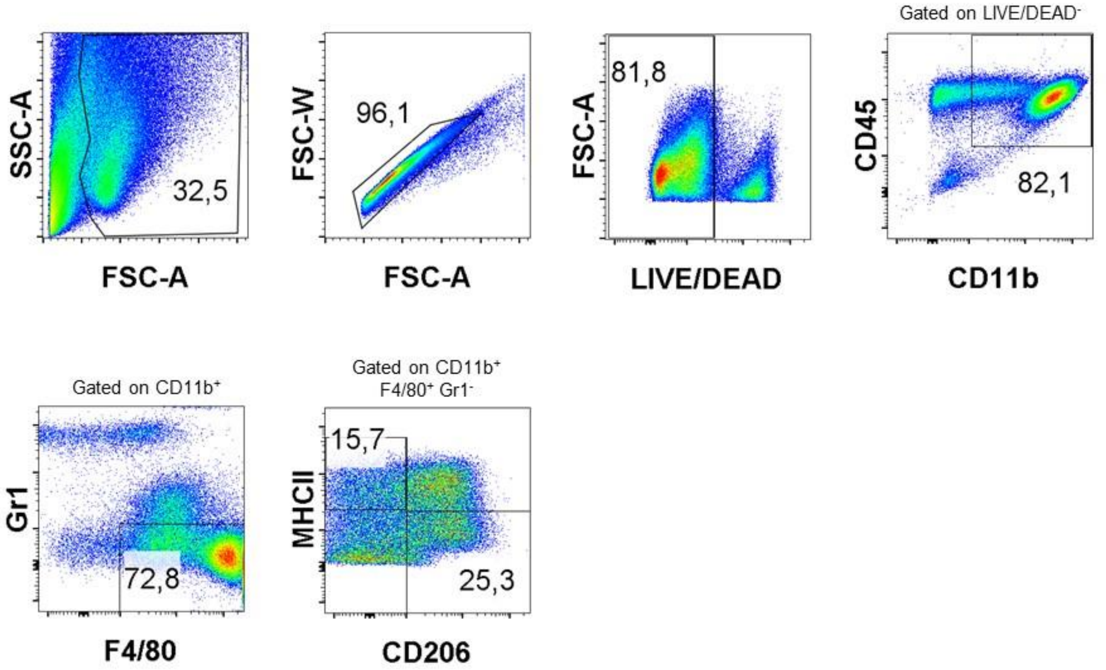

Figure S8. Intracellular staining of tumor-associated macrophages subsets. Representative gating strategy used for CD45^{+} CD11b^{+} F4/80^{+} Gr1^{-} MHCII^{+} CD206^{-} TAM (M1-like TAM) and CD45^{+} CD11b^{+} F4/80^{+} Gr1^{-} MHCII^{-} CD206^{+} TAM (M2-like TAM) analyzed in tumor cell suspensions (n = 3 animals/group).

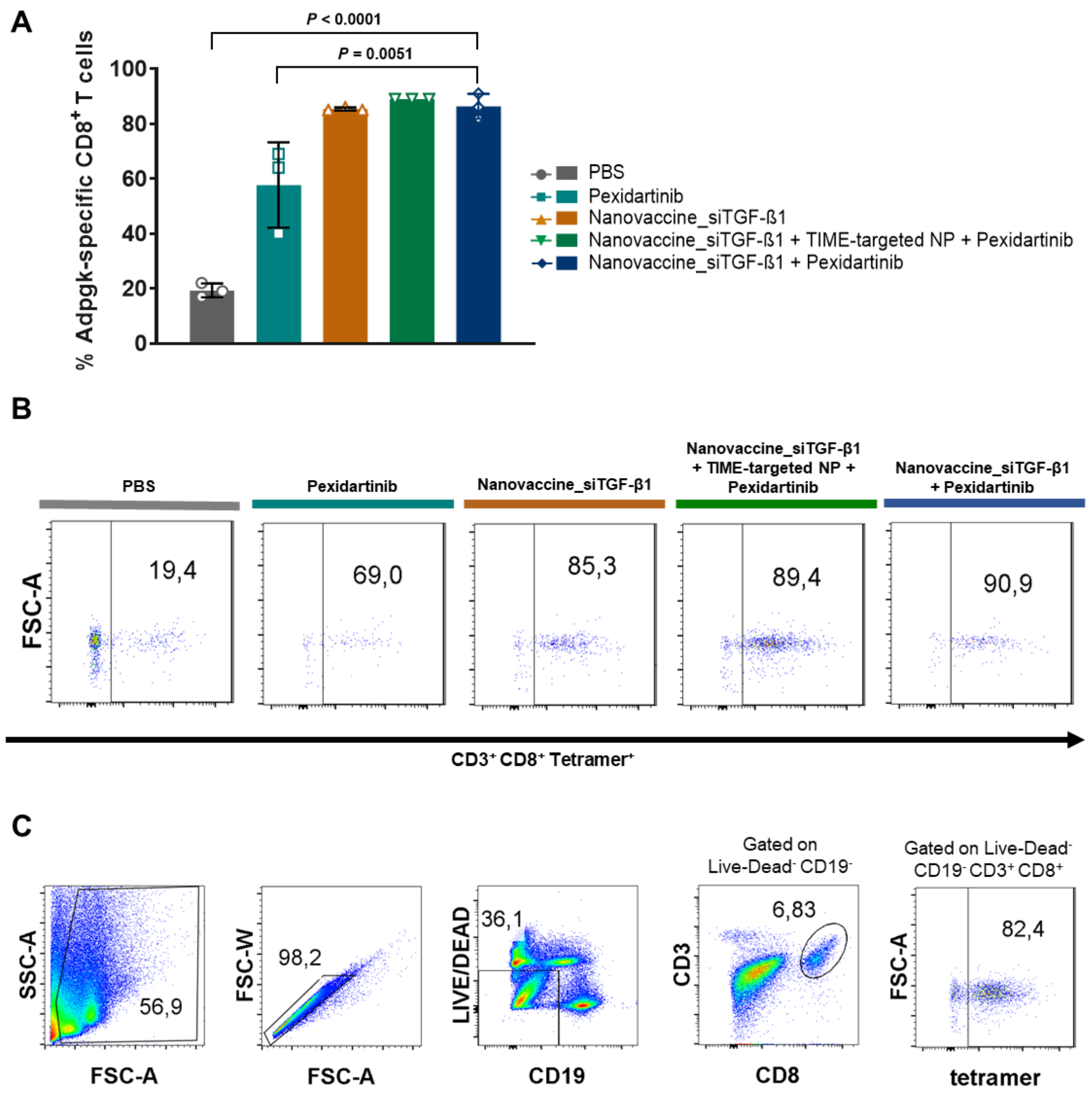

Figure S9. Divalent combination of the therapeutic siTGF-β1-loaded POx-Man nanovaccine with TAM modulation induces Adpgk antigen-specific immune responses. (**A**) Frequency of Adpgk-specific CD8$^+$ T cells in the spleen. Data are presented as mean ± s.d., *n* = 3 animals. Statistical significance was calculated by one-way ANOVA followed by the Tukey post-hoc test. (**B**) Representative scatter plots for Adpgk-specific CD8$^+$ T cells in the spleen. (**C**) Representative gating strategy used for the Adpgk-specific CD8$^+$ T cells analyzed in spleen cell suspensions (*n* = 3 animals/group).

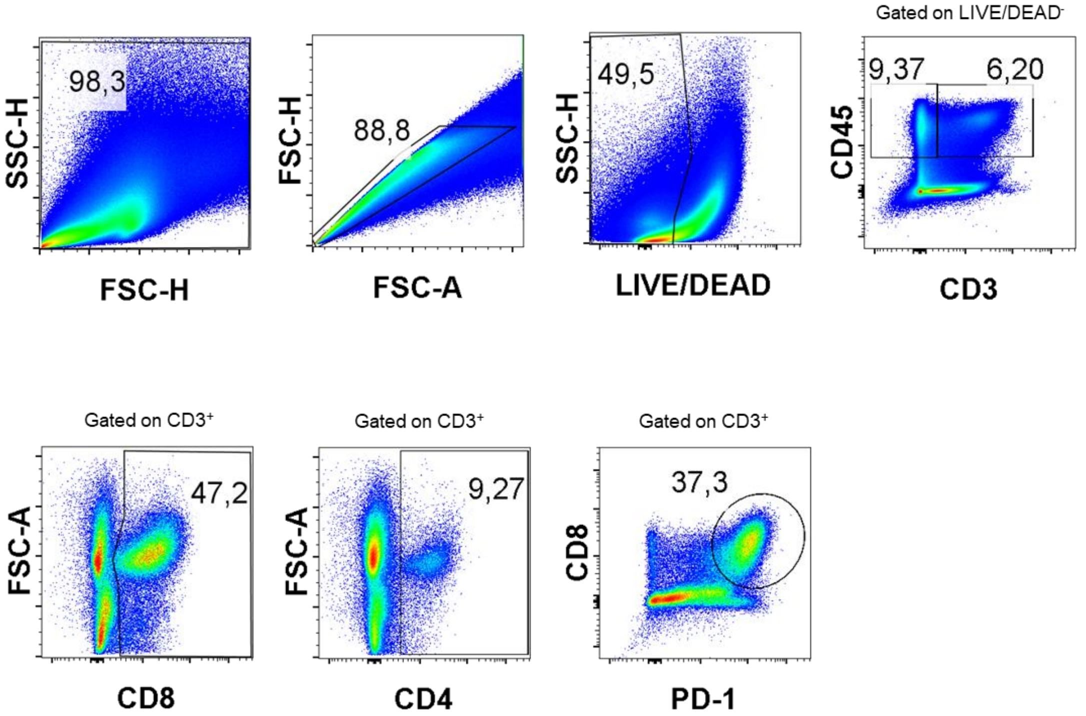

Figure S10. Cell surface staining of tumor-infiltrating lymphocyte and natural killer subsets. Representative gating strategy used for CD3+ (CD45+ CD3+), CD8+ (CD45+ CD3+ CD8+), PD-1+-expressing CD8+ (CD45+ CD3+ CD8+ PD-1+) and CD4+ (CD45+ CD3+ CD4+) T cells analyzed in tumor cell suspensions (*n* = 5 animals/group).

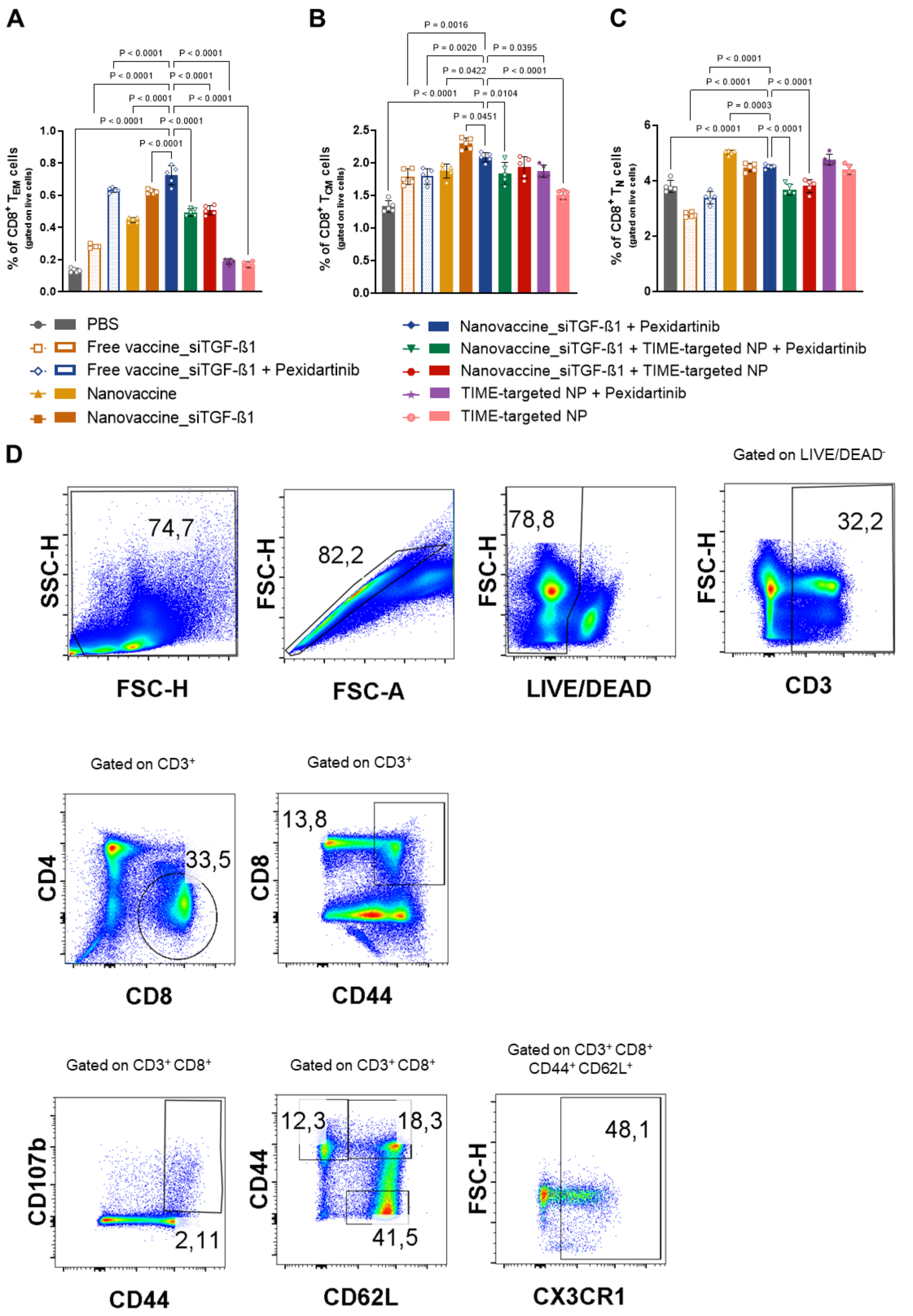

Figure S11. Divalent combination of the therapeutic siTGF-β1-loaded POx-Man nanovaccine with TAM modulation trigger a systemic activation of CD8$^+$ T memory. (**A** to **C**) Percentage of systemic (**A**) activated CD8$^+$ T effector memory (T_{EM}), (**B**) CD8$^+$ T central memory (T_{CM}), and (**C**) CD8$^+$ T naïve memory (T_N) cells. Spleens recovered on day 19 following tumor inoculation. The quantification was performed by flow cytometry analysis. Data are

presented as mean ± s.d., *n* = 5 animals. Statistical significance was calculated by one-way ANOVA with Dunnett multiple comparisons post-hoc test. (**D**) Representative gating strategy used for activated $CD8^+$ ($CD3^+$ $CD8^+$ $CD44^+$), activated CTL ($CD3^+$ $CD8^+$ $CD107b^+$ $CD44^+$), activated $CD8^+$ T effector memory (T_{EM}) ($CD3^+$ $CD8^+$ $CD44^+$ $CD62L^-$ $CX3CR1^+$), $CD8^+$ T central memory (T_{CM}) ($CD3^+$ $CD8^+$ $CD44^+$ $CD62L^+$), and $CD8^+$ T naïve memory (T_N) ($CD3^+$ $CD8^+$ $CD44_-$ $CD62L^+$) cells analyzed in spleen cell suspensions (*n* = 5 animals/group).

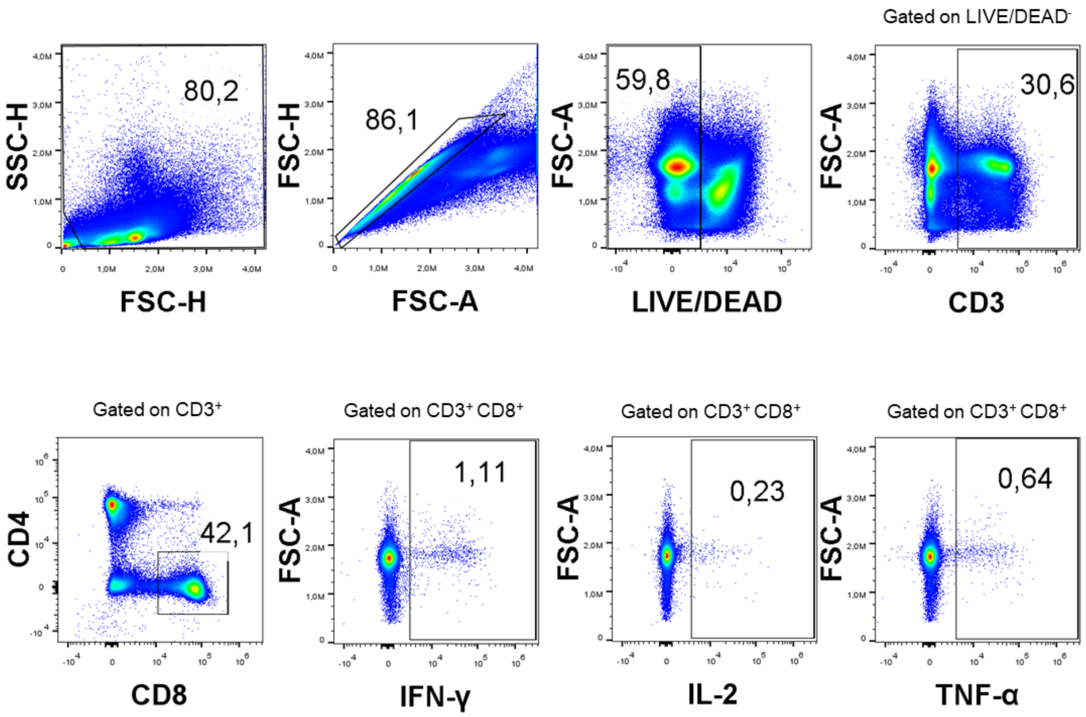

Figure S12. Intracellular staining of systemic Th1 cytokine expressing CD8$^+$ T cells. Representative gating strategy used for systemic IFN-γ$^+$-expressing CD8$^+$ (CD3$^+$ CD8$^+$ IFN-γ$^+$), IL-2$^+$-expressing CD8$^+$ (CD3$^+$ CD8$^+$ IL-2$^+$), and TNF-α$^+$-expressing CD8$^+$ (CD3$^+$ CD8$^+$ TNF-α$^+$) T cells analyzed in spleen cell suspensions after *ex vivo* re-stimulation with Adpgk MHCI/MHCII peptides for 6 h (n = 5 animals/group).

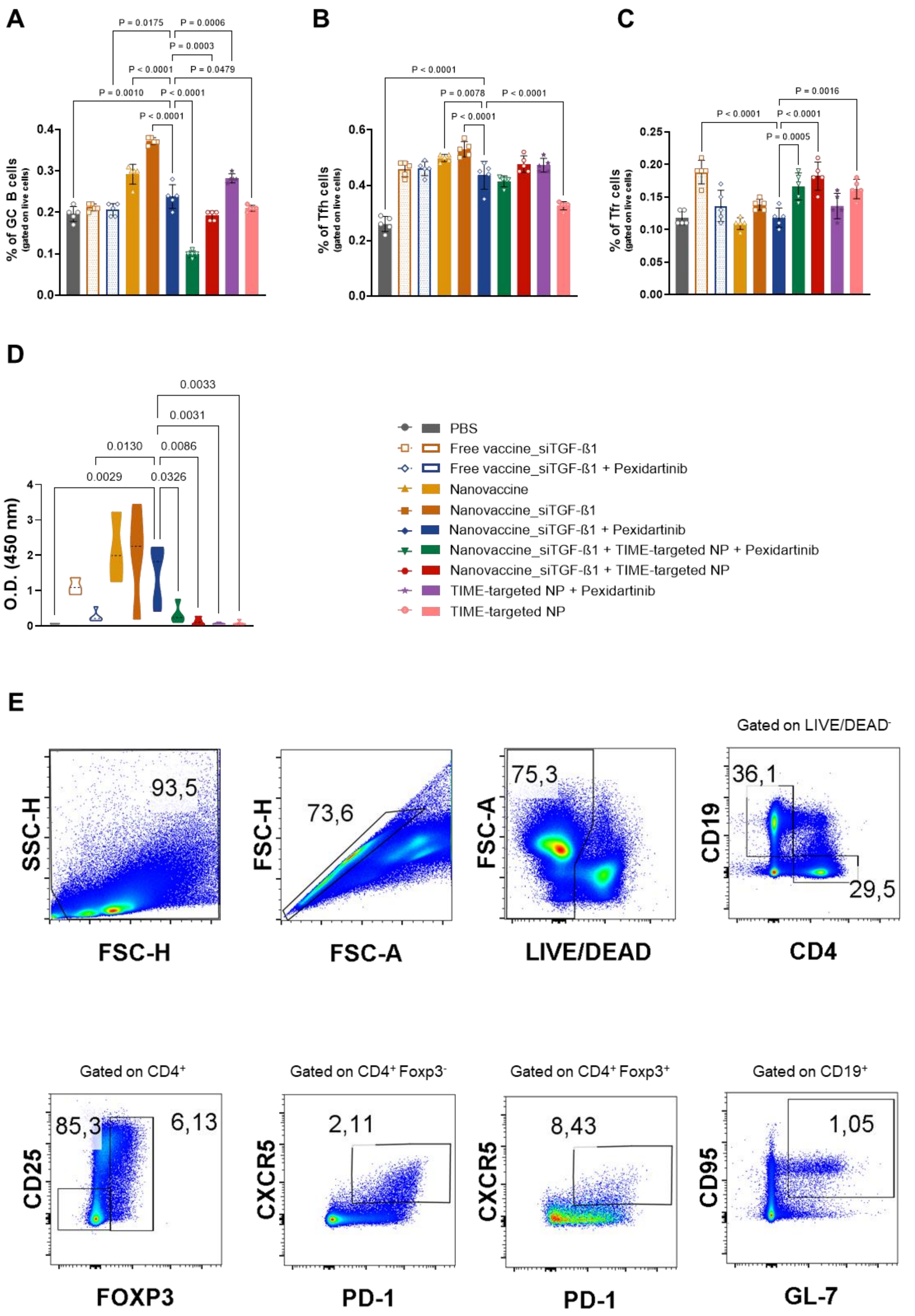

Figure S13. Increased amount of germinal center (GC) B and T follicular cells in the LN and antibody secretion for siTGF-β1-loaded POx-Man nanovaccine + Pexidartinib. (**A** to **C**) Frequency of germinal center (GC) B (**A**), T follicular helper (Tfh) (**B**), and T follicular regulatory (Tfr) (**C**) cells in the inguinal LN of MC38-bearing mice. LN recovered on day 19 following tumor inoculation. The quantification was performed by flow cytometry analysis. Data are presented as

mean ± s.d., n = 5 animals. Statistical significance was calculated by one-way ANOVA with Dunnett multiple comparisons post-hoc test. (**D**) Adpgk MHCII peptide-specific IgG antibody determined by enzyme-linked immunosorbent assay (ELISA) in mice serum (serum dilution 1:135) 19 days following tumor inoculation. Violin plots represent the median and quartiles. Number of animals / group: PBS=5, Free vaccine_siTGF-β1=3, Free vaccine_siTGF-β1 + Pexidartinib=5, Nanovaccine=3, Nanovaccine_siTGF-β1=4, Nanovaccine_siTGF-β1 + Pexidartinib=5, Nanovaccine_siTGF-β1 + TIME-targeted NP + Pexidartinib=4, Nanovaccine_siTGF-β1 + TIME-targeted NP=4, TIME-targeted NP + Pexidartinib=5, and TIME-targeted NP=5. Statistical significance was calculated by one-way ANOVA with Dunnett multiple comparisons post-hoc test. (**E**) Representative gating strategy used for the GC B cells ($CD19^+$ $CD95^+$ $GL\text{-}7^+$), Tfh ($CD4^+$ $Foxp3^-$ $CXCR5^+$ $PD\text{-}1^+$) and Tfr ($CD4^+$ $Foxp3^+$ $CXCR5^+$ $PD\text{-}1^+$) analyzed in LN cell suspensions (n = 5 animals/group).

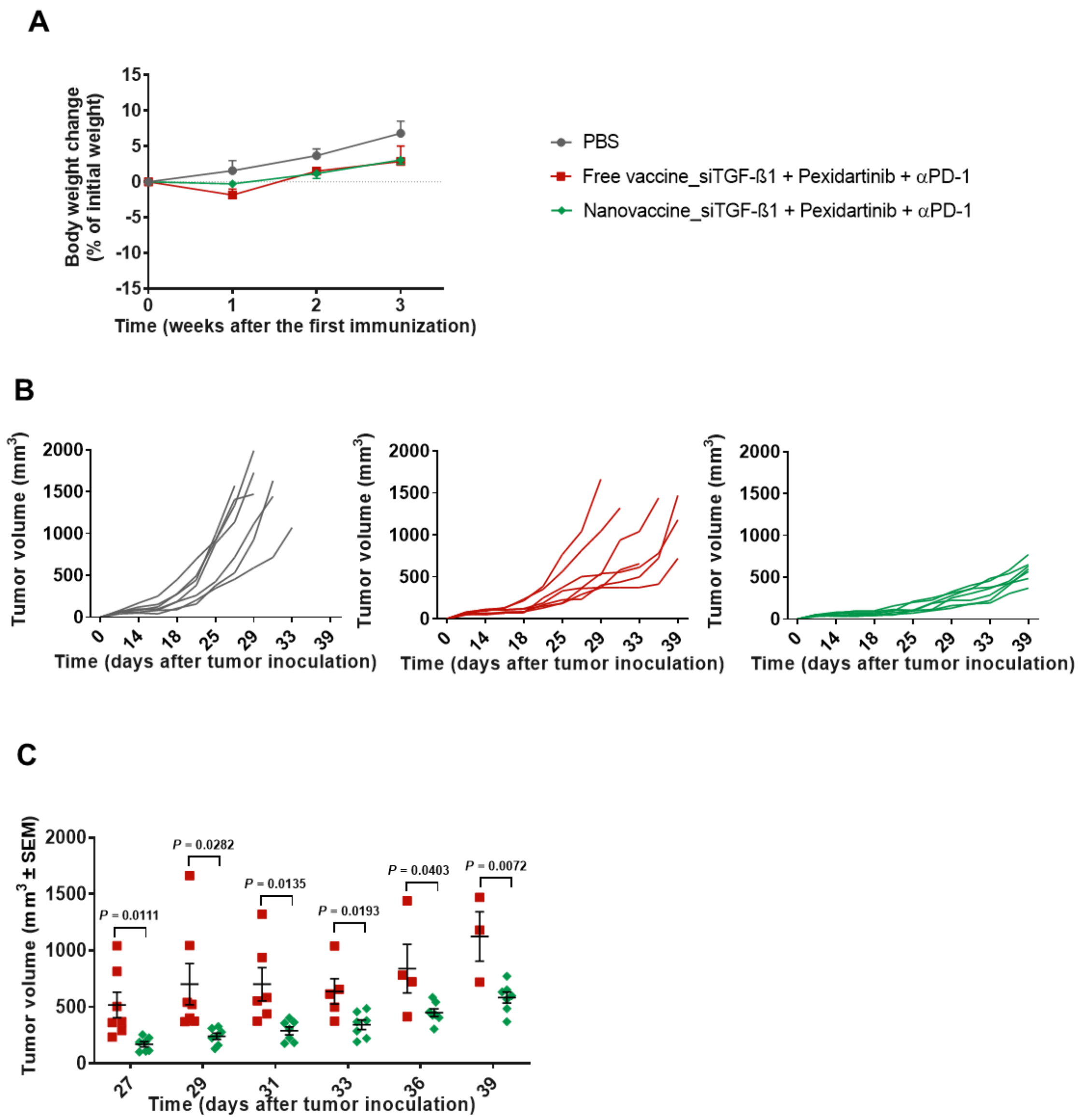

Figure S14. Trivalent combination of POx-Man Nanovaccine_siTGF-β1, Pexidartinib and αPD-1 strongly restricts MC38 tumors growth and leads to long-term survival. (**A**) Body weight change is expressed as the percent change in weight from the day of treatment initiation. Data are presented as mean ± s.e.m of MC38-bearing mice (*n* = 7 animals). (**B**) Individual MC38 tumor growth curves. (**C**) Individual MC38 tumor volumes at days 27, 29, 31, 33, 36, and 39 following tumor inoculations. Data are presented as mean ± s.e.m of MC38-bearing mice (*n* = 7 animals). Statistical significance was analyzed by multiple t tests followed by Holm-Sidak method.

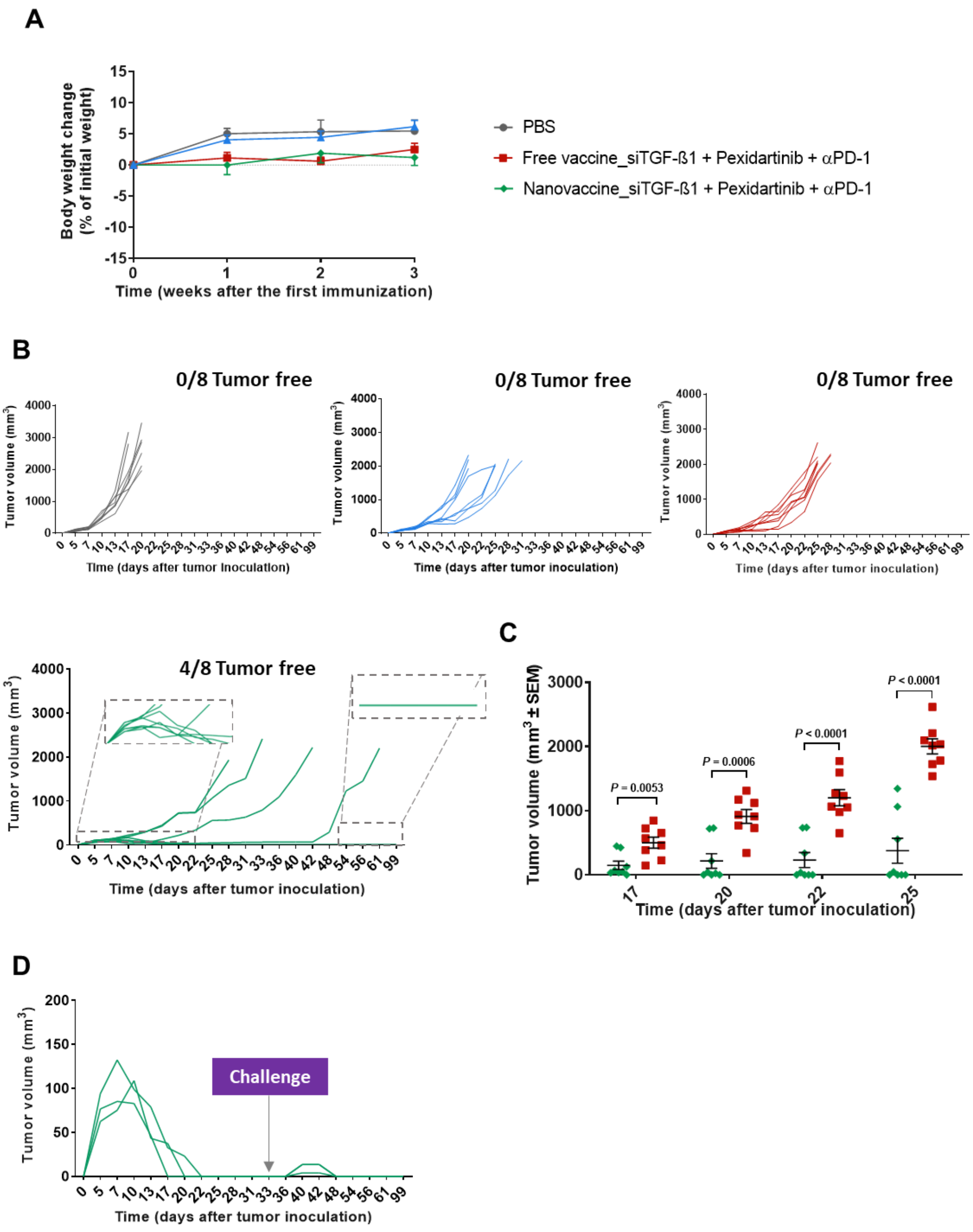

Figure S15. Trivalent combination of POx-Man Nanovaccine_siTGF-β1, Pexidartinib, and αPD-1 is also effective in CT26-bearing mice. (**A**) Body weight change is expressed as the percent change in weight from the day of treatment initiation. Data are presented as mean ± s.e.m of CT26-bearing mice (*n* = *8* animals). (**B**) Individual CT26 tumor growth curves. Mice treated with POx-Man Nanovaccine_siTGF-β1 in combination with both Pexidartinib and αPD-1 showed a robust response, with 4/8 mice showing complete tumor shrinkage. (**C**) Individual CT26 tumor volumes at days 17, 20, 22 and 25 following tumor inoculation. Data are presented as mean ± s.e.m of CT26-bearing mice (*n* = *8* animals). Statistical significance was analyzed by multiple t tests followed by Holm-Sidak method. (**D**) Individual CT26 tumor growth curves of C57BL/6J responders (Nanovaccine_siTGF-β1 + Pexidartinib + αPD-1) subcutaneously challenged with 0.5x10^6 CT26 tumor cells at the left flank, on day 33.

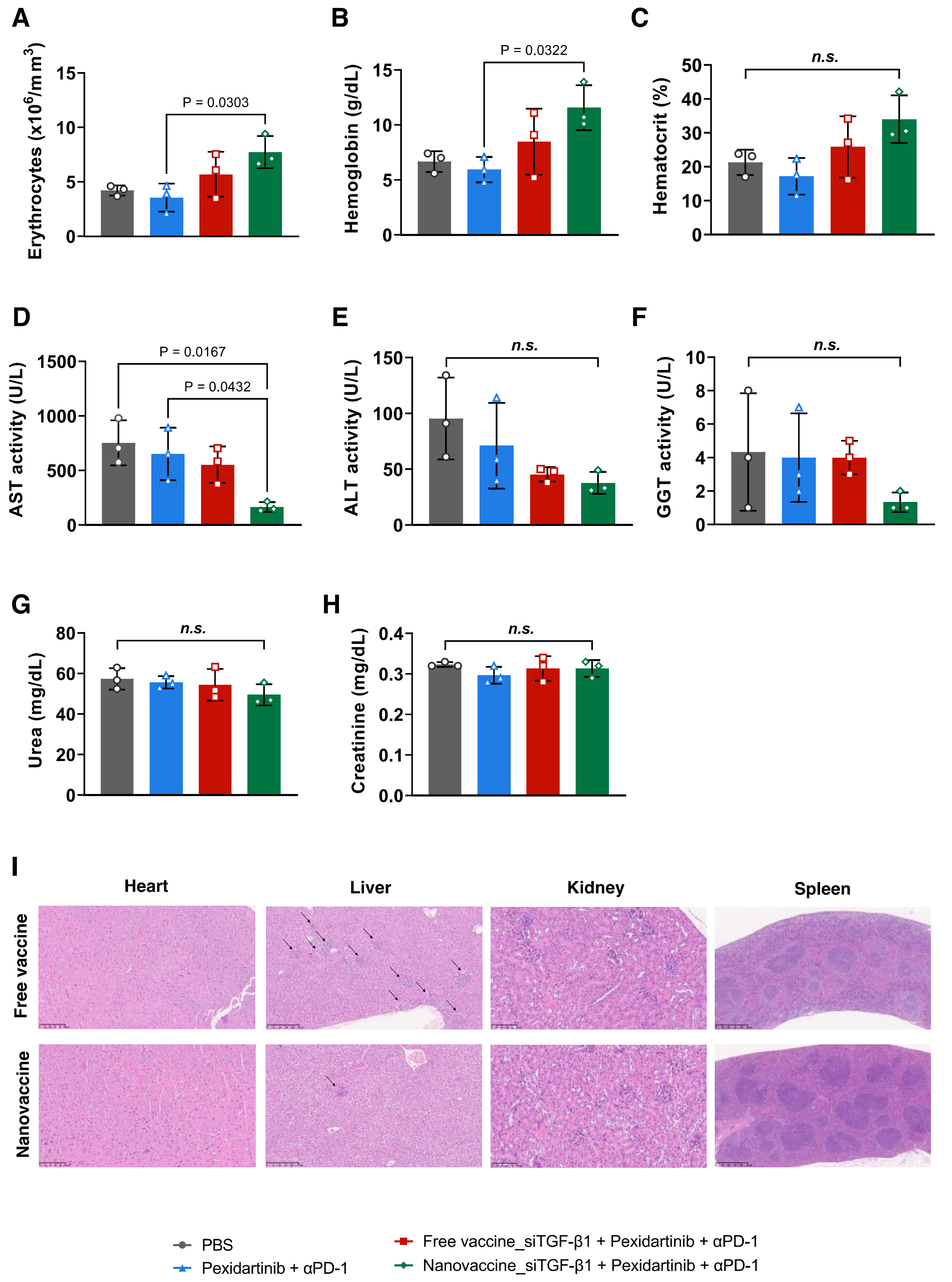

Figure S16. Trivalent combination of POx-Man Nanovaccine_siTGF-β1, Pexidartinib, and αPD-1 induced a superior anti-tumor effect against aggressive B16F10 melanoma with no acute organ toxicity. (**A to C**) Hematological analysis of erythrocytes (**A**), hemoglobin (**B**), and hematocrit (**C**) levels in blood from animals of PBS, Pexidartinib + αPD-1, Free vaccine_siTGF-β1 + Pexidartinib + αPD-1, and Nanovaccine_siTGF-β1 + Pexidartinib + αPD-1 groups. (**D to H**) Biochemical analysis of AST (**D**), ALT (**E**), and GGT (**F**) activities, as well as urea (**G**) and creatinine (**H**) levels in blood from animals of all treatment groups. Data are presented as mean

± s.e.m of B16F10-bearing mice (*n = 3* animals). Statistical significance was analyzed by one-way analysis of variance (ANOVA) followed by Tukey multiple comparisons post-hoc test (n.s.: not significant). (**I**) Representative histopathology images of hematoxylin and eosin staining of organs (heart (20X), liver (10X), kidney (20X), and spleen (5X)) recovered from animals treated with Free vaccine_siTGF-β1 + Pexidartinib + αPD-1 and Nanovaccine_siTGF-β1 + Pexidartinib + αPD-1. No significant alterations (within normal limits) were observed in the heart, kidney, and spleen. In contrast, multifocal foci of inflammatory cell infiltration (mononuclear) associated with moderate liver necrosis were found in the liver of animals treated with the Free vaccine_siTGF-β1 + Pexidartinib + αPD-1.
